# Multiple major disease-associated clones of *Legionella pneumophila* have emerged recently and independently

**DOI:** 10.1101/gr.209536.116

**Published:** 2016-11

**Authors:** Sophia David, Christophe Rusniok, Massimo Mentasti, Laura Gomez-Valero, Simon R. Harris, Pierre Lechat, John Lees, Christophe Ginevra, Philippe Glaser, Laurence Ma, Christiane Bouchier, Anthony Underwood, Sophie Jarraud, Timothy G. Harrison, Julian Parkhill, Carmen Buchrieser

**Affiliations:** 1 Wellcome Trust Sanger Institute, Wellcome Genome Campus, Hinxton, CB10 1SA Cambridge, United Kingdom;; 2 Public Health England, NW9 5HT London, United Kingdom;; 3 Institut Pasteur, Biologie des Bactéries Intracellulaires, 75015, Paris, France;; 4 CNRS UMR 3525, 75724, Paris, France;; 5 Hub de Bio-informatique et Biostatistiques, Centre de Bio-informatique, Biostatistique et Biologie Intégrative (C3BI), Institut Pasteur, 75724, Paris, France;; 6 National Reference Centre for Legionella, Hospice Civil de Lyon, International Center for Infection Research, Legionella pathogenesis Team, 69364, Lyon, France;; 7 Institut Pasteur, Evolution et Ecologie de la Resistance aux Antibiotiques, 75724, Paris, France;; 8 Institut Pasteur, Plate-Forme Génomique, 75724, Paris, France

## Abstract

*Legionella pneumophila* is an environmental bacterium and the leading cause of Legionnaires’ disease. Just five sequence types (ST), from more than 2000 currently described, cause nearly half of disease cases in northwest Europe. Here, we report the sequence and analyses of 364 *L. pneumophila* genomes, including 337 from the five disease-associated STs and 27 representative of the species diversity. Phylogenetic analyses revealed that the five STs have independent origins within a highly diverse species. The number of de novo mutations is extremely low with maximum pairwise single-nucleotide polymorphisms (SNPs) ranging from 19 (ST47) to 127 (ST1), which suggests emergences within the last century. Isolates sampled geographically far apart differ by only a few SNPs, demonstrating rapid dissemination. These five STs have been recombining recently, leading to a shared pool of allelic variants potentially contributing to their increased disease propensity. The oldest clone, ST1, has spread globally; between 1940 and 2000, four new clones have emerged in Europe, which show long-distance, rapid dispersal. That a large proportion of clinical cases is caused by recently emerged and internationally dispersed clones, linked by convergent evolution, is surprising for an environmental bacterium traditionally considered to be an opportunistic pathogen. To simultaneously explain recent emergence, rapid spread and increased disease association, we hypothesize that these STs have adapted to new man-made environmental niches, which may be linked by human infection and transmission.

A number of environmental bacteria have emerged to become human pathogens, either through accidental infection or adaptation to the human host. One example is *Legionella*, a bacterium that is ubiquitous in natural aquatic environments but also a contaminant of modern, man-made water systems. *Legionella* can infect humans, mainly through inhalation of contaminated aerosols, and can cause a severe, sometimes fatal, pneumonia known as Legionnaires’ disease (LD) (Fields et al. 2002; Newton et al. 2010). Since the first cases were reported in Philadelphia, Pennsylvania, in 1976, *Legionella* has increasingly been recognized as an important cause of both community- and hospital-acquired pneumonia worldwide (Phin et al. 2014).

*Legionella* are intracellular bacteria whose survival depends on the ability to replicate in eukaryotic cells such as aquatic protozoa (Rowbotham 1980). It is thought that the conservation of signaling pathways and cellular functions from protozoa to higher eukaryotes allows *Legionella* to also infect human alveolar macrophages. However, although humans have traditionally been considered a dead-end host for *Legionella*, one probable case of person-to-person transmission has recently been reported (Correia et al. 2016).

Interestingly, among the 62 species known in the genus *Legionella*, *Legionella pneumophila* is responsible for >90% of known LD cases worldwide (Yu et al. 2002). The prevalence of different *L. pneumophila* subtypes among clinical isolates is also unevenly distributed (Beauté et al. 2013). For example, of the 15 serogroups (sg) described, just one (sg1) is responsible for >80% of culture-confirmed LD cases (Yu et al. 2002; Beauté et al. 2013). Furthermore, data from the sequence-based typing (SBT) scheme (analogous to multilocus sequence typing), that allows a subdivision of *L. pneumophila* into sequence types (ST) based on the sequence of seven genetic loci (http://www.hpa-bioinformatics.org.uk/legionella/legionella_sbt/php/sbt_homepage.php), revealed that a small subset of STs accounts for a disproportionately high number of clinical cases. In particular, five STs (1, 23, 37, 47, and 62) have accounted for nearly half of all epidemiologically unrelated LD cases in northwest Europe reported to the SBT database (Fig. 1A; Supplemental Methods). There is no evidence that the high proportion of isolates found in clinical samples belonging to these five STs is a result of laboratory artifacts such as an increased growth of these STs in culture compared with other STs. Data from 2009 to 2014 obtained by SBT on clinical isolates (*n* = 1762) and nested-PCR-based SBT (NP-SBT) performed directly from respiratory samples of patients (*n* = 99), confirmed that STs 1, 23, 47, and 62 are the major STs in France, representing 41.6% of all STs from SBT and 46.5% of all STs from NP-SBT. The distribution of STs among culture-proven and culture-negative but NP-SBT positive patients appears to be the same for ST1 (9.8%/9.1% for SBT and NP-SBT, respectively); ST23 (18.5%/26.3%), and ST62 (4.8%/8.1%) appear a little more represented in NP-SBT, and ST47 appears a little less represented in NP-SBT (8.5%/3.0%). Furthermore, *L. pneumophila* strains detected by culture (∼65% of LD cases) or PCR (∼20% of LD cases) in another study show a similar distribution of STs (Mentasti et al. 2012).

**Figure 1.**
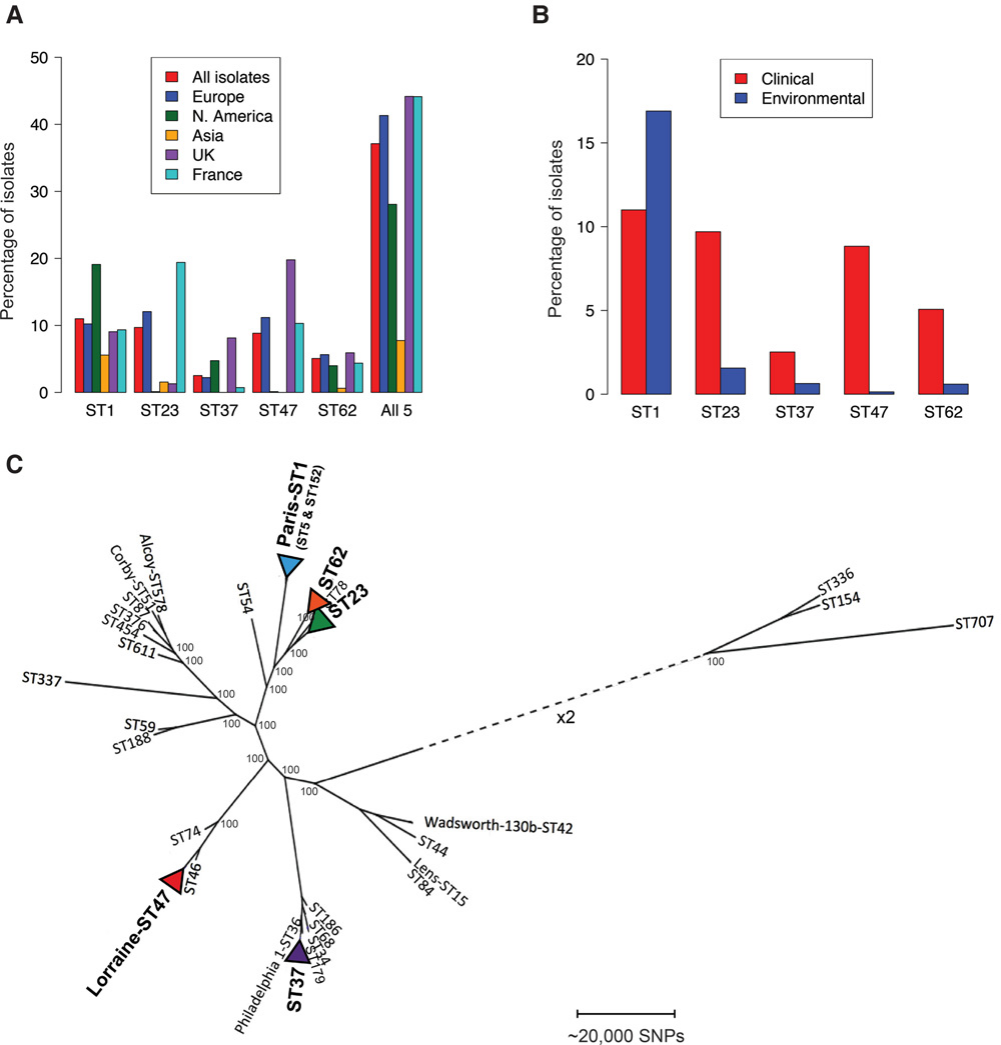
Geographical and environmental distribution of the five major disease-associated STs and their phylogenetic context within the species. (*A*) The percentage of clinical isolates submitted to the ESGLI sequence-based typing (SBT) database for *L. pneumophila* that are sequence types (STs) 1, 23, 37, 47, or 62, or from all five STs combined, are shown. Data are shown from different regions and countries where the numbers of isolates submitted were deemed sufficient for comparison. Data are based on a total of 6116 epidemiologically unrelated clinical isolates (i.e., including only one isolate from clusters/outbreaks) submitted to the database, 4785 of which were detected in Europe, including 541 in the United Kingdom and 2313 in France, as well as 801 in North America and 323 in Asia. (*B*) The percentage of clinical and environmental isolates from each of the five STs from the total number of clinical and environmental isolates submitted to the SBT database. Data are based on a total of 6116 epidemiologically unrelated clinical and 2826 unrelated environmental isolates (i.e., including only one isolate from clusters/outbreaks) submitted to the database. (*C*) Maximum likelihood phylogeny, generated by mapping sequence reads to the Corby reference genome, of representatives from each of the five dominant disease-associated STs together with an additional 27 isolates that are representative of the species diversity. The size of the alignment was 3,576,470 bp. Generally, the five STs are found in separate major clades of the species except for ST23 and ST62 that share a closer phylogenetic relationship. The scale represents the number of SNPs. Bootstrap values, derived from 1000 resamples, are shown for the major nodes of the tree.

Although ST47 has been nearly exclusively isolated in northwest Europe (a small number of cases have been reported from Canada), the other STs have been detected worldwide (Fig. 1A). In particular, ST1 (also known as the “Paris-like strain”) shows a global distribution (Cazalet et al. 2008). The mechanism of global spread is unknown. Interestingly, except for ST1, these disease-associated STs are rarely isolated from either natural or man-made environmental sources (Fig. 1B).

Whole-genome comparisons of *L. pneumophila* isolates have indicated that *L. pneumophila* is a genetically diverse and ancient species (Gomez-Valero et al. 2011; Underwood et al. 2013). Thus, key questions include how and when did these disease-associated STs evolve, and how have they been able to spread globally? Here, we undertook genomic analysis of 337 isolates belonging to five dominant disease-associated STs together with 27 additional *L. pneumophila* isolates representative of the species diversity to investigate their emergence as important human pathogens.

## Results

### The major disease-associated STs have emerged independently

We first analyzed the position of five representative isolates belonging to the major disease-associated STs within a phylogenetic tree containing a total of 32 previously sequenced *L. pneumophila* genomes. These represented the most distantly related STs in the database at the time of their selection (Supplemental Table S1; Underwood et al. 2013). This analysis showed that the five major disease-associated STs are found in separate major clades of the species tree with the exception of ST23 and ST62 that share a closer phylogenetic relationship (Fig. 1C). This suggests that the dominant disease-associated STs have evolved independently from within a genetically diverse species.

To investigate the evolution and diversity of each of the five STs, we separately analyzed 71 ST1 (59 ST1 and 12 “ST1-like isolates”), 37 ST23, 72 ST37, 122 ST47, and 35 ST62 isolates (Supplemental Tables S1, S2) by mapping sequence data to a selected reference genome of the same ST (Supplemental Table S3).

### ST47 isolates show no recombination and are highly clonal

The 122 ST47 isolates were recovered between 1994 and 2013 from the United Kingdom and France, but also include some travel-associated isolates for which the origin is uncertain. In recent years, ST47 has become the most common cause of Legionnaires’ disease in northwest Europe, accounting for more than one-quarter of cases in England and Wales, the Netherlands, France, and Belgium (Harrison et al. 2009; Vekens et al. 2012; Euser et al. 2013; Cassier et al. 2015), yet rarely isolated outside of Europe. ST47 isolates are also extremely rarely isolated from the environment and sources of infection usually remain unknown (Fig. 1B; Edelstein and Metlay 2009; Gomez-Valero et al. 2011).

**Figure 2.**
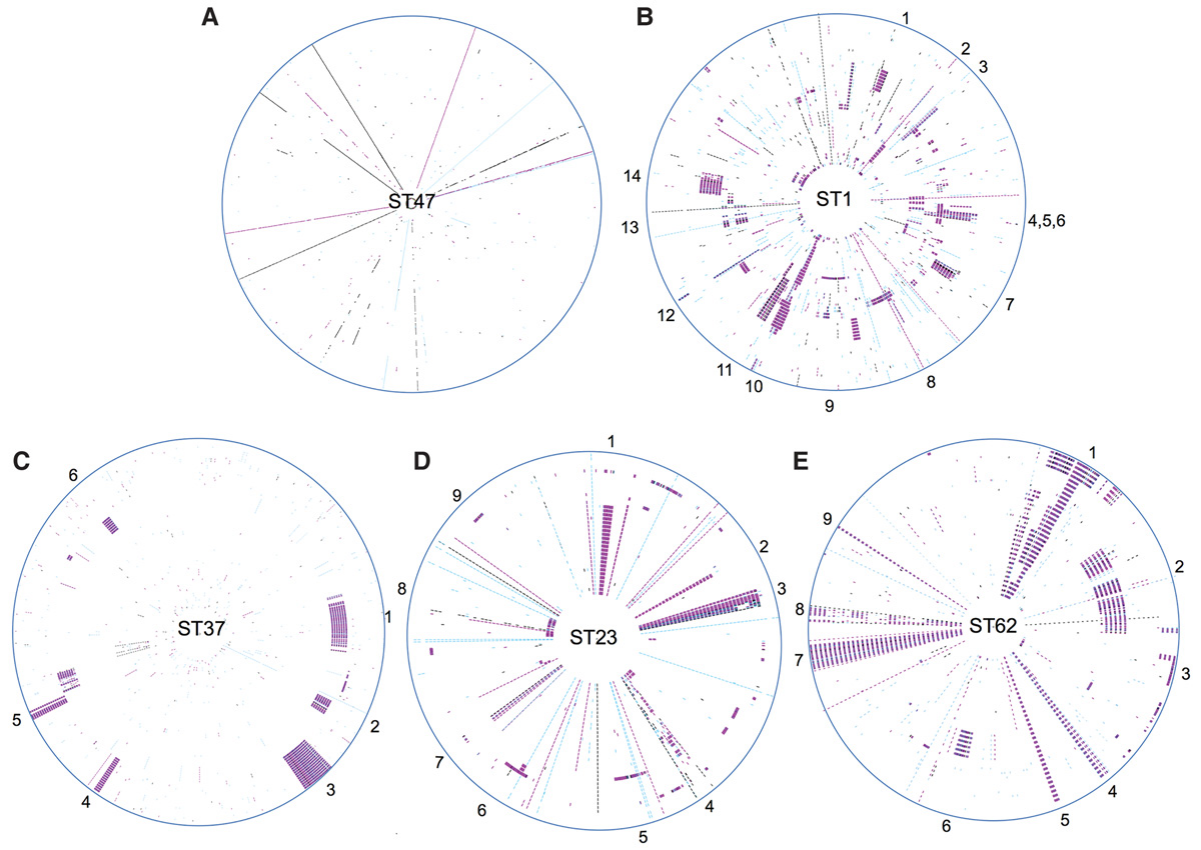
Distribution of SNPs across *L. pneumophila* ST47 (*A*), ST1 (*B*), ST37 (*C*), ST23 (*D*), and ST62 (*E*) lineages. Circular representation of the five major disease-associated STs with each genome shown as a concentric circle. The maps were generated with the SynTView software. SNPs with respect to the reference genome are indicated by short lines in the concentric circle. Recombined regions can be seen as regions with a higher density of SNPs. SNPs are colored according to the type of mutation: (black) intergenic; (pink) synonymous; (blue) nonsynonymous. Around the *outside* circle, selected recombined loci are indicated with numbers, the content of which is provided in Supplemental Tables S5–S8. Access to the interactive SNP server is available at: http://genopole.pasteur.fr/SynTView/flash/Legionella_st/SynWebST1.html, http://genopole.pasteur.fr/SynTView/flash/Legionella_st/SynWebST47.html, http://genopole.pasteur.fr/SynTView/flash/Legionella_st/SynWebST37.html, http://genopole.pasteur.fr/SynTView/flash/Legionella_st/SynWebST23.html, http://genopole.pasteur.fr/SynTView/flash/Legionella_st/SynWebST62.html. The circular representation can be visualised using the “Circular” tab. An alternative linear representation can be displayed using the “Local View” tab (then by selecting the horizontal line between the arrows in the panel on the left hand side, then “snp” and “show/hide snps”).

Surprisingly, the maximum number of SNPs between any pair of the 122 ST47 isolates is just 19. Furthermore, 21 isolates recovered between 2003 and 2012 from distant regions of the United Kingdom have no SNPs at all, and a further 17 isolates are just one SNP different from these 21 isolates. No SNPs are homoplasic, and no recombination events were detected using Gubbins, which uses high SNP density as a marker for recombined regions. This is an expected result given the sparse distribution of SNPs, as visualized using the SynTView program (Fig. 2A; Lechat et al. 2011).

### Recombination is the major driving force of diversity within STs 1, 23, 37, and 62

In contrast, the ST1 isolates were recovered between 1981 and 2011 from 14 countries over four continents (Europe, Asia, North America, and Africa). We included isolates within this data set, here termed “ST1-like isolates,” that are nested within, and thus evolved from, ST1 isolates. We have also sequenced and analyzed the oldest known isolate of *L. pneumophila* (OLDA1/ST1_31), which is an ST1 isolated in 1947, almost 30 years prior to the description of the species. Of the five STs analyzed, the ST1 lineage exhibits the greatest diversity with a maximum of 15,227 SNPs between the two most distant isolates, a sharp contrast to the low number of SNPs observed in the ST47 lineage (Supplemental Table S3).

Isolates belonging to STs 23, 37, and 62 are commonly isolated across Europe and occasionally elsewhere (Fig. 1A); thus, the extent of their distributions appears to be between those of ST1 and ST47. Like ST47 isolates, they are only rarely isolated from commonly expected environmental sources of *Legionella* (Fig. 1B). The ST23 and ST37 isolates analyzed in this study were recovered between 1987 and 2012, and the ST62 isolates were recovered between 1994 and 2012. The SNP analyses showed that the maximum pairwise SNP differences between isolates are 12,964, 13,776, and 12,842 within the ST23, ST37, and ST62 lineages, respectively (Supplemental Table S3), slightly lower than that observed in the ST1 lineage.

However, when we analyzed the origin of these nucleotide variants using Gubbins, we found that 96.3%–99.0% of SNPs in STs 1, 23, 37, and 62 had been acquired by recombination (Table 1). There was a high level of concordance between these results and those obtained using an alternative recombination detection software, BRATNextGen (Marttinen et al. 2012), which uses a hidden Markov model (HMM) to detect SNP patterns in an isolate that are more similar to those from another phylogenetic clade than the isolate's own clade. Overall, >90% of SNPs identified as recombined by Gubbins were confirmed by BRATNextGen to be within horizontally exchanged regions. The importance of recombination within these STs becomes very apparent when the SNP distributions are visualized using SynTView (Fig. 2B–E). The locations and content of the recombined regions are provided in Supplemental Tables S4–S8, and the distribution of recombination fragment lengths is shown in Supplemental Figure S1. Further details on their composition and predicted origin are also given in the Supplemental Results.

**Table 1.**
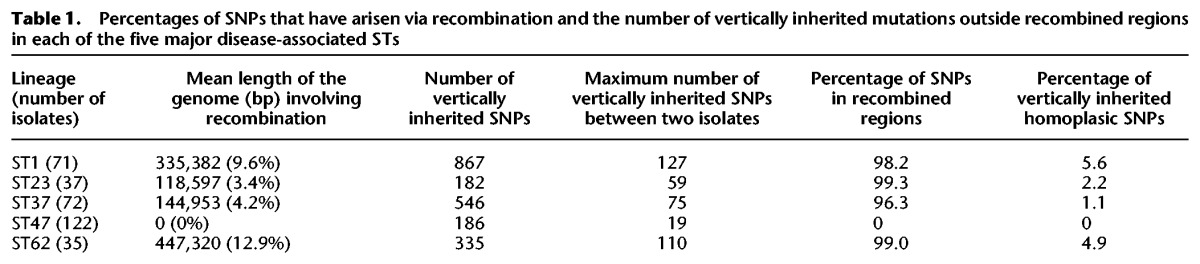
Percentages of SNPs that have arisen via recombination and the number of vertically inherited mutations outside recombined regions in each of the five major disease-associated STs

After removal of recombined regions (representing a mean of 3.4% [ST23] to 12.9% [ST62] of the 3.2-Mb genomes) from the analysis of STs 1, 23, 37, and 62 to leave only those SNPs generated by point mutation, the maximum pairwise SNP differences ranged from just 59 (ST23) to 127 (ST1), similar to the 19 SNPs observed in the ST47 lineage (Table 1). There might be additional de novo mutations that have occurred within the recombined regions and removed from this analysis; however, these are unlikely to constitute more than another 12.9%, in proportion with the length of genome removed. Thus, all five STs are characterized by a very low number of de novo mutations, in sharp contrast to the high species diversity.

### The major disease-associated clones emerged very recently

**Figure 3.**
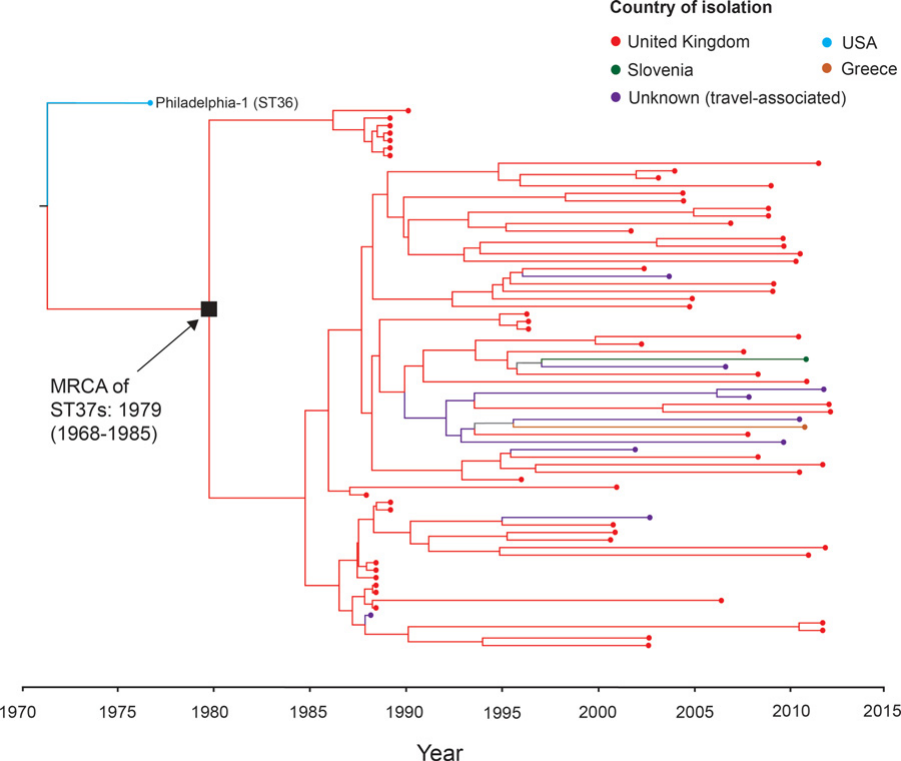
Maximum clade credibility tree of the ST37 lineage showing the estimated age of the MRCA. A time-dependent phylogenetic reconstruction of the ST37 lineage, inferred by Bayesian inference using BEAST, is shown. The Philadelphia isolate (a single locus variant of ST37) was also included in the analysis as an out-group. The node representing the MRCA of the ST37 lineage is labeled with the median estimate for the inferred date and the 95% highest posterior probability (HPD) intervals. Isolates are colored according to the country of isolation, and branches are similarly colored to indicate the origin of descendant nodes.

The small number of de novo mutations within each of the five major disease-associated STs suggests that these are very recently emerged lineages. To estimate their emergence date, we attempted to date the most recent common ancestor (MRCA) of each lineage using linear regression of root-to-tip distances against time, as well as with a Bayesian coalescent model as implemented in the BEAST software (Drummond et al. 2012). Only the ST37 lineage showed some temporal signal in terms of SNP accumulation using Path-O-Gen (Supplemental Fig. S2). Using a relaxed molecular clock model in BEAST, we estimated that the ST37 clone emerged in about 1979 (95% highest posterior density [HPD] intervals: 1968–1985) (Fig. 3), which is 3 yr prior to the earliest ST37 isolate recorded in the SBT database. The evolutionary rate estimated by BEAST is 2.07 × 10^−7^ substitutions per site per year (95% HPD interval: 1.69 × 10^−7^ to 2.44 × 10^−7^), very similar to that previously calculated for the ST578 lineage (1.39 × 10^−7^) (Table 2; Sánchez-Busó et al. 2014). We further used the estimated evolutionary rates of the ST37 and ST578 lineages to provide approximations of the length of time it would have taken for the observed diversity in STs 1, 23, 47, and 62 to arise. This analysis suggested emergence dates of 1851/1899 for ST1, 1972/1983 for ST23, 1943/1964 for ST62, and 1998/2002 for ST47, with the two dates provided corresponding to the application of the ST578 and ST37 mean evolutionary rates, respectively. Further details on all dating analyses are provided in the Supplemental Methods and Results.

**Table 2.**
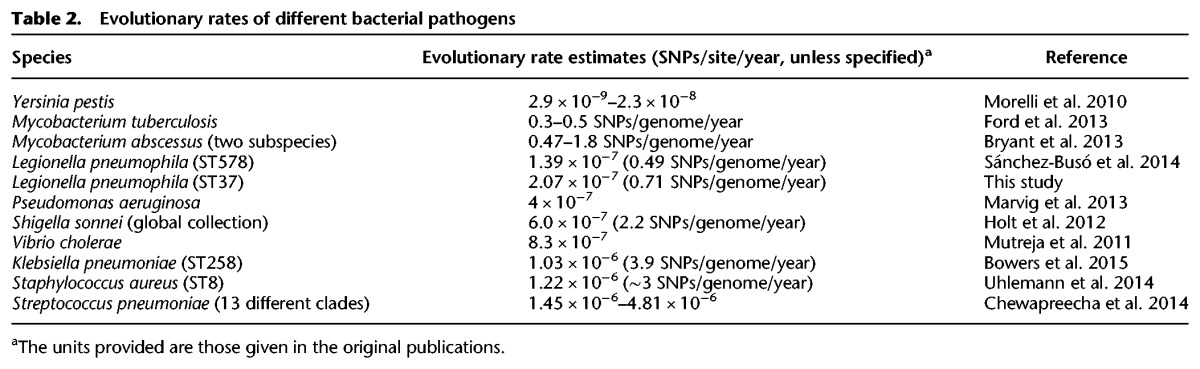
Evolutionary rates of different bacterial pathogens

### The disease-associated clones have spread rapidly and internationally

**Figure 4.**
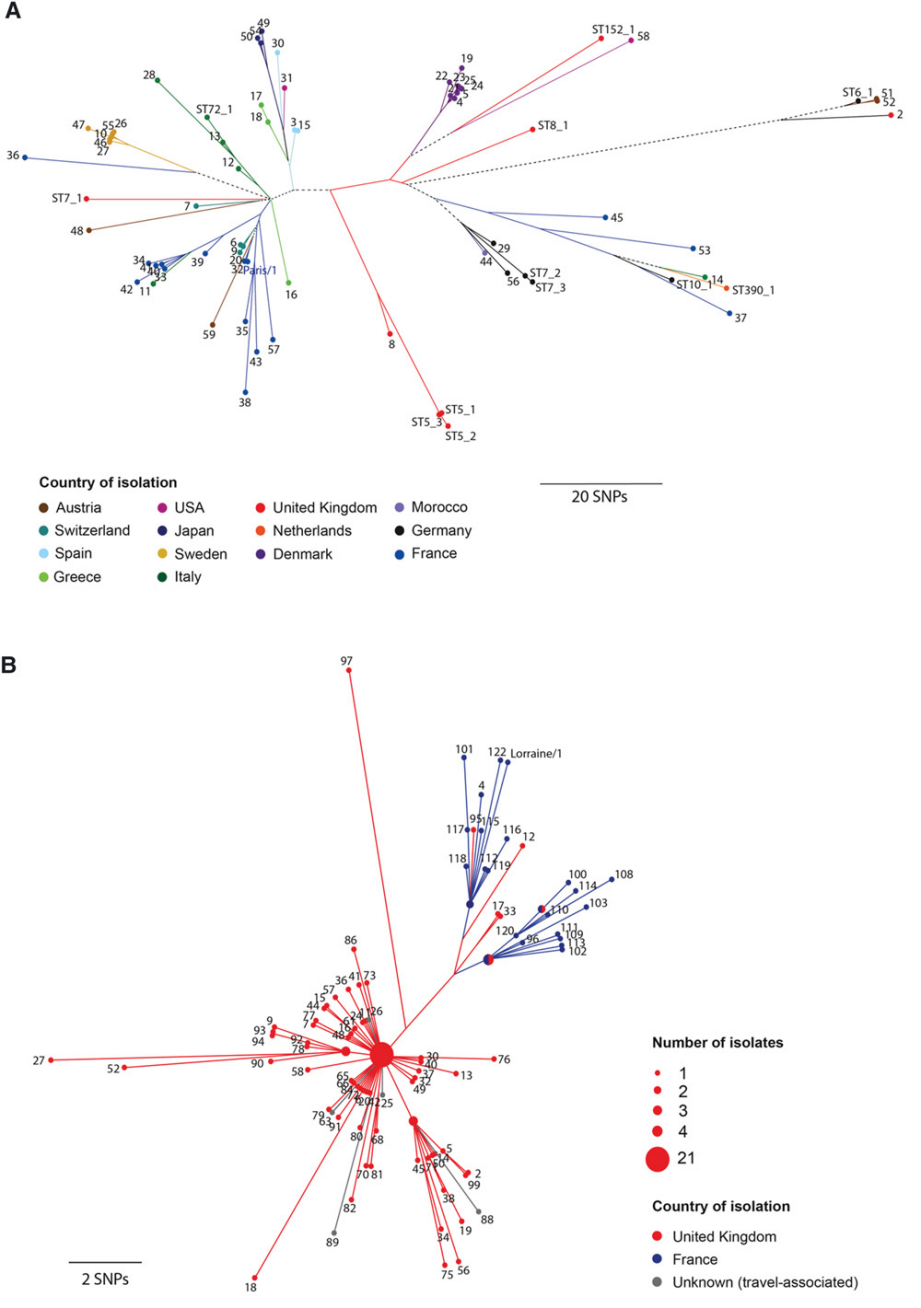
Maximum likelihood trees of 71 ST1 and 122 ST47 isolates. (*A*) A phylogeny of ST1 isolates, constructed using 867 SNP differences remaining after recombined regions were removed. (*B*) A phylogeny of ST47 isolates, constructed using 186 SNP differences. Isolates are colored according to the country of isolation, and branches are similarly colored to indicate the origin of descendant nodes. A black dotted line is used where there are descendant nodes from multiple countries. The scales indicate the number of SNPs that have occurred for a given branch length.

Phylogenetic analyses of the five STs show that isolates from the same country do not always cluster together, whereas isolates from distant geographical regions frequently cluster very closely (Figs. 3, 4; Supplemental Figs. S3–S7). This is most apparent in the globally dispersed ST1 lineage, but true in all lineages, including the more geographically restricted ST47 lineage, whereby isolates from the United Kingdom are nested within a cluster of predominantly French isolates (Fig. 4B). These results demonstrate the occurrence of multiple, recent, long-distance spreading events. It is also notable that the geographical distribution of these STs correlates with their predicted ages. For example, ST1 is estimated to be the oldest lineage and is distributed globally, whereas ST47 is the youngest predicted lineage and is mostly restricted to northwest Europe.

### The disease-associated clones show evidence of convergent evolution via recent recombination

**Figure 5.**
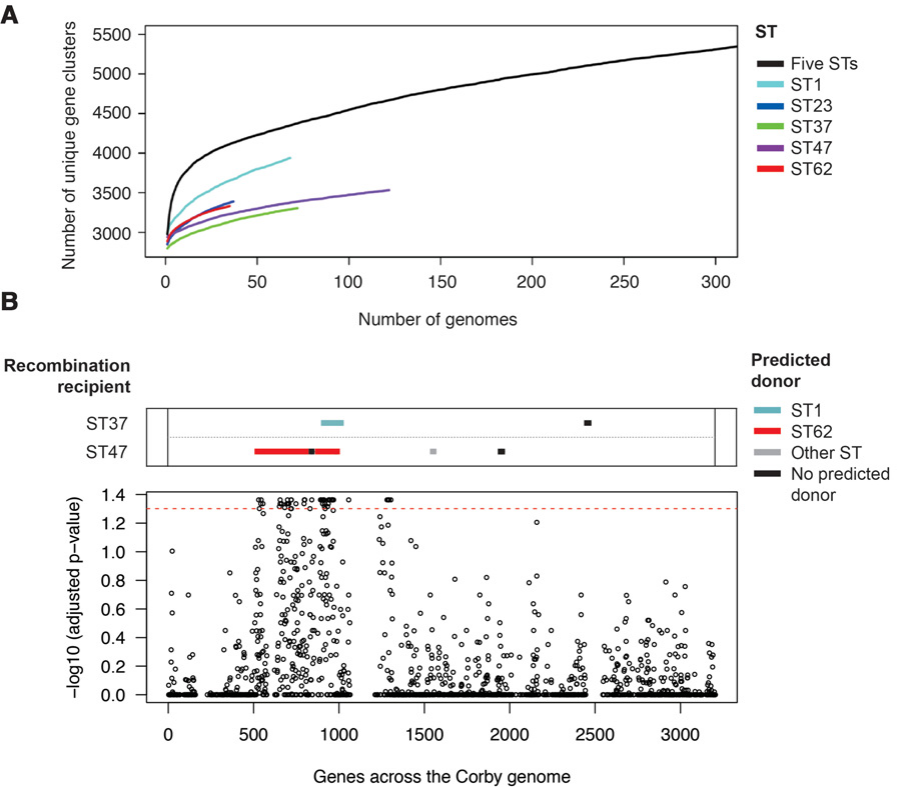
Gene content analysis and the nucleotide diversity of core genes within the five disease-associated STs. (*A*) Rarefaction curves applied to the strains of *L. pneumophila* ST1 (71 isolates), ST23 (37 isolates), ST37 (72 isolates), ST47 (122 isolates), ST62 (35 isolates), and all five STs together (337 isolates), showing that differences in gene content exist among the five STs, but that the number of novel genes in the overall pan-genome is beginning to plateau. (*B*) Log-transformed *P*-values derived from testing whether the five disease-associated STs have lower than expected nucleotide diversity values in individual core genes given their nucleotide diversity across all 1888 core genes, and with respect to the gene conservation across the species (excluding isolates from the distant subspecies, strains ST5 and ST152, which are nested within ST1, and strains ST36 [Philadelphia], ST42, and ST578 [Alcoy], which are also disease-associated strains). The core genes are ordered as in the Corby genome. Any noncore genes (genes in <100% isolates) are omitted. The horizontal dotted red line indicates the significance threshold when the Benjamini-Hochberg method is applied to correct for multiple testing. The box at the *top* shows the location and predicted origins of recombined regions that were detected on the branches leading to the ST37 and ST47 lineages. Recombined regions that were found in the ST37 and ST47 accessory genomes are not shown.

Next, we investigated whether specific signatures of convergent evolution exist between the five STs that could explain possible adaptation to a common niche or increased propensity to cause disease compared with other STs. Although many of the specific isolates from the other STs were from LD patients, the STs to which they belong are far less associated with disease than isolates belonging to STs 1, 23, 37, 47, and 62. Analysis of the gene content using de novo assemblies of all isolates did not identify any genes specifically present in the five STs but absent from the other genomes from our collection. Analyses of the pan-genome of each of the five STs individually showed that the pan-genome content of each ST is beginning to plateau, suggesting that this gene analysis is representative of each ST (Fig. 5A). For example, the 306 known substrates of the Dot/Icm secretion system, key virulence factors of *L. pneumophila*, were all highly conserved across the five STs (Supplemental Table S9). Further details of this analysis are provided in the Supplemental Results. This finding led us to focus our attention on core genes (i.e., genes that are shared among all isolates).

Analysis of all core genes using CodeML identified none that had been subjected to positive selection on more than one of the five branches in the species tree leading to each of the disease-associated STs, a result which could have indicated common adaptation to a particular niche. Further details are provided in the Supplemental Methods and Results. However, we did identify seven SNPs that are convergent on four of these branches, and 38 on three branches (Supplemental Table S10). One of the SNPs that occurred on four of the five branches (leading to STs 1, 37, 47, and 62) causes an amino acid change and was also found on two other branches of the species tree. This SNP is in *lpp0942/lpg0879*, a gene that encodes a diguanylate kinase with a GGDEF domain, which is strongly induced in the virulent, transmissive phase of infection and belongs to the transmissive phase core genes (Brüggemann et al. 2006; Weissenmayer et al. 2011). However, further studies are required to test if this SNP, or any of the others, affect disease propensity.

Finally, we searched for core genes with a higher than expected nucleotide similarity in the five STs with respect to the rest of the species. This approach bypasses a limitation of the previous approaches by taking into account all evolution that has occurred en route to the formation of the five disease-associated STs, rather than searching for evidence of convergent evolution on individual, sometimes short, branches leading to each lineage. To perform this analysis, we first identified core genes present in all 32 STs with the exclusion of ST154, ST336, and ST707 that are distantly related. Using one representative isolate from each of STs 1, 23, 37, 47, and 62, we calculated the nucleotide diversity (π) value, as first described by Nei and Li (1979), between the five isolates for each of the 1888 core genes. Interestingly, a ∼700-kb region of the genome was identified that contains several genes with a very low nucleotide diversity (π) value in the five STs as compared to the rest of the genome. Because this region could simply be more conserved across the species in general, we next tested whether each gene was significantly more similar between the five disease-associated STs than expected given its degree of conservation across the entire species. We also took into account the nucleotide diversity (π) values observed in the five STs across the whole genome, thus accounting for phylogenetic distance. Further details of these methods are provided in the Supplemental Methods. After correcting for multiple testing, we found that nucleotide diversity (π) in the five disease-associated STs was statistically lower than expected in 64 genes (*P* < 0.05) (Supplemental Table S11), which are all located in the aforementioned region of 725.1 kb (*lpp0536*/*LPC_2873* to *lpp1176*/*LPC_0640*) (Fig. 5B; Supplemental Fig. S8). Maximum likelihood trees of selected individual gene alignments confirmed that the genes from the five STs cluster together in contrast to their positions within the whole-genome phylogeny (Supplemental Fig. S9). Among the 64 genes, some have been shown to play a role in intracellular infection such as the genes from the cytochrome *c* maturation (*ccm*) locus (Viswanathan et al. 2002; Naylor and Cianciotto 2004), PilR (an important regulator for pilin and flagella synthesis), the phagosomal transporter family Pht (Sauer et al. 2005), or the enhanced entry protein EnhA that was shown to be important for entry in phagocytic cells (Cirillo et al. 2000) and during persistence in water environments (Li et al. 2015).

The detection of shared gene variants within the five STs led us to hypothesize that they have arisen via recombination events prior to the emergence of these major disease-associated STs. Using Gubbins, we were able to detect recombination events on the branches leading to STs 37 and 47, because they contain relatively few SNPs and allow the SNP-dense recombined regions to be detectable above the background level. Indeed, we detected a number of imported recombined regions on both branches that shared 100%, or almost 100%, similarity with other major disease-associated STs (Fig. 5B; Supplemental Table S12) and that lie within the previously detected region of highly similar genes. Of particular note is the large amount of sequence (396,135 bp) imported from the ST62 lineage to ST47, which makes up 11.4% of the ST47 chromosome (Supplemental Fig. S8).

## Discussion

Genomic and phylogenetic analysis of 364 *L. pneumophila* isolates revealed that five major disease-associated STs emerged independently from different genomic backgrounds. In contrast to the high species diversity, they show remarkably little diversity (excluding recombined regions), suggesting recent clonal origins. Further support for the recent emergence of these STs is provided by BEAST analysis of the ST37 lineage, which predicts the most likely emergence date to be between 1968 and 1985. When the estimated evolutionary rate of the ST37 lineage, and that of the previously published Alcoy lineage (Sánchez-Busó et al. 2014), is applied to the four remaining lineages, emergences in the last century are also predicted. Although these observations might be expected for a human-adapted pathogen, the results are surprising for an environmental bacterium that is traditionally thought to “accidentally” infect humans when given the opportunity. The results suggest that these *L. pneumophila* clones have adapted to new niches that presumably are related to modern, man-made water systems, from which the majority of infections are acquired. However, because most of these disease-associated STs are not more frequently detected in commonly expected environmental sources than other STs, and indeed some are rarely found, it is possible that they are also more efficient at infecting humans.

Because the five disease-associated STs have emerged independently from within the species, we explored whether there are signs of convergent evolution that could explain their common adaptation to specific niches and increased propensity to cause human disease. Indeed, we identified many genes with particular allelic variants in the five STs that are rarely seen in other STs, and which have arisen at least partially via recombination events. This finding, together with observations from this study and others (Gomez-Valero et al. 2011; Sánchez-Busó et al. 2014) that recombination accounts for almost all the observed diversity in some STs, confirms the importance of this process for *L. pneumophila* evolution and the emergence of disease-associated lineages.

In contrast to all other STs studied, no recombination was detected within the ST47 lineage. Although this lack of observed recombination events may simply reflect its very recent emergence leaving no time for recombination to occur, it could also suggest that ST47 inhabits a specific environmental niche where no opportunity for recombination exists. ST47 may also have lost the ability to recombine with other *L. pneumophila* strains, or have lost natural competence. However, we have been able to construct a streptomycin-resistant ST47 isolate by natural competence, and thus the latter possibility can be ruled out.

The evolutionary rate, as estimated by BEAST, is very low with on average 2.07 × 10^−7^ SNPs/site/year (0.71 SNPs/genome/year) for the ST37 lineage (Table 2). This is similar to the rate of 0.49 SNPs/genome/year estimated for the Spanish ST578 lineage (Sánchez-Busó et al. 2014). Further support for a low evolutionary rate is provided by the 21 identical ST47 isolates recovered between 2003 and 2012 as well as the existence of only 20 vertically inherited SNPs between the OLDA1 isolate from 1947 and another ST1 isolate from 1995. The evolutionary rate is comparable to that described for *Mycobacterium tuberculosis*, a notoriously slow-evolving pathogen (Ford et al. 2013). Together with the absence of a strict molecular clock, the low evolutionary rate suggests that *Legionella* may undergo periods of dormancy, either within amoebas, biofilm, or during its free-living phase, and perhaps also due to the water temperature in temperate climates being <15°C for most of the year.

Phylogenetic analyses of each of these disease-associated STs showed that isolates from different countries, and even different continents, differ by just a few SNPs (Figs. 3, 4; Supplemental Figs. S3–S7). This demonstrates the occurrence of long-distance spread, which must have occurred relatively rapidly given the recent emergence of these STs. Possible spreading mechanisms include wind transport, natural water currents, or human-related activities such as the movement of contaminated vehicles, ships, or other objects harboring water. The latter possibility may also explain the recent emergence of these clones, because they may have adapted to the new environments. However, it would be very surprising that those few STs that are highly associated with human disease have also spread widely and rapidly, if these phenomena were unlinked.

The observations gained from our evolutionary, phylogenetic, and comparative genome analyses lead us to hypothesize that *L. pneumophila*–infected humans may indeed contribute to the spread of these highly disease-causing strains by linking modern man-made water systems through human transmission. *L. pneumophila* has been isolated from human feces (Rowbotham 1998) and is regularly isolated from the sputa of Legionnaires’ disease patients, suggesting that human infection may not actually be a dead end. Further support for this has also come from a recently reported case of probable person-to-person transmission of Legionnaires’ disease (Correia et al. 2016). Scenarios involving human-to-human transmission and/or human-to-environment transmission could simultaneously explain why these specific strains have emerged recently, spread widely, and are primarily associated with human infection. Adaptation to man-made water systems, when coupled with human infection and transmission at least partially via humans, would select strains most fit for human infection. These would then be more frequently transmitted to other man-made water systems. Humans as vectors would also link similar sites, enhancing the ability of these clones to adapt to this niche and promoting long-distance transmission. Such a scenario would effectively create a new evolutionary niche and allow expansion and further adaptation of these clones. The finding that this has happened independently to multiple strains suggests that it is the new niche that has driven the establishment and expansion of these strains, rather than the attributes of a specific strain.

The discovery of several independent disease-associated clones that are recently emerged has important implications for the understanding of Legionnaires’ disease. In particular, our data support the idea that the majority of clinical cases do not arise due to infection by any *L. pneumophila* strain that happens to be present in a source, but rather are caused by selected clones that may have adapted to a specific niche. Identifying the environmental niche and mechanism of spread of these clones should become a priority if we are to reduce human exposure to *L. pneumophila* and alleviate the disease burden. We also believe that our hypothesis that specific *L. pneumophila* clones may have shifted from being accidental to more human-adapted pathogens is worthy of further investigation.

## Methods

### Bacterial isolates and whole-genome sequencing

Of the 364 *L. pneumophila* isolates used in this study, 35 have been previously sequenced, whereas 329 are newly sequenced (Supplemental Tables S1, S2). All newly sequenced isolates are from the culture collections at Public Health England (PHE), United Kingdom, and the *Legionella* National reference center, Lyon (LRC), France. Details of these isolates and their sequencing are provided in the Supplemental Methods.

### Mapping of sequence reads and phylogenetic analysis

Sequence reads were mapped to a reference genome using SMALT v0.7.4, and SNPs were identified using a standard approach (Harris et al. 2010). Further details are provided in the Supplemental Methods. After removing recombined regions, as defined by Gubbins (Croucher et al. 2014) (except for the species representatives, as the large amount of diversity renders recombination detection very difficult), maximum likelihood trees were constructed based on variable positions using RAxML v7.0.4 (Stamatakis 2006). A general time reversible (GTR) model with gamma correction for among-site rate variation and 1000 bootstrap replicates were used. SNPs were reconstructed onto the individual phylogenies using accelerated transformation parsimony, meaning that SNPs are inferred to have occurred as early as possible.

### Time-dependent phylogenetic reconstruction

Linear regression analysis of the root-to-tip distances against sampling time was performed using Path-O-Gen. Time-dependent phylogenetic reconstructions and calculations of evolutionary rates were undertaken using BEAST v1.7 (Drummond et al. 2012). The evolutionary rates of ST37 (this study) and ST578 (Sánchez-Busó et al. 2014) were used to infer the approximate length of time it would have taken the diversity in the STs 1, 23, 47, and 62 lineages to arise. Further details are provided in the Supplemental Methods.

### Gene content analysis

De novo assemblies were generated using an in-house Sanger Institute pipeline that uses Velvet (Zerbino and Birney 2008), SSPACE (Boetzer et al. 2011), and GapFiller (Boetzer and Pirovano 2012). Prodigal software was used to predict genes in the assemblies, which were then clustered into orthologous groups using BLAST+ (Blastp) and the micropan R package (Snipen and Liland 2015). Custom Python scripts were used to identify “accessory” genes present in STs 1, 23, 37, 47, and 62, but not in other STs.

### Identification of core genes under positive selection in the five STs

The branch-site model in CodeML was used to test whether any core genes (i.e., genes found in every isolate in the collection) had been subjected to positive selection on the branches leading to each of the five disease-associated STs. Further details are provided in the Supplemental Methods.

### Identification of core genes with high nucleotide similarity in the five STs

The core genome of the ST representatives, with the exclusion of the distantly related STs (ST336, ST154, and ST707), was defined using Roary (Page et al. 2015). For each core gene, a nucleotide alignment was generated using one representative from each of the five STs—Paris/ST1 (Cazalet et al. 2004), EUL00011/ST23_3, EUL00132/ST37_69, Lorraine/ST47 (Gomez-Valero et al. 2011), H043540106/ST62_2—and excluding all other ST representatives. The nucleotide diversity (π) value, a measurement first described by Nei and Li (1979), was calculated for each of these alignments using the R package, “pegas,” and custom Python scripts. To test whether each core gene possessed significantly higher nucleotide similarity (or lower diversity) in the five major disease-associated STs than would be expected, these values were compared to those derived from testing all possible combinations of five STs from the set of species representatives, after adjusting for phylogenetic distance. Further details are provided in Supplemental Methods.

### Prediction of recombination donors

Each predicted recombined region was used as a query sequence in BLASTn to identify matches among the de novo assemblies of all 364 isolates used in this study. BLAST hits with a *P*-value of <1 × 10^−5^ and >75% of the length of the recombined region were recorded.

## Data access

Raw sequence reads from this study have been submitted to the European Nucleotide Archive (ENA; http://www.ebi.ac.uk/ena) under accession numbers ERP002503, ERP003631, and ERP010118. The assembled genomes used as references for STs 23 (EUL00011/ST23_3), 37 (EUL00132/ST37_69), and 62 (H043540106/ST62_2) have also been submitted to ENA under accession numbers FJBI01000001–FJBI01000031, FJFB01000001–FJFB01000024, and FJLN01000001–FJLN01000039, respectively. The scripts used for the diversity analyses are in the Supplemental Material and are available at https://github.com/sophiadavid1/diversity_analysis.

## Supplementary Material

Supplemental Material
