# Supplementary material for: Multiple major disease-associated clones of Legionella pneumophila have emerged recently and independently
Source: Genome Res. 2016 Nov;26(11):1555–64. doi: 10.1101/gr.209536.116 (PMC5088597; doi:10.1101/gr.209536.116)
Supplement: Supplemental Material [file supp_26_11_1555__index.html]

Multiple major disease-associated clones of Legionella pneumophila have emerged recently and independently — Multiple major disease-associated clones of Legionella pneumophila have emerged recently and independently — Supplemental Material 

# Multiple major disease-associated clones of *Legionella pneumophila* have emerged recently and independently

## Supplemental Material

- Supplemental\_Fig\_S1.pdf
- Supplemental\_Fig\_S2.pdf
- Supplemental\_Fig\_S3.pdf
- Supplemental\_Fig\_S4.pdf
- Supplemental\_Fig\_S5.tif
- Supplemental\_Fig\_S6.tif
- Supplemental\_Fig\_S7.tif
- Supplemental\_Fig\_S8.pdf
- Supplemental\_Fig\_S9.tif
- Supplemental\_Material\_Contents.docx
- Supplemental\_Material\_References.docx
- Supplemental\_Methods.docx
- Supplemental\_Results.docx
- Supplemental\_Table\_S1.docx
- Supplemental\_Table\_S2.docx
- Supplemental\_Table\_S3.docx
- Supplemental\_Table\_S4.docx
- Supplemental\_Table\_S5.xlsx
- Supplemental\_Table\_S6.xlsx
- Supplemental\_Table\_S7.xlsx
- Supplemental\_Table\_S8.xlsx
- Supplemental\_Table\_S9.docx
- Supplemental\_Table\_S10.docx
- Supplemental\_Table\_S11.docx
- Supplemental\_Table\_S12.docx
- Supplemental\_nucleotide\_diversity\_analysis-master.zip
