# Supplementary figures and images for: Multiple major disease-associated clones of Legionella pneumophila have emerged recently and independently
Source: Genome Res. 2016 Nov;26(11):1555–64. doi: 10.1101/gr.209536.116 (PMC5088597; doi:10.1101/gr.209536.116)

A (ST1)

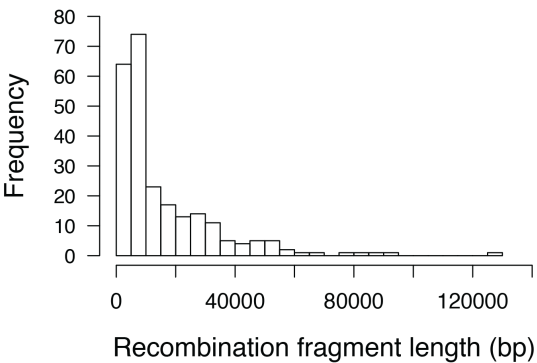

B (ST23)

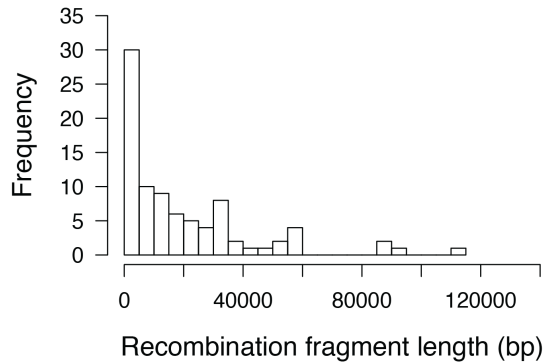

C (ST37)

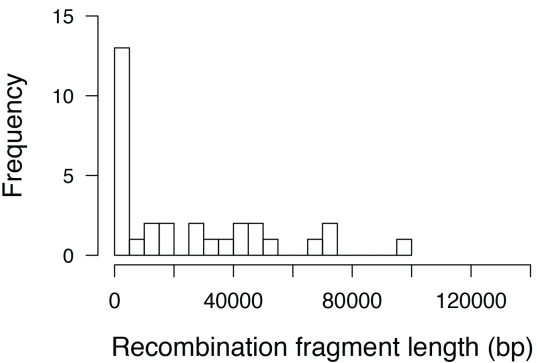

D (ST62)

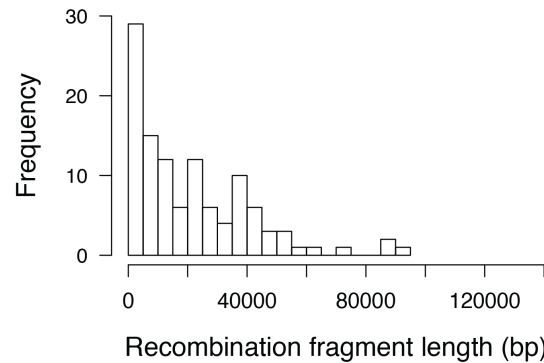

Supplement: Supplemental Material [file supp_gr.209536.116_Supplemental_Fig_S1.pdf]

A (ST1)

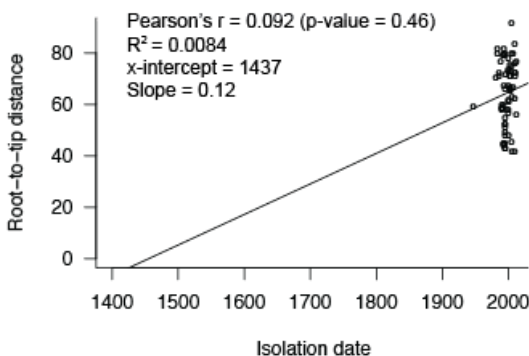

B (ST23)

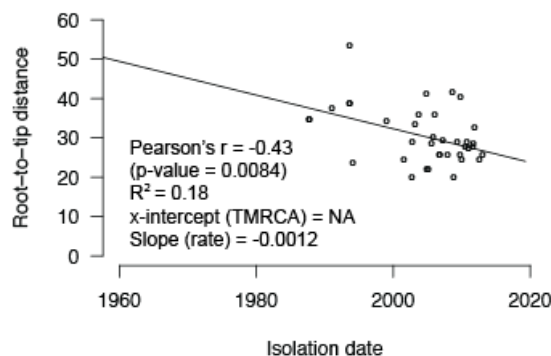

C (ST37)

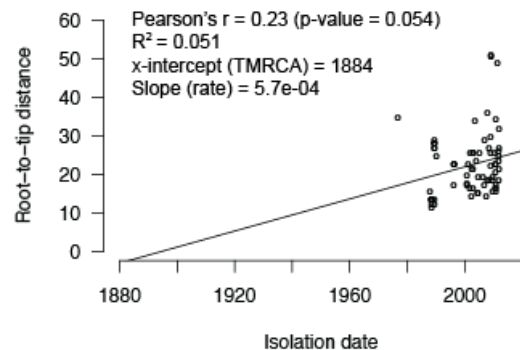

D (ST47)

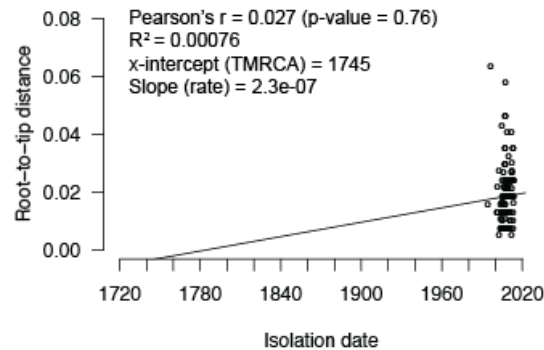

E (ST62)

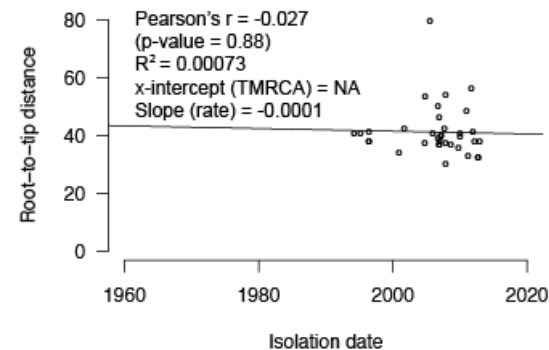

Supplement: Supplemental Material [file supp_gr.209536.116_Supplemental_Fig_S2.pdf]

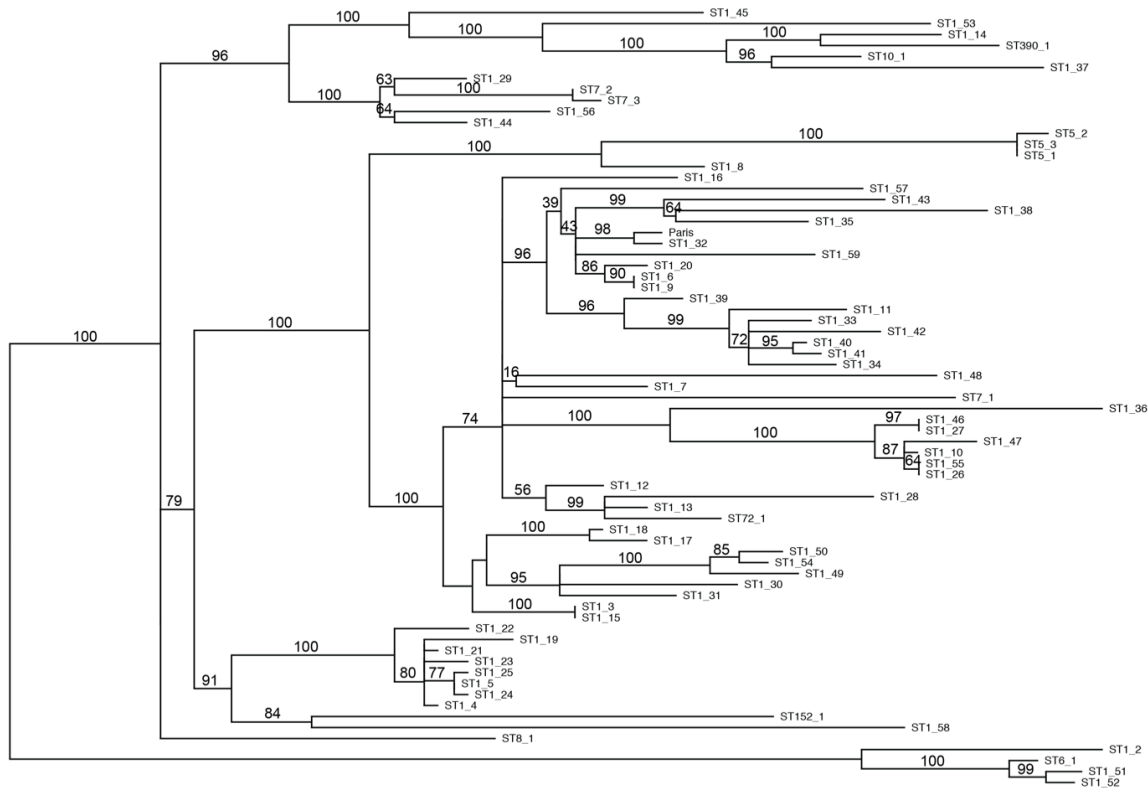

Supplement: Supplemental Material [file supp_gr.209536.116_Supplemental_Fig_S3.pdf]

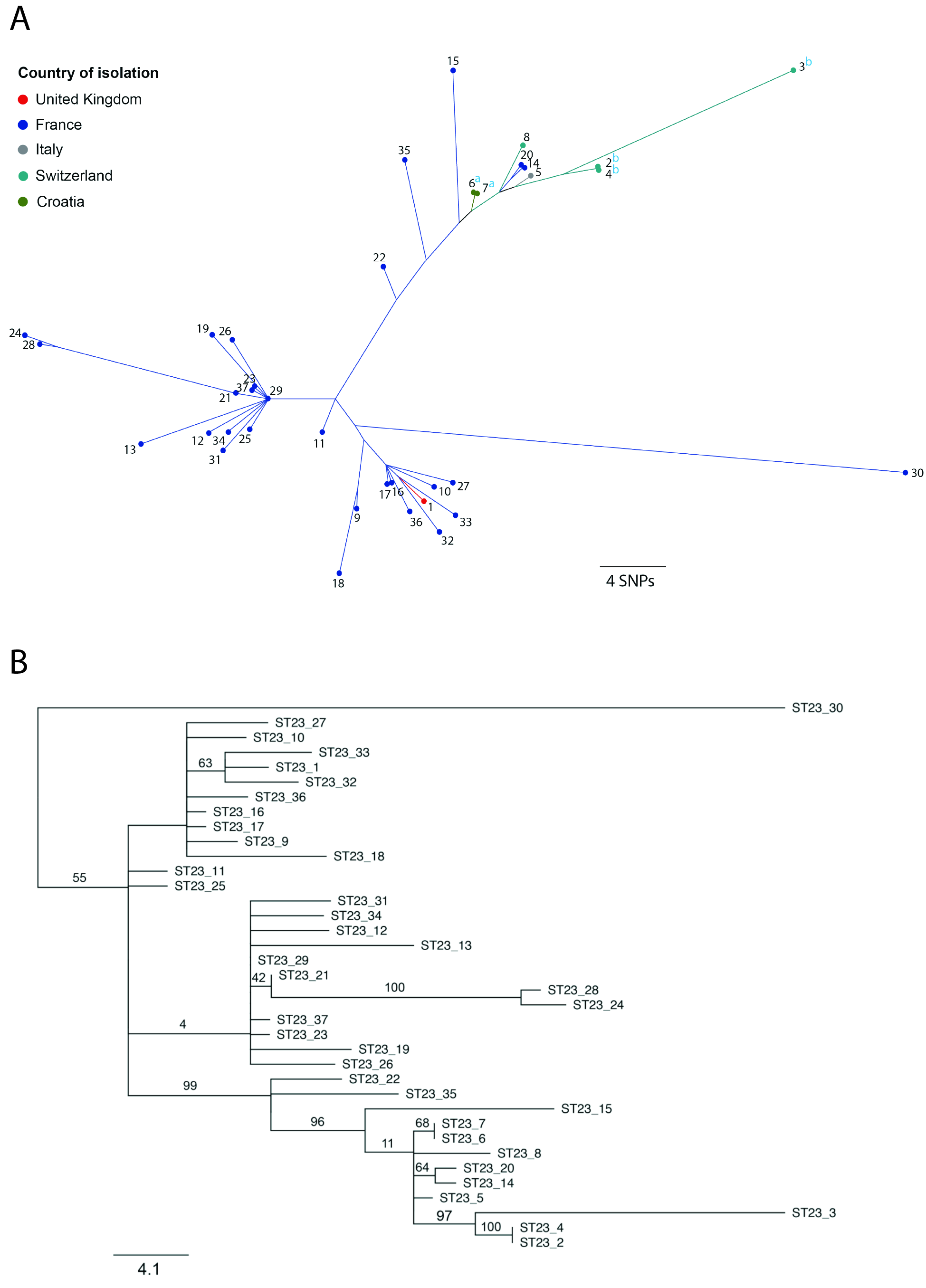

Supplement: Supplemental Material [file supp_gr.209536.116_Supplemental_Fig_S5.tif]

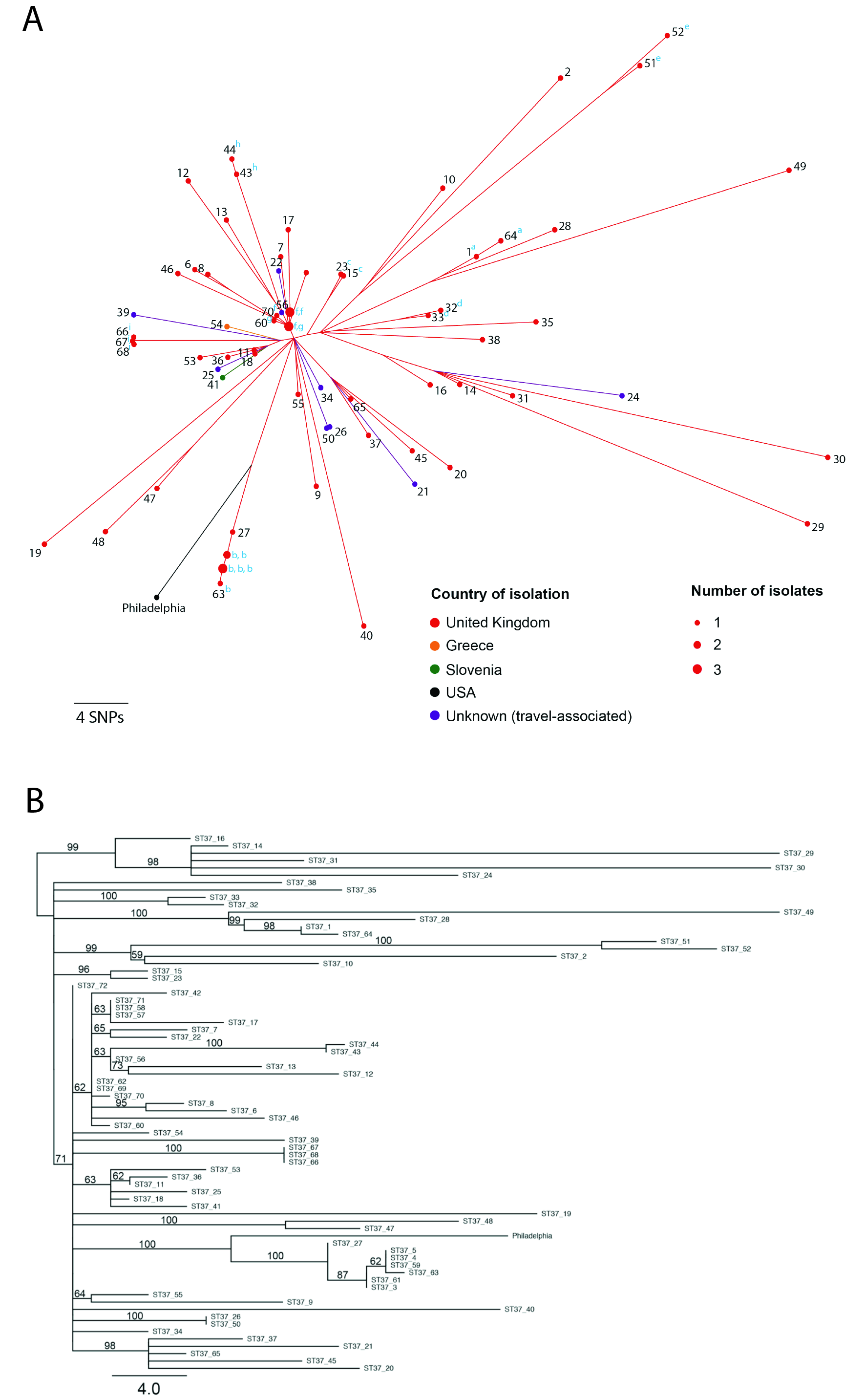

Supplement: Supplemental Material [file supp_gr.209536.116_Supplemental_Fig_S6.tif]

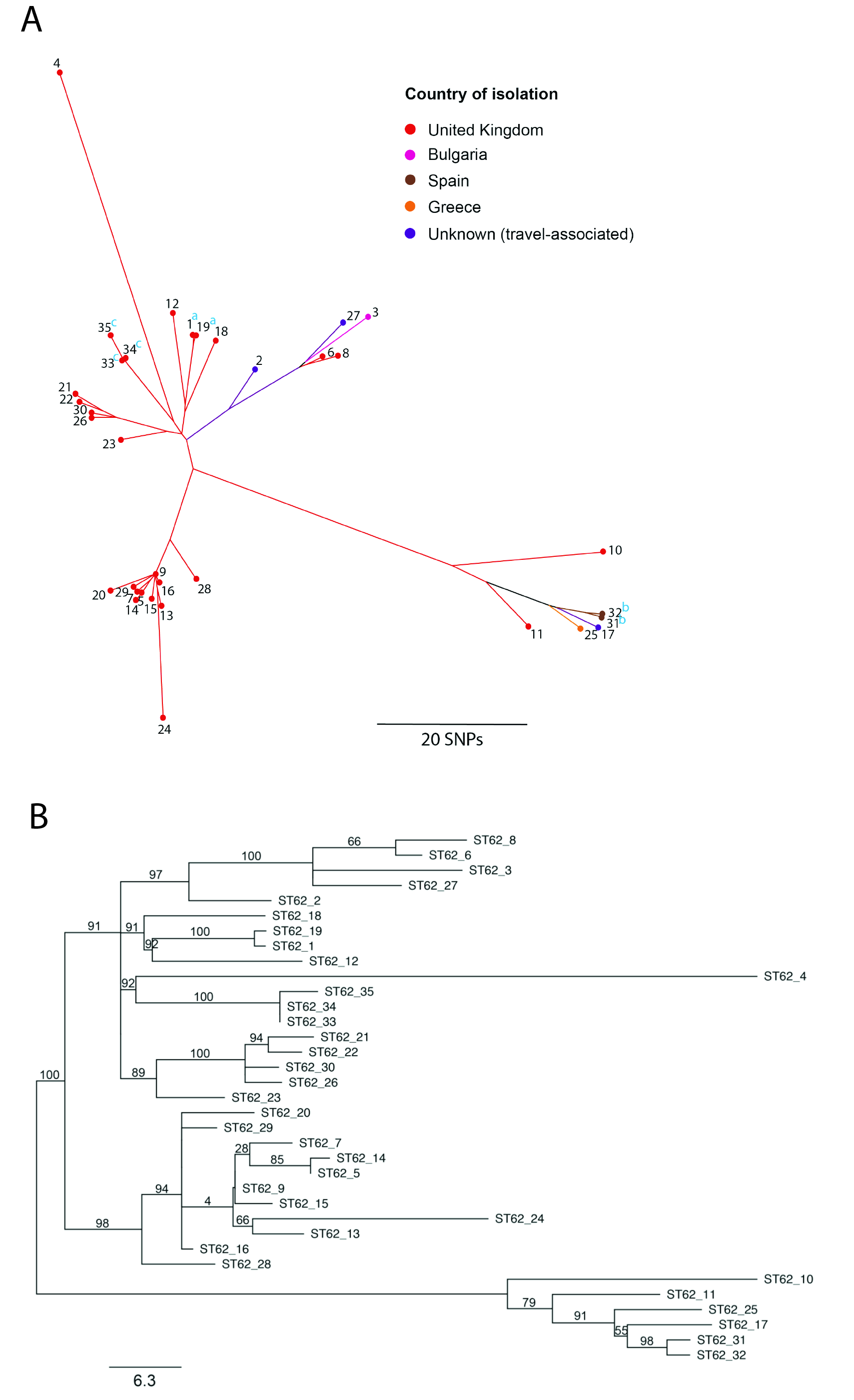

Supplement: Supplemental Material [file supp_gr.209536.116_Supplemental_Fig_S7.tif]

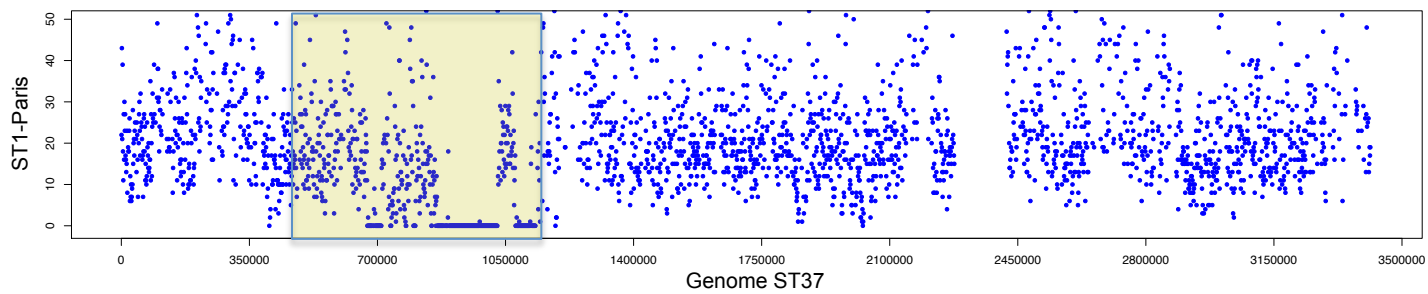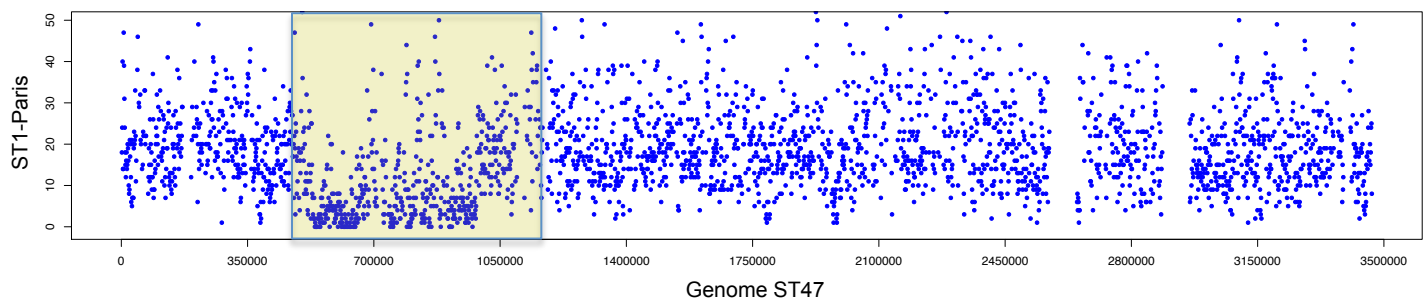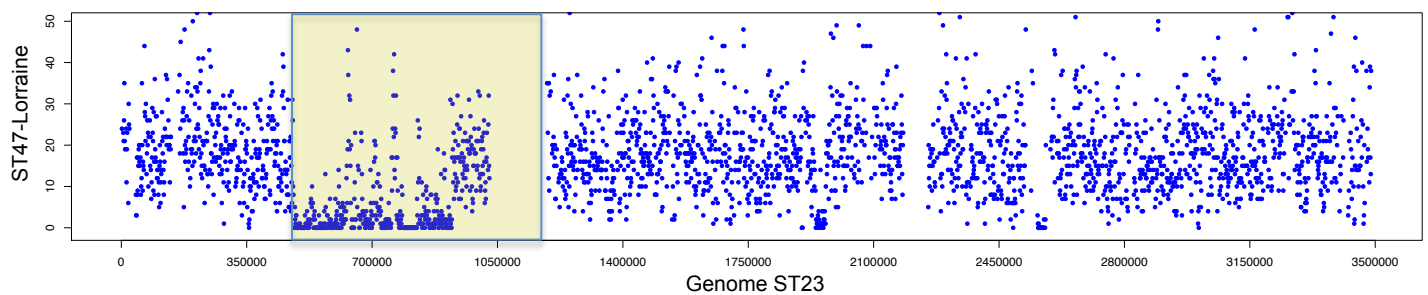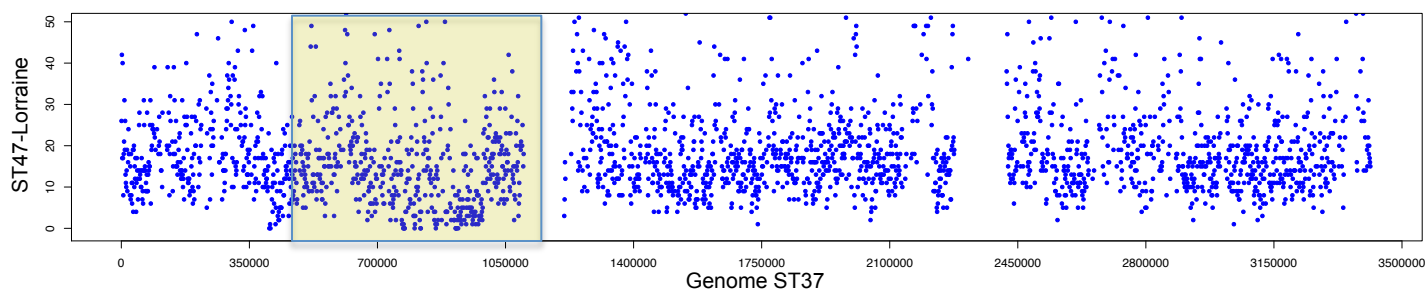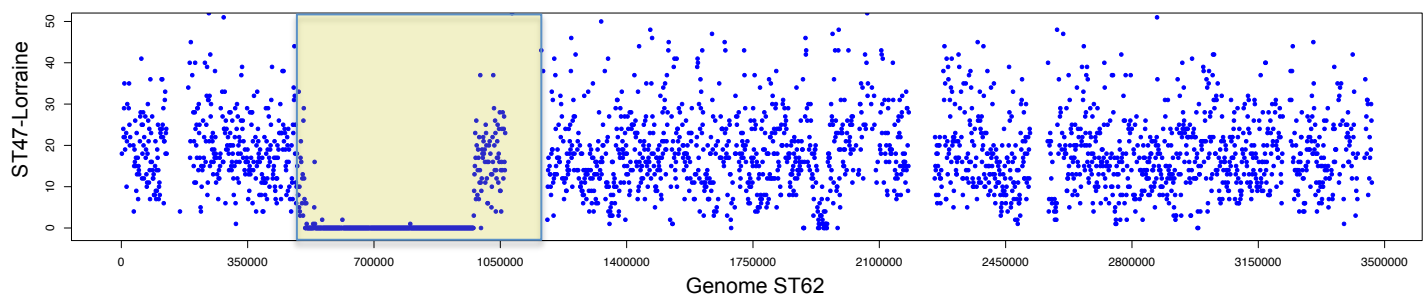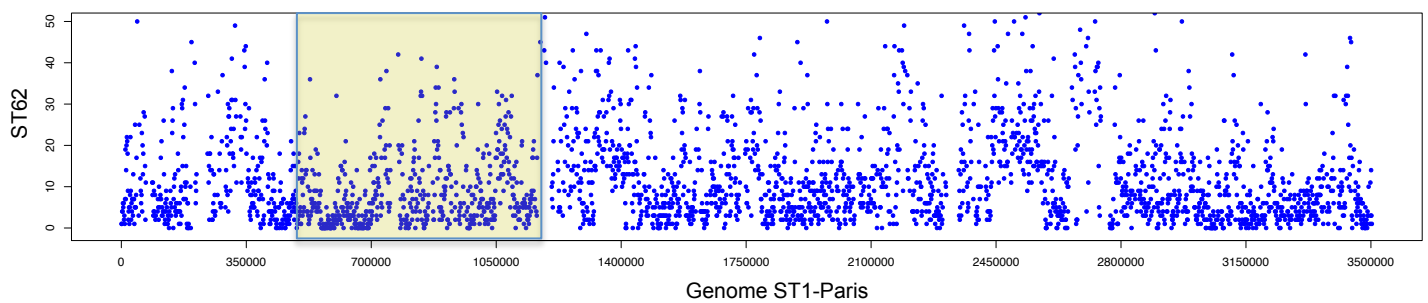

Supplement: Supplemental Material [file supp_gr.209536.116_Supplemental_Fig_S8.pdf]

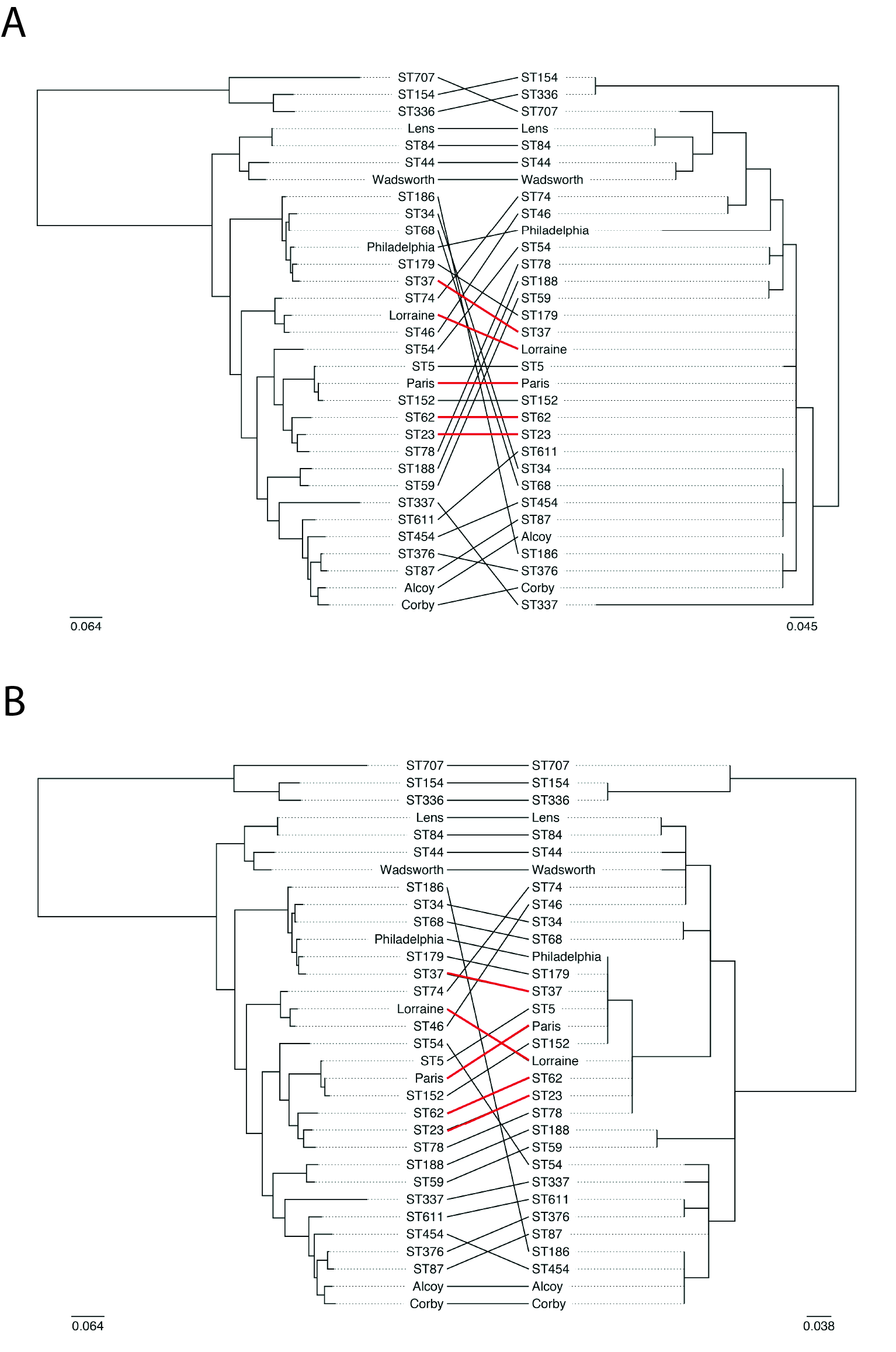

Supplement: Supplemental Material [file supp_gr.209536.116_Supplemental_Fig_S9.tif]
