# Supplementary material for: Multiple major disease-associated clones of Legionella pneumophila have emerged recently and independently
Source: Genome Res. 2016 Nov;26(11):1555–64. doi: 10.1101/gr.209536.116 (PMC5088597; doi:10.1101/gr.209536.116)
Supplement: Supplemental Material [file supp_gr.209536.116_Supplemental_Material_Contents.docx]

**Supplemental Material – Contents**

**Supplemental Methods**

1. Selection of isolates used in this study

2. Culture and DNA extraction

3. Whole genome sequencing

4. Reference genomes

5. Mapping of sequence reads

6. BEAST analysis

7. Estimation of the age of the ST1, ST23, ST47 and ST62 lineages

8. Identification of core genes under positive selection in the five STs

9. Identification of core genes with high nucleotide similarity in the five STs

10. Calculation of pairwise nucleotide similarity between representatives of the five major disease-associated STs

**Supplemental Results**

1. Composition and predicted origin of the recombined regions

2. BEAST analysis

3. Predicting the age of STs 1, 23, 47 and 62

4. Analysis of the gene content

5. Analysis of core genes under positive selection in the five STs

**Supplemental Figures**

**Figure S1.** Distribution of recombination fragment sizes in the four recombining STs (STs 1, 23, 37 and 62). The majority of recombination fragments are small (<10,000bp) although the largest predicted fragment is >129,000bp.

**Figure S2.** Correlation of root-to-tip branch lengths with isolation dates for the five disease-associated STs as calculated by Path-O-Gen. The correlation between root-to-tip distances and sampling time is weak in all lineages, with the exception of the ST37 lineage that possesses a slightly stronger correlation.

**Figure S3.** Midpoint rooted maximum likelihood tree of 71 ST1 and ST1-derived isolates showing bootstrap values calculated from 1000 resamples. The scale indicates the number of SNPs that have occurred for a given branch length.

**Figure S4.** Midpoint rooted maximum likelihood tree of 122 ST47 isolates showing bootstrap values calculated from 1000 resamples. The scale indicates the number of SNPs that have occurred for a given branch length.

**Figure S5.** Unrooted (A) and midpoint rooted (B) maximum likelihood trees of 37 ST23 isolates, with the latter showing bootstrap values calculated from 1000 resamples. In (A), isolates are colored according to the country of isolation and branches are similarly colored to indicate the origin of descendant nodes. Epidemiologically related isolates are indicated by blue superscripted letters (a,b). In both, the scales indicate the number of SNPs that have occurred for a given branch length.

**Figure S6.** Unrooted (A) and midpoint rooted (B) maximum likelihood trees of 72 ST37 isolates (and Philadelphia, an ST36), with the latter showing bootstrap values calculated from 1000 resamples. In (A), isolates are colored according to the country of isolation and branches are similarly colored to indicate the origin of descendant nodes. Epidemiologically related isolates are indicated by blue superscripted letters (a-i). In both, the scales indicate the number of SNPs that have occurred for a given branch length.

**Figure S7.** Unrooted (A) and midpoint rooted (B) maximum likelihood trees of 35 ST62 isolates, with the latter showing bootstrap values calculated from 1000 resamples. In (A), isolates are colored according to the country of isolation and branches are similarly colored to indicate the origin of descendant nodes. Epidemiologically related isolates are indicated by blue superscripted letters (a-c). In both, the scales indicate the number of SNPs that have occurred for a given branch length.

**Figure S8.** The distribution of single-nucleotide polymorphisms (SNPs) along the genome between selected pairs of isolates representative of the five major disease-associated STs. The number of SNPs (y axis) is plotted according to the position of the corresponding 1000bp fragment on the chromosome of the respective strain (x axis). The yellow shaded boxes indicate the chromosomal region identified among the five major disease-associated STs that has significantly higher similarity than the rest of the chromosome.

**Figure S9.** Tanglegrams with maximum likelihood trees of 32 representative STs. The trees were constructed using the whole genome alignment on the left, and individual gene alignments on the right (LPC_0588 (A) and LPC_0671 (B)). The five disease-associated STs are connected with red lines. The scales represent the number of SNPs per site in each alignment.

**Supplemental Tables**

**Table S1.** Whole genome sequenced, previously published *L. pneumophila* isolates that represent the known species diversity.

**Table S2.** *L. pneumophila* isolates belonging to the five major disease-associated STs (ST1, ST23, ST37, ST47, ST62), and additional, closely related isolates (ST18, ST146) used as outgroups. 329 of these are newly sequenced for this study and three have been previously published.

**Table S3.** The reference genomes used and the number of SNPs identified within each of the five disease-associated lineages before recombination is removed.

**Table S4.** Recombined regions in STs 1, 23, 37 and 62.

**Table S5.** Highlighted recombined regions in the SynTView representation of the ST1 lineage.

**Table S6.** Highlighted recombined regions in the SynTView representation of the ST23 lineage.

**Table S7.** Highlighted recombined regions in the SynTView representation of the ST37 lineage.

**Table S8.** Highlighted recombined regions in the SynTView representation of the ST62 lineage.

**Table S9.** Distribution of the known Dot/Icm substrates in the five STs.

**Table S10.** Homoplasic SNPs identified on three or four of the branches of the species tree, leading to STs 1, 23, 37, 47 and 62.

**Table S11.** Genes that are significantly more similar in the five major disease-associated STs than is expected, given the similarity of the five STs across all core genes and also with respect to the conservation of each gene across the species.

**Table S12.** Recombination events that occurred on the branches leading to the ST47 or ST37 lineages. The start and end of the regions are with respect to the ST47 and ST37 mapping references (Lorraine and EUL 132, respectively).
