# Supplementary material for: Multiple major disease-associated clones of Legionella pneumophila have emerged recently and independently
Source: Genome Res. 2016 Nov;26(11):1555–64. doi: 10.1101/gr.209536.116 (PMC5088597; doi:10.1101/gr.209536.116)
Supplement: Supplemental Material [file supp_gr.209536.116_Supplemental_Material_References.docx]

**Supplemental Material – References**

Baele G, Lemey P, Bedford T, Rambaut A, Suchard MA, Alekseyenko AV. Improving the Accuracy of Demographic and Molecular Clock Model Comparison While Accommodating Phylogenetic Uncertainty. Molecular Biology and Evolution. 2012;29(9):2157-67.

Boetzer M, Henkel CV, Jansen HJ, Butler D, Pirovano W. Scaffolding pre-assembled contigs using SSPACE. Bioinformatics. 2011;27(4):578-9.

Boetzer M, Pirovano W. Toward almost closed genomes with GapFiller. Genome Biology. 2012;13(6).

Cazalet C, Rusniok C, Bruggemann H, Zidane N, Magnier A, Ma L, et al. Evidence in the Legionella pneumophila genome for exploitation of host cell functions and high genome plasticity. Nature Genetics. 2004;36(11):1165-73.

Chien MC, Morozova I, Shi SD, Sheng HT, Chen J, Gomez SM, et al. The genomic sequence of the accidental pathogen Legionella pneumophila. Science. 2004;305(5692):1966-8.

D'Auria G, Jimenez-Hernandez, N., Peris-Bondia, F., Moya, A., Latorre, A. Legionella pneumophila pangenome reveals strain-specific virulence factors. Bmc Genomics. 2010;11(181).

Drummond AJ, Suchard MA, Xie D, Rambaut A. Bayesian Phylogenetics with BEAUti and the BEAST 1.7. Molecular Biology and Evolution. 2012;29(8):1969-73.

Gloeckner G, Albert-Weissenberger C, Weinmann E, Jacobi S, Schunder E, Steinert M, et al. Identification and characterization of a new conjugation/type IVA secretion system (trb/tra) of Legionella pneumophila Corby localized on two mobile genomic islands. International Journal of Medical Microbiology. 2008;298(5-6):411-28.

Gomez-Valero L, Rusniok C, Jarraud S, Vacherie B, Rouy Z, Barbe V, et al. Extensive recombination events and horizontal gene transfer shaped the Legionella pneumophila genomes. Bmc Genomics. 2011;12.

Harris SR, Feil EJ, Holden MTG, Quail MA, Nickerson EK, Chantratita N, et al. Evolution of MRSA During Hospital Transmission and Intercontinental Spread. Science. 2010;327(5964):469-74.

Nei M, Li WH. Mathematical model for studying genetic variation in terms of restriction endonucleases. Proceedings of the National Academy of Sciences. 1979;76(10):5269-73.

Page AJ, Cummins CA, Hunt M, Wong VK, Reuter S, Holden MT, et al. Roary: rapid large-scale prokaryote pan genome analysis. Bioinformatics. 2015;31(22):3691-3.

Reuter S, Harrison TG, Koeser CU, Ellington MJ, Smith GP, Parkhill J, et al. A pilot study of rapid whole-genome sequencing for the investigation of a Legionella outbreak. Bmj Open. 2013;3(1).

Sanchez-Buso L, Comas I, Jorques G, Gonzalez-Candelas F. Recombination drives genome evolution in outbreak-related Legionella pneumophila isolates. Nature Genetics. 2014;46(11):1205-11.

Schroeder GN, Petty NK, Mousnier A, Harding CR, Vogrin AJ, Wee B, et al. Legionella pneumophila Strain 130b Possesses a Unique Combination of Type IV Secretion Systems and Novel Dot/Icm Secretion System Effector Proteins. Journal of Bacteriology. 2010;192(22):6001-16.

Stamatakis A. RAxML-VI-HPC: Maximum likelihood-based phylogenetic analyses with thousands of taxa and mixed models. Bioinformatics. 2006;22(21):2688-90.

Underwood AP, Jones G, Mentasti M, Fry NK, Harrison TG. Comparison of the Legionella pneumophila population structure as determined by sequence-based typing and whole genome sequencing. Bmc Microbiology. 2013;13.

Yang Z. PAML 4: Phylogenetic analysis by maximum likelihood. Molecular Biology and Evolution. 2007;24(8):1586-91.

Zerbino DR, Birney E. Velvet: Algorithms for de novo short read assembly using de Bruijn graphs. Genome Research. 2008;18(5):821-9.
