# Supplementary material for: Multiple major disease-associated clones of Legionella pneumophila have emerged recently and independently
Source: Genome Res. 2016 Nov;26(11):1555–64. doi: 10.1101/gr.209536.116 (PMC5088597; doi:10.1101/gr.209536.116)
Supplement: Supplemental Material [file supp_gr.209536.116_Supplemental_Methods.docx]

**Supplementary Methods**

## 1. Selection of isolates used in this study

The newly sequenced isolates constitute all isolates belonging to ST37, ST47 and ST62 present in the culture collection at Public Health England (UK) at the time of selection. Furthermore, ST1, ST23 and ST47 isolates were selected from the culture collection at the Legionella National Reference Centre (France) from the largest possible time period (2000-2012) for which sequence based typing (SBT) data were routinely available and from the most diverse locations. The majority of the isolates are from sporadic cases of Legionnaires’ disease or environments associated with these cases as indicated in **Table S2**.

## 2. Culture and DNA extraction

The newly sequenced isolates were grown at 37°C on buffered charcoal-yeast extract (BCYE) agar for 48 to 72h prior to DNA extraction. High quality DNA was extracted using the Wizard (Promega UK, Southampton, UK), PurElute (VH Bio, Gateshead, UK) or the DNeasy Blood & Tissue Kit (Qiagen), according to manufacturer’s instructions. DNA was eluted in 1x TE pH 8·0 and quantified using Qubit (Life Technologies Ltd, Paisley, UK).

## 3. Whole genome sequencing

Paired-end sequencing on the Illumina HiSeq platform and a read length of 100 bases were used for all isolates, except OLDA1/ST1_31 for which the Illumina MiSeq platform and a read length of 150 bases were used.

## 4. Reference genomes

The previously published Corby genome (Gloeckner *et al.*, 2008) was used as the mapping reference for the 32 isolates representing the species diversity. For in-depth analysis of each ST and to improve resolution, isolates from each of the five STs were mapped to a reference genome of the same ST. These reference genomes were: the complete genome sequence of Paris (ST1) (Cazalet *et al.,* 2004), the newly sequenced and *de novo* assembled ST23 isolate EUL00011 (31 contigs, mean contig length of 112,539bp, and N50 of 242,931bp), the newly sequenced and *de novo* assembled ST37 isolate, EUL00132 (20 contigs, mean contig length of 170,592bp and N50 of 372,526bp), the complete genome sequence of Lorraine (ST47) (Gomez-Valero *et al.*, 2011) and the newly sequenced and *de novo* assembled ST62 isolate, H043540106 (39 contigs, mean contig length of 88,821bp and N50 of 182,977bp). The *de novo* assemblies were generated using the in-house pipeline of the Sanger Institute that uses Velvet (Zerbino & Birney, 2008), SSPACE (Boetzer *et al.,* 2011) and GapFiller (Boetzer & Pirovano, 2012).

## 5. Mapping of sequence reads

Sequence reads were mapped to a reference using SMALT v0·7·4. An in-house pipeline using samtools and bcftools was used to call bases with the following quality metrics: 75% of high quality mapped reads on each strand must agree with the base call, the base quality score must be at least 50 and the mapping quality score must be at least 30. Reads that did not map to one region uniquely were discarded, to avoid repetitive regions. SNPs were identified using a standard approach (Harris *et al.,* 2010).

## 6. BEAST analysis

Time-dependent phylogenetic reconstructions and calculations of evolutionary rates were undertaken using BEAST v1.7 (Drummond *et al.,* 2012). SNP alignments (excluding any SNPs identified to be in recombined regions) and the isolation dates of samples were used as input. A GTR model was used as well as a site heterogeneity model of “Gamma + Invariant” sites. A variety of models were tested including constant, exponential and Bayesian skyline (variable) population sizes and strict, lognormal relaxed, exponential relaxed and random clock models. Two methods, path sampling and stepping stones sampling, were used to assess the model with priors that best fitted the data (Baele *et al.,* 2012). All results were produced from three independent chains of 100 millions steps, sampled every 10,000 steps. The first 10 million steps were discarded as burn-in. LogCombiner was used to combine the results and maximum clade credibility (MCC) trees were constructed using TreeAnnotator. Both programs are part of the BEAST package. Tracer v1.5 (available from <http://tree.bio.ed.ac.uk/software/tracer>) was used to check convergence and ensure all effective sample size (ESS) values were in excess of 200.

## 7. Estimation of the age of the ST1, ST23, ST47 and ST62 lineages

Isolates belonging to ST18, ST146, ST46 and ST78 (**Tables S1 and S2**) were used to establish the root of the ST1, ST23, ST47 and ST62 lineages, respectively. Maximum likelihood trees of each ST, constructed with RAxML v7.0.4 (Stamatakis, 2006) without the out-group and after the removal of recombinant regions from the alignment, were then rooted appropriately. The number of SNPs on each branch of the tree was reconstructed using accelerated transformation parsimony. This means that SNPs are inferred to have occurred as early as possible. The total length of genome affected by recombination events on each branch (as inferred by Gubbins) was calculated. The number of SNPs on the branch were then scaled up by the proportion of the genome removed due to recombination, to take into account additional SNPs that may have arisen from mutation but were located inside recombinant regions. Each branch length was converted to a length of time that it would have taken for the SNPs to occur, using the evolutionary rates calculated for the ST37 lineage and the previously published ST578 lineage (Sanchez-Buso *et al.*, 2014), using the formula:

Length of time for a given branch (years) = no. SNPs on branch / mutation rate (SNPs/genome/year)

The length of time taken for each root-to-tip distance to have occurred was then calculated by summing the lengths of time taken for the constituent branches. Finally, an estimated age of the MRCA was calculated by subtracting the length of time taken for each root-to-tip distance from the sampling date of the respective isolates, and taking the mean of these values.

## 8. Identification of core genes under positive selection in the five STs

An alignment comprising all 364 isolates was prepared for each core gene and also used to generate a maximum likelihood tree using RAxML v7.0.4 (Stamatakis, 2006). For each of the core genes, the individual gene alignment and gene tree were used as input to CodeML (Yang, 2007). The branch-site model was used to test whether any regions within the gene had been subjected to positive selection on the branches leading to each of the five disease-associated STs. Testing was performed five times for every gene, specifying just one of the five branches each time. For each of the five branches, a null model (specifying that the dN/dS does not differ between the branch of interest and the rest of the tree) and an alternative model (specifying that the branch of interest may have some sites under positive selection) were tested and compared using log likelihood values.

## 9. Identification of core genes with high nucleotide similarity in the five STs

The core genome of the ST representatives, with the exclusion of the distantly related STs (ST336, ST154 and ST707), was defined using Roary (Page *et al.*, 2015). For each core gene, a nucleotide alignment was generated using one representative from each of the five STs (Paris/ST1 (Cazalet *et al.*, 2004), EUL00011/ST23_3, EUL00132/ST37_69, Lorraine/ST47 (Gomez-Valero *et al.*, 2011), H043540106/ST62_2) and excluding all other ST representatives. The nucleotide diversity (π) value, a measurement first described by Nei and Li (1979), was calculated for each of these alignments using the R package, “pegas”.

To test whether each core gene possessed significantly higher nucleotide similarity (or lower diversity) in the five major disease-associated STs than would be expected, given the similarity of all core genes between the five disease-associated STs and the conservation of the gene across the species representatives, we compared the nucleotide diversity values to those obtained from sampling all possible combinations of any five STs from the collection of species representatives. However, we excluded the distantly related STs, ST336, ST152 and ST707, and ST5 and ST152, which are nested within ST1, and also the Philadelphia, ST42 and Alcoy strains, as they contribute to many disease cases. Thus, with the remaining 24 STs (including the five major disease-associated STs), nucleotide diversity values were calculated for each of the 42,504 possible combinations of five STs and for each of the 1888 core genes. These were denoted *π_i,j_* i.e. the nucleotide diversity for the *i^th^* gene and *j^th^* ST combination, for *i* = 1,…,1888 and *j* = 1,…,42504.

To account for the phylogenetic distance between a given set of five STs, each nucleotide diversity value obtained was adjusted by dividing by the median nucleotide diversity value across all core genes for that particular combination of five STs. This is given by the equation:


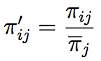


where *π_i,j_* is the nucleotide diversity for the *i^th^* gene and *j^th^* ST combination, and where for each *j* = 1,…,42504


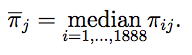


Using the adjusted nucleotide diversity value of the five major disease-associated STs together with the 42,504 adjusted values calculated for all combinations of five STs, a *p-value* was determined for the each (*i^th^*) core gene with the formula:


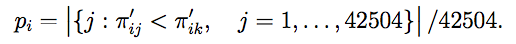


where we assume that the *k^th^* combination of STs is the one corresponding to the five disease-associated STs.

Correction for multiple testing was performed using the Benjamini-Hochberg method and implemented in R.

## 10. Calculation of pairwise nucleotide similarity between representatives of the five major disease-associated STs

Each genome was fragmented into contiguous and non-overlapping 1kb segments. Each segment was compared to other genomes using BLASTn, and the number of SNPs between each query sequence and a full-length match sequence were recorded.
