# Supplementary material for: Multiple major disease-associated clones of Legionella pneumophila have emerged recently and independently
Source: Genome Res. 2016 Nov;26(11):1555–64. doi: 10.1101/gr.209536.116 (PMC5088597; doi:10.1101/gr.209536.116)
Supplement: Supplemental Material [file supp_gr.209536.116_Supplemental_Results.docx]

# Supplemental Results

## 1. Composition and predicted origin of the recombined regions

The distribution of lengths of the recombined regions identified in the ST1, ST23, ST37 and ST62 lineages is shown in **Fig. S1**. The mean length ranges from 15,809bp (ST1) to 24,420bp (ST37), although the largest predicted region is over 129kb. The recombined regions comprise mainly integrative conjugative elements (ICE) that carry *tra*-like gene clusters, phage-like elements and the LPS-encoding region in the ST1 lineage. Analyses of the origin of the recombined regions within each of these lineages showed that no recombination comes from outside of the species and very few regions come from the subspecies *L. pneumophila* *fraseri,* which comprises ST154, ST336 and ST707 (one region each in the ST23 and ST62 lineages). A relatively large proportion, ranging from 25·0% (ST37 lineage) to 61·5% (ST62 lineage), comes from closely related isolates in the same major clade of the *L.* *pneumophila pneumophila* subspecies, to which the five STs belong, although recombination also occurs between major clades. Furthermore, only a small number of the recombined regions are derived from another of the five major disease-associated STs.

## 2. BEAST analysis

Since only the ST37 lineage was shown to possess some correlation between the root-to-tip distances and sampling date (**Fig. S2**), we proceeded to date only this lineage using the Bayesian coalescent model in BEAST (Drummond *et al.*, 2012). This method can use a relaxed molecular clock rather than assuming a constant clock. We tested and compared various combinations of model parameters and found that a model that uses a Bayesian skyline (variable) population size and an exponential relaxed evolutionary rate has the best fit to the data. Using this model, the median age of emergence was estimated to be 1979 (95% highest posterior density (HPD) intervals: 1968 to 1985) (**Fig. 3**). The earliest recorded ST37 isolate in the SBT database is from 1982, three years after the most likely emergence date, an observation that makes the dating results from BEAST seem plausible. The evolutionary rate estimated by BEAST is 2·07x10^-7^ substitutions per site per year (95% HPD interval: 1·69x10^-7^ to 2·44x10^-7^), slightly higher than that of the *L. pneumophila* ST578 lineage (1·39 x 10^-7^), calculated in a previous study (Sanchez-Buso *et al*., 2014).

## 3. Predicting the age of STs 1, 23, 47 and 62

Since the lack of observed temporal signal prohibited us from using Path-O-Gen or BEAST to date the emergence of STs 1, 23, 47 and 62, we used the estimated evolutionary rates of the ST578 and ST37 lineages to provide approximations of the length of time it would have taken for the observed diversity to arise. This analysis suggested emergence dates of 1851/1899 for ST1, 1972/1983 for ST23, 1943/1964 for ST62 and 1998/2002 for ST47, with the two dates provided corresponding to the application of the ST578 and ST37 mean evolutionary rates, respectively. Although the date of the first sampled isolate of ST47 was 1994, a few years before our mean predicted emergence date, this does fall within the dates predicted by the 95% HPD intervals on the evolutionary rate. Even allowing for possible large variations in the evolutionary rate between lineages, our results undoubtedly support a very recent emergence of all five major disease-associated STs, with ST1 being the oldest and ST47 the most recent.

## 4. Analysis of the gene content

No genes were identified that were present only in the five STs and not in any other genomes from the species-wide collection. However, we did identify 6, 17, 5, 24 and 23 genes that were specific to ST1, ST23, ST37, ST47 and ST62, respectively. The majority of these genes encode transposases, phage-related proteins and hypothetical proteins, and thus bioinformatics predictions on their disease impact are not possible. We also noted that all five disease-associated STs encode specific multidrug transporters, putative efflux pumps for toxic compounds and heavy metals, and members of the major facilitator family, which might provide these clones with a survival advantage in hostile environments.

No new Dot/Icm effectors were identified amongst the five STs. However, the conservation of the 306 known Dot/Icm substrates was high across the five STs (**Table S9**), with only thirteen missing in STs 1, 23, 47 and 62 (and all present in ST37).

## 5. Analysis of core genes under positive selection in the five STs

Amongst the 1538 core genes identified in all 364 isolates, we searched for any genes that have been subjected to positive selection in the recent evolution of the five STs, which may indicate common adaptation to a particular niche. We used the branch-site model in CodeML (Yang, 2007) to identify genes in which the dN/dS ratio was significantly higher on the individual branches leading to each of the five STs than on the rest of the species tree. However, no evidence of shared positive selection was found within the core genes using this method. While this result may indicate a true lack of shared positive selection between the five STs within core genes, we also acknowledge limitations to the method such as the inability to disregard the four remaining branches leading to the disease-associated STs when comparing a particular branch to the rest of the species tree. Thus we also sought other methods for identifying possible convergent evolution between the five STs.
