# Supplementary material for: Multiple major disease-associated clones of Legionella pneumophila have emerged recently and independently
Source: Genome Res. 2016 Nov;26(11):1555–64. doi: 10.1101/gr.209536.116 (PMC5088597; doi:10.1101/gr.209536.116)
Supplement: Supplemental Material [file supp_gr.209536.116_Supplemental_Table_S1.docx]

Table S1. Whole genome sequenced, previously published *L. pneumophila* isolates that represent the known species diversity.

| **Isolate name** | **Other name** | **ST** | **Sg** | **Source** | **Country** | **Year** | **Reference** | **Known epidemiological relatedness** |
| --- | --- | --- | --- | --- | --- | --- | --- | --- |
| Alcoy |  | 578 | 1 | clin | Spain | 1999 | D’Auria et al. 2010 | None |
| Corby |  | 51 | 1 | clin | UK | 1982 | Gloeckner et al. 2008 | None |
| Lorraine | ST47_1 | 47 | 1 | clin | France | 2004 | Gomez-Valero et al. 2011 | None |
| Philadelphia1  (ATCC 33152) |  | 36 | 1 | clin | USA | 1981 | Chien et al. 2004 | None |
| Wadsworth 130b |  | 42 | 1 | clin | USA |  | Schroeder et al. 2010 | None |
| LC6774 |  | 154 | 1 | env | UK | 2003 | Underwood et al. 2013 | None |
| H093380153 |  | 179 | 1 | clin | UK | 2009 | Underwood et al. 2013 | None |
| H044500045 |  | 186 | 1 | clin | UK | 2004 | Underwood et al. 2013 | None |
| H075160080 |  | 188 | 1 | env | UK | 2007 | Underwood et al. 2013 | None |
| H063280001 | ST23_1 | 23 | 1 | clin | UK | 2006 | Underwood et al. 2013 | None |
| Lansing-3 |  | 336 | 15 | clin | USA | 1981 | Underwood et al. 2013 | None |
| RR08000517 |  | 337 | 4 | env | UK | 2007 | Underwood et al. 2013 | None |
| RR08000134 |  | 34 | 1 | env | UK | 2005 | Underwood et al. 2013 | None |
| RR08000760 |  | 376 | 4 | env | UK | 2006 | Underwood et al. 2013 | None |
| H100260089 |  | 44 | 1 | clin | UK | 2010 | Underwood et al. 2013 | None |
| H091960011 |  | 454 | 1 | env | UK | 2009 | Underwood et al. 2013 | None |
| H093620212 |  | 46 | 1 | clin | UK | 2009 | Underwood et al. 2013 | None |
| H065000139 |  | 54 | 1 | clin | UK | 2006 | Underwood et al. 2013 | None |
| H070840415 |  | 59 | 1 | clin | UK | 2007 | Underwood et al. 2013 | None |
| H090500162 |  | 611 | 1 | env | UK | 2009 | Underwood et al. 2013 | None |
| H064180002 | ST62_1 | 62 | 1 | clin | UK | 2006 | Underwood et al. 2013 | Related to ST62_19 |
| H074360710 |  | 68 | 6 | env | UK | 2007 | Underwood et al. 2013 | None |
| H091960009 |  | 707 | 4 | env | UK | 2009 | Underwood et al. 2013 | None |
| LC6451 |  | 78 | 1 | clin | UK | 2002 | Underwood et al. 2013 | None |
| H071260094 |  | 87 | 3 | clin | Spain | 2007 | Underwood et al. 2013 | None |
| H053260229 |  | 74 | 1 | clin | UK | 2005 | Underwood et al. 2013 | None |
| H043940028 |  | 84 | 1 | clin | UK | 2004 | Underwood et al. 2013 | None |
| Paris | ST1_1 | 1 | 1 | clin | France | 2002 | Cazalet et al. 2004 | None |
| H074360702 | ST152_1 | 152 | 1 | env | UK | 2007 | Underwood et al. 2013 | None |
| EUL00013 | ST5_1 | 5 | 1 | clin | UK | 1994 | Underwood et al. 2013 | None |
| EUL00165 | ST37_1 | 37 | 1 | clin | UK | 2003 | Underwood et al. 2013 | Related to ST37_64 |
| Lens |  | 15 | 1 | clin | France | 2003 | Cazalet et al. 2004 | None |

ST, sequence type

Sg, serogroup

clin, clinical

env, environmental
