# Supplementary material for: Multiple major disease-associated clones of Legionella pneumophila have emerged recently and independently
Source: Genome Res. 2016 Nov;26(11):1555–64. doi: 10.1101/gr.209536.116 (PMC5088597; doi:10.1101/gr.209536.116)
Supplement: Supplemental Material [file supp_gr.209536.116_Supplemental_Table_S2.docx]

Table S2. *L. pneumophila* isolates belonging to the five major disease-associated STs (ST1, ST23, ST37, ST47, ST62), and additional, closely related isolates (ST18, ST146) used as outgroups. 329 of these are newly sequenced for this study and three have been previously published.

| **Isolate name** | **Other name** | **ST** | **Sg** | **Source** | **Country** | **Year** | **Known epidemiological relatedness** | **Accession number/**  **Reference** |
| --- | --- | --- | --- | --- | --- | --- | --- | --- |
| H034800423 | ST1_2 | 1 | 1 | env | UK | 2003 | None | Reuter et al. 2013 |
| EUL00055 | ST1_3 | 1 | 1 | clin | Spain | 1994 | Related to ST1_15 | ERR332141 |
| EUL00088 | ST1_4 | 1 | 1 | clin | Denmark | 1995 | None | ERR332174 |
| EUL00093 | ST1_5 | 1 | 1 | clin | Denmark | 1992 | Related to ST1_24 and ST1_25 | ERR332179 |
| EUL00010 | ST1_6 | 1 | 1 | env | Switzerland | 1989 | Related to ST1_9 and ST1_20 | ERR376635 |
| EUL00001 | ST1_7 | 1 | 1 | clin | Switzerland | 1998 | None | ERR376626 |
| EUL00021 | ST1_8 | 1 | 1 | env | UK | 1999 | None | ERR376638 |
| EUL00003 | ST1_9 | 1 | 1 | clin | Switzerland | 1989 | Related to ST1_6 and ST1_20 | ERR376628 |
| EUL00109 | ST1_10 | 1 | 1 | env | Sweden | 1992 | None | ERR376662 |
| EUL00042 | ST1_11 | 1 | 1 | clin | Italy | 1999 | None | ERR376667 |
| EUL00043 | ST1_12 | 1 | 1 | clin | Italy | 1999 | None | ERR376668 |
| EUL00044 | ST1_13 | 1 | 1 | env | Italy | 1999 | Related to ST1_28 | ERR376669 |
| EUL00046 | ST1_14 | 1 | 1 | env | Italy | 1999 | None | ERR376671 |
| EUL00058 | ST1_15 | 1 | 1 | env | Spain | 1994 | Related to ST1_3 | ERR376683 |
| EUL00060 | ST1_16 | 1 | 1 | clin | Greece | 1992 | None | ERR376685 |
| EUL00062 | ST1_17 | 1 | 1 | env | Greece | 1989 | None | ERR376687 |
| EUL00067 | ST1_18 | 1 | 1 | clin | Greece | 1995 | None | ERR376692 |
| EUL00085 | ST1_19 | 1 | 1 | clin | Denmark | 1995 | None | ERR376710 |
| EUL00009 | ST1_20 | 1 | 1 | env | Switzerland | 1989 | Related to ST1_6 and ST1_9 | ERR376634 |
| EUL00082 | ST1_21 | 1 | 1 | clin | Denmark | 1994 | None | ERR376733 |
| EUL00084 | ST1_22 | 1 | 1 | clin | Denmark | 1995 | None | ERR376735 |
| EUL00090 | ST1_23 | 1 | 1 | clin | Denmark | Unknown | None | ERR376736 |
| EUL00094 | ST1_24 | 1 | 1 | clin | Denmark | 1992 | Related to ST1_5 and ST1_25 | ERR376738 |
| EUL00095 | ST1_25 | 1 | 1 | env | Denmark | 1993 | Related to ST1_5 and ST1_24 | ERR376739 |
| EUL00104 | ST1_26 | 1 | 1 | clin | Sweden | 1992 | None | ERR376745 |
| EUL00108 | ST1_27 | 1 | 1 | clin | Sweden | 1992 | None | ERR376748 |
| EUL00037 | ST1_28 | 1 | 1 | clin | Italy | 1999 | Related to ST1_13 | ERR376723 |
| EUL00119 | ST1_29 | 1 | 1 | clin | Germany | 2005 | None | ERR376757 |
| EUL00053 | ST1_30 | 1 | 1 | clin | Spain | 1995 | None | ERR376725 |
| OLDA (NCTC1208) | ST1_31 | 1 | 1 | clin | USA | 1947 | None | ERR434061 |
| HL00364001 | ST1_32 | 1 | 1 | clin | France | 2000 | None | ERR922483 |
| HL02304015 | ST1_33 | 1 | 1 | clin | France | 2002 | None | ERR922484 |
| HL03111005 | ST1_34 | 1 | 1 | env | France | 2003 | None | ERR922485 |
| HL03373012 | ST1_35 | 1 | 1 | env | France | 2003 | None | ERR922486 |
| HL04163014 | ST1_36 | 1 | 1 | clin | France | 2004 | None | ERR922487 |
| HL07013004 | ST1_37 | 1 | 1 | env | France | 2007 | None | ERR922488 |
| LG09192006 | ST1_38 | 1 | 1 | clin | France | 2009 | None | ERR922489 |
| LG09404015 | ST1_39 | 1 | 1 | clin | France | 2009 | None | ERR922490 |
| LG10191002 | ST1_40 | 1 | 1 | clin | France | 2010 | Related to ST1_41 | ERR922491 |
| LG10203012 | ST1_41 | 1 | 1 | env | France | 2010 | Related to ST1_40 | ERR922492 |
| LG11011012 | ST1_42 | 1 | 1 | env | France | 2010 | None | ERR922493 |
| LG11054025 | ST1_43 | 1 | 1 | env | France | 2011 | None | ERR922494 |
| LG11181044 | ST1_44 | 1 | 1 | env | Morocco | 2009 | None | ERR922495 |
| LG11391124 | ST1_45 | 1 | 1 | env | France | 2011 | None | ERR922496 |
| LP21 | ST1_46 | 1 | 1 | clin | Sweden | 1996-1999 | Unknown | ERR922497 |
| LP23 | ST1_47 | 1 | 1 | clin | Sweden | 1996-2000 | None | ERR922498 |
| LT 40/04 | ST1_48 | 1 | 1 | clin | Austria | 2004 | None | ERR922499 |
| NIIB223 | ST1_49 | 1 | 1 | env | Japan | 1986 | Unknown | ERR922500 |
| NIIB225 | ST1_50 | 1 | 1 | env | Japan | 1986 | Unknown | ERR922501 |
| L 3386/03 | ST1_51 | 1 | 1 | env | Austria | 2003 | None | ERR922502 |
| L 3415/03 | ST1_52 | 1 | 1 | env | Austria | 2003 | None | ERR922503 |
| LG10143009 | ST1_53 | 1 | 1 | clin | France | 2010 | None | ERR922504 |
| NIIB80 | ST1_54 | 1 | 1 | clin | Japan | 1981 | None | ERR923392 |
| LP22 | ST1_55 | 1 | 1 | clin | Sweden | 1996-1999 | Unknown | ERR923393 |
| L00-549 | ST1_56 | 1 | 1 | clin | Germany | 2000 | None | ERR923394 |
| E21203 | ST1_57 | 1 | 1 | clin | France | 2004 | None | ERR923395 |
| 2735 | ST1_58 | 1 | 1 | env | USA | 2002 | None | ERR923396 |
| Wien 47-14 | ST1_59 | 1 | 1 | env | Austria | 1996 | None | ERR923397 |
| EUL00014 | ST5_2 | 5 (1) | 1 | clin | UK | 1984 | None | ERR376639 |
| EUL00016 | ST5_3 | 5 (1) | 1 | clin | UK | 1984 | None | ERR376641 |
| EUL00017 | ST7_1 | 7 (1) | 1 | clin | UK | 1993 | None | ERR376642 |
| EUL00113 | ST7_2 | 7 (1) | 1 | env | Germany | 1995 | None | ERR376751 |
| EUL00114 | ST7_3 | 7 (1) | 1 | env | Germany | 1995 | None | ERR376752 |
| EUL00045 | ST72_1 | 72 (1) | 1 | clin | Italy | 1999 | None | ERR376670 |
| EUL00110 | ST10_1 | 10 (1) | 1 | clin | Germany | 1993 | None | ERR376674 |
| EUL00117 | ST6_1 | 6 (1) | 1 | clin | Germany | 2005 | None | ERR376755 |
| EUL00157 | ST8_1 | 8 (1) | 1 | env | UK | 2004 | None | ERR376779 |
| IN-23-G1-C2 (ATCC 35289) | ST390_1 | 390 (1) | 9 | env | Netherlands | 1988 | None | ERR923391 |
| EUL00008 | ST23_2 | 23 | 1 | clin | Switzerland | 1993 | Related to ST23_3 and ST23_4 | ERR376633 |
| EUL00011 | ST23_3 | 23 | 1 | env | Switzerland | 1993 | Related to ST23_2 and ST23_4 | ERR376636 |
| EUL00012 | ST23_4 | 23 | 1 | env | Switzerland | 1993 | Related to ST23_2 and ST23_3 | ERR376637 |
| EUL00041 | ST23_5 | 23 | 1 | clin | Italy | 1999 | None | ERR376666 |
| EUL00130 | ST23_6 | 23 | 1 | clin | Croatia | 1987 | Related to ST23_7 | ERR376703 |
| EUL00129 | ST23_7 | 23 | 1 | clin | Croatia | 1987 | Related to ST23_6 | ERR376762 |
| EUL00004 | ST23_8 | 23 | 1 | clin | Switzerland | 1991 | None | ERR376721 |
| EUL00028 | ST23_9 | 23 | 1 | clin | France | 1994 | None | ERR376722 |
| HL01273027 | ST23_10 | 23 | 1 | clin | France | 2001 | None | ERR922505 |
| HL02365014 | ST23_11 | 23 | 1 | clin | France | 2002 | None | ERR922506 |
| HL02365015 | ST23_12 | 23 | 1 | clin | France | 2002 | None | ERR922507 |
| HL03071012 | ST23_13 | 23 | 1 | clin | France | 2003 | None | ERR922508 |
| HL03393028 | ST23_14 | 23 | 1 | clin | France | 2003 | None | ERR922509 |
| HL04371017 | ST23_15 | 23 | 1 | clin | France | 2004 | None | ERR922510 |
| HL04433031 | ST23_16 | 23 | 1 | clin | France | 2004 | None | ERR922511 |
| HL05063005 | ST23_17 | 23 | 1 | clin | France | 2005 | None | ERR922512 |
| HL05322037 | ST23_18 | 23 | 1 | env | France | 2005 | None | ERR922513 |
| HL05415018 | ST23_19 | 23 | 1 | clin | France | 2005 | None | ERR922514 |
| HL06043045 | ST23_20 | 23 | 1 | clin | France | 2006 | None | ERR922515 |
| HL06373021 | ST23_21 | 23 | 1 | clin | France | 2006 | None | ERR922516 |
| HL07093017 | ST23_22 | 23 | 1 | clin | France | 2007 | None | ERR922517 |
| LG07512008 | ST23_23 | 23 | 1 | clin | France | 2007 | None | ERR922518 |
| LG08345006 | ST23_24 | 23 | 1 | clin | France | 2008 | None | ERR922519 |
| LG08392025 | ST23_25 | 23 | 1 | clin | France | 2008 | None | ERR922520 |
| LG09153012 | ST23_26 | 23 | 1 | env | France | 2009 | None | ERR922521 |
| LG09353013 | ST23_27 | 23 | 1 | env | France | 2009 | None | ERR922522 |
| LG09403015 | ST23_28 | 23 | 1 | clin | France | 2009 | None | ERR922523 |
| LG09454021 | ST23_29 | 23 | 1 | clin | France | 2009 | None | ERR922524 |
| LG10255002 | ST23_30 | 23 | 1 | clin | France | 2010 | None | ERR922525 |
| LG10363013 | ST23_31 | 23 | 1 | env | France | 2010 | None | ERR922526 |
| LG10481020 | ST23_32 | 23 | 1 | clin | France | 2010 | None | ERR922527 |
| LG11272006 | ST23_33 | 23 | 1 | clin | France | 2011 | None | ERR922528 |
| LG11363009 | ST23_34 | 23 | 1 | clin | France | 2011 | None | ERR922529 |
| LG11402026 | ST23_35 | 23 | 1 | clin | France | 2011 | None | ERR922530 |
| LG12242012 | ST23_36 | 23 | 1 | clin | France | 2012 | None | ERR922531 |
| LG12465006 | ST23_37 | 23 | 1 | clin | France | 2012 | None | ERR922532 |
| H064240448 | ST37_2 | 37 | 1 | env | UK | 2006 | None | ERR363849 |
| LC0731 | ST37_3 | 37 | 1 | clin | UK | 1989 | Related to ST37_4, ST37_5, ST37_59, ST37_61 and ST37_63 | ERR363882 |
| LC0732 | ST37_4 | 37 | 1 | clin | UK | 1989 | Related to ST37_3, ST37_5, ST37_59, ST37_61 and ST37_63 | ERR363883 |
| LC0763 | ST37_5 | 37 | 1 | env | UK | 1989 | Related to ST37_3, ST37_4, ST37_59, ST37_61 and ST37_63 | ERR363884 |
| LC5694 | ST37_6 | 37 | 1 | clin | UK | 2000 | None | ERR363891 |
| LC5722 | ST37_7 | 37 | 1 | clin | UK | 2000 | None | ERR363892 |
| LC5738 | ST37_8 | 37 | 1 | clin | UK | 2000 | None | ERR363893 |
| LC5755 | ST37_9 | 37 | 1 | clin | UK | 2000 | None | ERR363894 |
| LC5908 | ST37_10 | 37 | 1 | clin | UK | 2001 | None | ERR363895 |
| LC6163 | ST37_11 | 37 | 1 | clin | UK | 2002 | None | ERR363897 |
| LC6267 | ST37_12 | 37 | 1 | clin | UK | 2002 | None | ERR363899 |
| LC6268 | ST37_13 | 37 | 1 | clin | UK | 2002 | None | ERR363900 |
| LC6228 | ST37_14 | 37 | 1 | clin | UK | 2002 | None | ERR363898 |
| H041380048 | ST37_15 | 37 | 1 | clin | UK | 2004 | Related to ST37_23 | ERR363843 |
| H042960010 | ST37_16 | 37 | 1 | clin | UK | 2004 | None | ERR363845 |
| H061140013 | ST37_17 | 37 | 1 | clin | UK | 2006 | None | ERR363847 |
| H071880001 | ST37_18 | 37 | 1 | clin | UK | 2007 | None | ERR363850 |
| H073060003 | ST37_19 | 37 | 1 | clin | UK | 2007 | None | ERR363851 |
| H080820009 | ST37_20 | 37 | 1 | clin | UK | 2008 | None | ERR363853 |
| LC6058 | ST37_21 | 37 | 1 | clin | Unknown (TA) | 2001 | None | ERR363896 |
| LC6293 | ST37_22 | 37 | 1 | clin | Unknown (TA) | 2002 | None | ERR363901 |
| H041640791 | ST37_23 | 37 | 1 | env | UK | 2004 | Related to ST37_15 | ERR363844 |
| LC6788 | ST37_24 | 37 | 1 | clin | Unknown (TA) | 2003 | None | ERR363902 |
| H062660463 | ST37_25 | 37 | 1 | clin | Unknown (TA) | 2006 | None | ERR363848 |
| H073900557 | ST37_26 | 37 | 1 | clin | Unknown (TA) | 2007 | None | ERR363852 |
| LC1127 | ST37_27 | 37 | 1 | clin | UK | 1989 | None | ERR363890 |
| H084760449 | ST37_28 | 37 | 1 | clin | UK | 2008 | None | ERR363857 |
| H085020185 | ST37_29 | 37 | 1 | clin | UK | 2008 | None | ERR363858 |
| H090320386 | ST37_30 | 37 | 1 | clin | UK | 2009 | None | ERR363859 |
| H044260061 | ST37_31 | 37 | 1 | env | UK | 2004 | None | ERR363846 |
| H093140322 | ST37_32 | 37 | 1 | clin | UK | 2009 | Related to ST37_33 | ERR363861 |
| H093160422 | ST37_33 | 37 | 1 | env | UK | 2009 | Related to ST37_32 | ERR363862 |
| H092760433 | ST37_34 | 37 | 1 | clin | Unknown (TA) | 2009 | None | ERR363860 |
| H100940111 | ST37_35 | 37 | 1 | clin | UK | 2010 | None | ERR363863 |
| H101760092 | ST37_36 | 37 | 1 | clin | UK | 2010 | None | ERR363864 |
| H101820190 | ST37_37 | 37 | 1 | clin | UK | 2010 | None | ERR363865 |
| H102020414 | ST37_38 | 37 | 1 | clin | UK | 2010 | None | ERR363867 |
| H101980130 | ST37_39 | 37 | 1 | clin | Unknown (TA) | 2010 | None | ERR363866 |
| H103820081 | ST37_40 | 37 | 1 | clin | UK | 2010 | None | ERR363868 |
| H120240685 | ST37_41 | 37 | 1 | clin | Slovenia | 2010 | None | ERR363992 |
| H104320293 | ST37_42 | 37 | 1 | env | UK | 2010 | None | ERR363869 |
| H113180118 | ST37_43 | 37 | 1 | clin | UK | 2011 | Related to ST37_44 | ERR363871 |
| H113340664 | ST37_44 | 37 | 1 | env | UK | 2011 | Related to ST37_43 | ERR363873 |
| H113280076 | ST37_45 | 37 | 1 | clin | UK | 2011 | None | ERR363872 |
| H113660550 | ST37_46 | 37 | 1 | clin | UK | 2011 | None | ERR363874 |
| H114740454 | ST37_47 | 37 | 1 | clin | UK | 2011 | None | ERR363876 |
| H115040456 | ST37_48 | 37 | 1 | clin | UK | 2011 | None | ERR363877 |
| H111580389 | ST37_49 | 37 | 1 | clin | UK | 2011 | None | ERR363870 |
| H113780240 | ST37_50 | 37 | 1 | clin | Unknown (TA) | 2011 | None | ERR363875 |
| H083920177 | ST37_51 | 37 | 1 | clin | UK | 2008 | Related to ST37_52 | ERR363855 |
| H084140691 | ST37_52 | 37 | 1 | env | UK | 2008 | Related to ST37_51 | ERR363856 |
| H081180019 | ST37_53 | 37 | 1 | env | UK | 2008 | None | ERR363854 |
| H103260667 | ST37_54 | 37 | 1 | env | Greece | 2010 | None | ERR363938 |
| LC464 | ST37_55 | 37 | 1 | clin | UK | 1987 | None | ERR363878 |
| LC0512 | ST37_56 | 37 | 1 | clin | Unknown (TA) | 1988 | None | ERR363879 |
| LC0565 | ST37_57 | 37 | 1 | clin | UK | 1988 | Related to ST37_58, ST37_69, ST37_ST37_70 and ST37_71 | ERR363880 |
| LC0583 | ST37_58 | 37 | 1 | clin | UK | 1988 | Related to ST37_57, ST37_69, ST37_ST37_70 and ST37_71 | ERR363881 |
| LC0782 | ST37_59 | 37 | 1 | clin | UK | 1989 | Related to ST37_3, ST37_4, ST37_5, ST37_61 and ST37_63 | ERR363885 |
| LC0794 | ST37_60 | 37 | 1 | clin | UK | 1989 | Related to ST37_62 | ERR363886 |
| LC0795 | ST37_61 | 37 | 1 | clin | UK | 1989 | Related to ST37_3, ST37_4, ST37_5, ST37_59 and ST37_63 | ERR363887 |
| LC0798 | ST37_62 | 37 | 1 | clin | UK | 1989 | Related to ST37_60 | ERR363888 |
| LC0801 | ST37_63 | 37 | 1 | clin | UK | 1989 | Related to ST37_3, ST37_4, ST37_5, ST37_59 and ST37_61 | ERR363889 |
| EUL166/LP056 | ST37_64 | 37 | 1 | env | UK | 2003 | Related to ST37_1 | ERR364007 |
| EUL00069 | ST37_65 | 37 | 1 | clin | UK | 1995 | None | ERR332155 |
| EUL00073 | ST37_66 | 37 | 1 | clin | UK | 1996 | Related to ST37_67 and ST37_68 | ERR332159 |
| EUL00078 | ST37_67 | 37 | 1 | clin | UK | 1996 | Related to ST37_66 and ST37_68 | ERR340955 |
| EUL00079 | ST37_68 | 37 | 1 | clin | UK | 1996 | Related to ST37_66 and ST37_67 | ERR340956 |
| EUL00132 | ST37_69 | 37 | 1 | clin | UK | 1988 | Related to ST37_57, ST37_58, ST37_70 and ST37_71 | ERR332168 |
| EUL00133 | ST37_70 | 37 | 1 | clin | UK | 1988 | Related to ST37_57, ST37_58, ST37_69 and ST37_71 | ERR332169 |
| EUL00134 | ST37_71 | 37 | 1 | clin | UK | 1988 | Related to ST37_57, ST37_58, ST37_69 and ST37_70 | ERR332170 |
| EUL00131 | ST37_72 | 37 | 1 | clin | UK | 1988 | None | ERR332167 |
| EUL00169 | ST47_2 | 47 | 1 | clin | UK | 2006 | Related to ST47_5 and ST47_99 | Underwood et al. 2013 |
| H034700617 | ST47_3 | 47 | 1 | clin | UK | 2003 | None | Reuter et al. 2013 |
| HL01313013 | ST47_4 | 47 | 1 | clin | France | 2001 | None | ERR1341919 |
| H064160534 | ST47_5 | 47 | 1 | env | UK | 2006 | Related to ST47_2 and ST47_99 | ERR363994 |
| H043580159 | ST47_6 | 47 | 1 | clin | UK | 2004 | None | ERR363943 |
| H043580160 | ST47_7 | 47 | 1 | clin | UK | 2004 | None | ERR363959 |
| H043660021 | ST47_8 | 47 | 1 | clin | UK | 2004 | None | ERR363946 |
| H043680663 | ST47_9 | 47 | 1 | clin | UK | 2004 | None | ERR363949 |
| H043700021 | ST47_10 | 47 | 1 | clin | UK | 2004 | None | ERR363944 |
| H043790008 | ST47_11 | 47 | 1 | clin | UK | 2004 | None | ERR363945 |
| H052920051 | ST47_12 | 47 | 1 | clin | UK | 2005 | None | ERR363961 |
| H053540106 | ST47_13 | 47 | 1 | clin | UK | 2005 | None | ERR363948 |
| H063660005 | ST47_14 | 47 | 1 | clin | UK | 2006 | Related to ST47_15 and ST47_21 | ERR363904 |
| H063660006 | ST47_15 | 47 | 1 | clin | UK | 2006 | Related to ST47_14 and ST47_21 | ERR363922 |
| H063660009 | ST47_16 | 47 | 1 | clin | UK | 2006 | None | ERR363911 |
| H063680006 | ST47_17 | 47 | 1 | clin | UK | 2006 | Related to ST47_18 | ERR363918 |
| H063680007 | ST47_18 | 47 | 1 | clin | UK | 2006 | Related to ST47_17 | ERR363913 |
| H063740003 | ST47_19 | 47 | 1 | clin | UK | 2006 | None | ERR363929 |
| H063740018 | ST47_20 | 47 | 1 | clin | UK | 2006 | None | ERR363906 |
| H063760006 | ST47_21 | 47 | 1 | clin | UK | 2006 | Related to ST47_14 and ST47_15 | ERR363915 |
| H063780007 | ST47_22 | 47 | 1 | clin | UK | 2006 | Related to ST47_23 | ERR363934 |
| H063780008 | ST47_23 | 47 | 1 | clin | UK | 2006 | Related to ST47_22 | ERR363916 |
| H063860003 | ST47_24 | 47 | 1 | clin | UK | 2006 | None | ERR363930 |
| H063960001 | ST47_25 | 47 | 1 | clin | UK | 2006 | None | ERR363928 |
| LC5759 | ST47_26 | 47 | 1 | clin | Unknown (TA) | 2000 | None | ERR363995 |
| H070420013 | ST47_27 | 47 | 1 | clin | UK | 2007 | None | ERR363968 |
| LC5822 | ST47_28 | 47 | 1 | clin | UK | 2001 | None | ERR363996 |
| H040260015 | ST47_29 | 47 | 1 | clin | UK | 2004 | None | ERR363903 |
| H055140095 | ST47_30 | 47 | 1 | clin | UK | 2006 | None | ERR363947 |
| H060780053 | ST47_31 | 47 | 1 | clin | UK | 2006 | None | ERR363907 |
| H061120064 | ST47_32 | 47 | 1 | clin | UK | 2006 | None | ERR363914 |
| H062840608 | ST47_33 | 47 | 1 | clin | UK | 2006 | None | ERR363917 |
| H062940111 | ST47_34 | 47 | 1 | clin | UK | 2006 | None | ERR363919 |
| H064320006 | ST47_35 | 47 | 1 | clin | UK | 2006 | None | ERR363923 |
| H064280005 | ST47_36 | 47 | 1 | clin | UK | 2006 | None | ERR363924 |
| H064380002 | ST47_37 | 47 | 1 | clin | UK | 2006 | None | ERR363926 |
| H064380001 | ST47_38 | 47 | 1 | clin | UK | 2006 | None | ERR363921 |
| H064560527 | ST47_39 | 47 | 1 | clin | UK | 2006 | None | ERR363925 |
| H064660638 | ST47_40 | 47 | 1 | clin | UK | 2006 | None | ERR363964 |
| H070160015 | ST47_41 | 47 | 1 | clin | UK | 2007 | None | ERR363970 |
| H071120010 | ST47_42 | 47 | 1 | clin | UK | 2007 | None | ERR363931 |
| H071360036 | ST47_43 | 47 | 1 | clin | UK | 2007 | None | ERR363908 |
| H072740002 | ST47_44 | 47 | 1 | clin | UK | 2007 | None | ERR363935 |
| H073000045 | ST47_45 | 47 | 1 | clin | UK | 2007 | None | ERR363932 |
| H073380007 | ST47_46 | 47 | 1 | clin | UK | 2007 | None | ERR363940 |
| H073600182 | ST47_47 | 47 | 1 | clin | UK | 2007 | None | ERR363976 |
| H073640185 | ST47_48 | 47 | 1 | clin | UK | 2007 | None | ERR363933 |
| H074960018 | ST47_49 | 47 | 1 | clin | UK | 2008 | None | ERR363920 |
| H080780059 | ST47_50 | 47 | 1 | clin | UK | 2008 | None | ERR363910 |
| H053840008 | ST47_51 | 47 | 1 | clin | UK | 2004 | None | ERR363954 |
| H072520002 | ST47_52 | 47 | 1 | clin | UK | 2007 | None | ERR363927 |
| H081340222 | ST47_53 | 47 | 1 | clin | UK | 2007 | None | ERR363909 |
| H082520613 | ST47_54 | 47 | 1 | clin | UK | 2008 | None | ERR363912 |
| H083120262 | ST47_55 | 47 | 1 | clin | UK | 2008 | None | ERR363941 |
| H083620580 | ST47_56 | 47 | 1 | clin | UK | 2008 | None | ERR363936 |
| H083960064 | ST47_57 | 47 | 1 | clin | UK | 2008 | None | ERR363937 |
| H084620118 | ST47_58 | 47 | 1 | clin | UK | 2008 | None | ERR363939 |
| H090140214 | ST47_59 | 47 | 1 | clin | UK | 2009 | None | ERR363963 |
| H090440226 | ST47_60 | 47 | 1 | clin | UK | 2009 | None | ERR363966 |
| H040960441 | ST47_61 | 47 | 1 | clin | UK | 2004 | None | ERR363953 |
| H041120007 | ST47_62 | 47 | 1 | clin | UK | 2004 | None | ERR363942 |
| H093480403 | ST47_63 | 47 | 1 | clin | Unknown (TA) | 2009 | None | ERR363973 |
| H094340202 | ST47_64 | 47 | 1 | clin | UK | 2009 | None | ERR363971 |
| H095060125 | ST47_65 | 47 | 1 | clin | UK | 2009 | None | ERR363972 |
| H100140151 | ST47_66 | 47 | 1 | clin | UK | 2010 | None | ERR363965 |
| H100660110 | ST47_67 | 47 | 1 | clin | UK | 2010 | None | ERR363962 |
| H100700025 | ST47_68 | 47 | 1 | clin | UK | 2010 | None | ERR363958 |
| H103140121 | ST47_69 | 47 | 1 | clin | UK | 2010 | None | ERR363967 |
| H103620160 | ST47_70 | 47 | 1 | clin | UK | 2010 | None | ERR363950 |
| H103660126 | ST47_71 | 47 | 1 | clin | UK | 2010 | None | ERR363974 |
| H103660121 | ST47_72 | 47 | 1 | clin | UK | 2010 | None | ERR363956 |
| H104420240 | ST47_73 | 47 | 1 | clin | UK | 2010 | None | ERR363957 |
| H110480273 | ST47_74 | 47 | 1 | clin | UK | 2011 | None | ERR363969 |
| H112320437 | ST47_75 | 47 | 1 | clin | UK | 2011 | None | ERR363951 |
| H112080616 | ST47_76 | 47 | 1 | clin | UK | 2011 | None | ERR363952 |
| H112380374 | ST47_77 | 47 | 1 | clin | UK | 2011 | None | ERR363960 |
| H120160499 | ST47_78 | 47 | 1 | clin | UK | 2012 | None | ERR363985 |
| H120200371 | ST47_79 | 47 | 1 | clin | UK | 2012 | None | ERR363984 |
| H105140391 | ST47_80 | 47 | 1 | clin | UK | 2010 | None | ERR363993 |
| H121040204 | ST47_81 | 47 | 1 | clin | UK | 2012 | None | ERR363982 |
| H121420445 | ST47_82 | 47 | 1 | clin | UK | 2012 | None | ERR363983 |
| H102240357 | ST47_83 | 47 | 1 | clin | UK | 2010 | None | ERR363955 |
| H122500497 | ST47_84 | 47 | 1 | clin | UK | 2012 | None | ERR363981 |
| H122820408 | ST47_85 | 47 | 1 | clin | Unknown (TA) | 2012 | None | ERR363980 |
| H123620597 | ST47_86 | 47 | 1 | clin | UK | 2012 | None | ERR363979 |
| H123840629 | ST47_87 | 47 | 1 | clin | UK | 2012 | None | ERR363978 |
| H123940534 | ST47_88 | 47 | 1 | clin | UK | 2012 | None | ERR363975 |
| H124920387 | ST47_89 | 47 | 1 | clin | UK | 2012 | None | ERR363991 |
| H131340777 | ST47_90 | 47 | 1 | clin | UK | 2013 | Related to ST47_92, ST47_93 and ST47_94 | ERR363990 |
| H131460248 | ST47_91 | 47 | 1 | clin | UK | 2013 | None | ERR363987 |
| H131480353 | ST47_92 | 47 | 1 | env | UK | 2013 | Related to ST47_90, ST47_93 and ST47_94 | ERR363989 |
| H131480354 | ST47_93 | 47 | 1 | env | UK | 2013 | Related to ST47_90, ST47_92 and ST47_94 | ERR363988 |
| H131840211 | ST47_94 | 47 | 1 | env | UK | 2013 | Related to ST47_90, ST47_92 and ST47_93 | ERR363986 |
| H132140863 | ST47_95 | 47 | 1 | clin | UK | 2013 | None | ERR364031 |
| EUL00031 | ST47_96 | 47 | 1 | clin | France | 1994 | None | ERR376656 |
| EUL00070 | ST47_97 | 47 | 1 | clin | UK | 1996 | None | ERR376695 |
| EUL00168 | ST47_98 | 47 | 1 | clin | UK | 2005 | None | ERR352161 |
| EUL00170 | ST47_99 | 47 | 1 | env | UK | 2006 | Related to ST47_2 and ST47_5 | ERR376788 |
| LG12084002 | ST47_100 | 47 | 1 | clin | France | 2012 | None | ERR922533 |
| LG12034018 | ST47_101 | 47 | 1 | clin | France | 2012 | None | ERR1193351 |
| LG11463009 | ST47_102 | 47 | 1 | clin | France | 2011 | None | ERR922534 |
| LG11415002 | ST47_103 | 47 | 1 | clin | France | 2011 | None | ERR922535 |
| LG11403003 | ST47_104 | 47 | 1 | clin | France | 2011 | None | ERR922536 |
| LG10425016 | ST47_105 | 47 | 1 | clin | France | 2010 | None | ERR922537 |
| LG10397001 | ST47_106 | 47 | 1 | clin | France | 2010 | None | ERR922538 |
| LG09534017 | ST47_107 | 47 | 1 | clin | France | 2009 | None | ERR922539 |
| LG09471012 | ST47_108 | 47 | 1 | clin | France | 2009 | None | ERR922540 |
| LG08394013 | ST47_109 | 47 | 1 | clin | France | 2008 | None | ERR922541 |
| LG08251002 | ST47_110 | 47 | 1 | clin | France | 2008 | None | ERR922542 |
| HL07512016 | ST47_111 | 47 | 1 | clin | France | 2007 | None | ERR922543 |
| HL07055011 | ST47_112 | 47 | 1 | clin | France | 2007 | None | ERR922544 |
| HL06353025 | ST47_113 | 47 | 1 | clin | France | 2006 | None | ERR922545 |
| HL05383032 | ST47_114 | 47 | 1 | clin | France | 2005 | None | ERR922546 |
| HL05375017 | ST47_115 | 47 | 1 | clin | France | 2005 | None | ERR922547 |
| HL04411050 | ST47_116 | 47 | 1 | env | France | 2004 | None | ERR922548 |
| HL04284070 | ST47_117 | 47 | 1 | clin | France | 2004 | None | ERR922549 |
| HL04075055 | ST47_118 | 47 | 1 | clin | France | 2004 | None | ERR922550 |
| HL03503011 | ST47_119 | 47 | 1 | clin | France | 2003 | None | ERR922551 |
| HL03443027 | ST47_120 | 47 | 1 | clin | France | 2003 | None | ERR922552 |
| HL02392002 | ST47_121 | 47 | 1 | clin | France | 2002 | None | ERR922553 |
| HL02274033 | ST47_122 | 47 | 1 | clin | France | 2002 | None | ERR922554 |
| H043540106 | ST62_2 | 62 | 1 | clin | Unknown (TA) | 2004 | None | ERR363997 |
| H044120014 | ST62_3 | 62 | 1 | clin | Bulgaria | 2004 | None | ERR363999 |
| H052780022 | ST62_4 | 62 | 1 | clin | UK | 2005 | None | ERR363998 |
| H054280040 | ST62_5 | 62 | 1 | clin | UK | 2005 | None | ERR364028 |
| H063680003 | ST62_6 | 62 | 1 | clin | UK | 2006 | None | ERR364002 |
| H063840008 | ST62_7 | 62 | 1 | clin | UK | 2006 | None | ERR364001 |
| H073660582 | ST62_8 | 62 | 1 | clin | UK | 2007 | None | ERR364008 |
| LC5804 | ST62_9 | 62 | 1 | clin | UK | 2000 | None | ERR364029 |
| H063760005 | ST62_10 | 62 | 1 | clin | UK | 2006 | None | ERR364000 |
| H064240003 | ST62_11 | 62 | 1 | clin | UK | 2006 | None | ERR364005 |
| H065040012 | ST62_12 | 62 | 1 | clin | UK | 2007 | None | ERR364012 |
| H070140635 | ST62_13 | 62 | 1 | clin | UK | 2007 | None | ERR364011 |
| H073020039 | ST62_14 | 62 | 1 | clin | UK | 2007 | None | ERR364022 |
| H073320399 | ST62_15 | 62 | 1 | clin | UK | 2007 | None | ERR364010 |
| H073440003 | ST62_16 | 62 | 1 | clin | UK | 2007 | None | ERR364009 |
| LC6009 | ST62_17 | 62 | 1 | clin | Unknown (TA) | 2001 | None | ERR364030 |
| H083140015 | ST62_18 | 62 | 1 | clin | UK | 2008 | None | ERR364007 |
| H064180019 | ST62_19 | 62 | 1 | env | UK | 2006 | Related to ST62_1 | ERR364004 |
| H093400182 | ST62_20 | 62 | 1 | clin | UK | 2009 | None | ERR364006 |
| H094760070 | ST62_21 | 62 | 1 | clin | UK | 2009 | None | ERR364003 |
| H094800237 | ST62_22 | 62 | 1 | clin | UK | 2009 | None | ERR364020 |
| H110480715 | ST62_23 | 62 | 1 | clin | UK | 2011 | None | ERR364018 |
| H112840293 | ST62_24 | 62 | 1 | clin | UK | 2011 | None | ERR364017 |
| H114100406 | ST62_25 | 62 | 1 | clin | Greece | 2011 | None | ERR364016 |
| H120240362 | ST62_26 | 62 | 1 | clin | UK | 2012 | None | ERR364025 |
| H104640262 | ST62_27 | 62 | 1 | clin | Unknown (TA) | 2010 | None | ERR364019 |
| H123140428 | ST62_28 | 62 | 1 | env | UK | 2012 | None | ERR364015 |
| H123460520 | ST62_29 | 62 | 1 | clin | UK | 2012 | None | ERR364014 |
| H124360642 | ST62_30 | 62 | 1 | clin | UK | 2012 | None | ERR364013 |
| EUL00054 | ST62_31 | 62 | 1 | clin | Spain | 1994 | Related to ST62_32 | ERR332140 |
| EUL00057 | ST62_32 | 62 | 1 | env | Spain | 1995 | Related to ST62_31 | ERR332143 |
| EUL00071 | ST62_33 | 62 | 1 | clin | UK | 1996 | Related to ST62_34 and ST62_35 | ERR332157 |
| EUL00076 | ST62_34 | 62 | 1 | clin | UK | 1996 | Related to ST62_33 and ST62_35 | ERR332162 |
| EUL00077 | ST62_35 | 62 | 1 | clin | UK | 1996 | Related to ST62_33 and ST62_34 | ERR332163 |
| EUL00007 |  | 18 | 1 | clin | Switzerland | 1992 | None | ERR376632 |
| LG12482019 |  | 146 | 1 | clin | France | 2012 | None | ERR923430 |

ST, sequence type

Sg, serogroup

env, environmental

clin, clinical

TA, travel-associated

(1) in the “ST” column refers to isolates nested between ST1 isolates in a phylogenetic tree
