# Supplementary material for: Multiple major disease-associated clones of Legionella pneumophila have emerged recently and independently
Source: Genome Res. 2016 Nov;26(11):1555–64. doi: 10.1101/gr.209536.116 (PMC5088597; doi:10.1101/gr.209536.116)
Supplement: Supplemental Material [file supp_gr.209536.116_Supplemental_Table_S3.docx]

Table S3. The reference genomes used and the number of SNPs identified within each of the five disease-associated lineages before recombination is removed.

| **ST** | **Number of isolates** | **Mapping reference** | **Total number of SNPs** | **Maximum number of pairwise SNP differences** | **Percentage SNPs that are homoplasic** |
| --- | --- | --- | --- | --- | --- |
| ST1 (+ ST1-derived isolates) | 71 | Paris  (complete genome) | 48,655 | 15,227 | 35·9 |
| ST23 | 37 | EUL00011/ST23_3 (*de novo* assembly) | 26,945 | 12,964 | 11·8 |
| ST37 | 72 | EUL00132/ST37_69 (*de novo* assembly) | 14,829 | 13,776 | 0·06 |
| ST47 | 122 | Lorraine  (complete genome) | 186 | 19 | 0 |
| ST62 | 35 | H043540106/ST62_2 (*de novo* assembly) | 33,200 | 12,842 | 36·4 |
