# Supplementary material for: Multiple major disease-associated clones of Legionella pneumophila have emerged recently and independently
Source: Genome Res. 2016 Nov;26(11):1555–64. doi: 10.1101/gr.209536.116 (PMC5088597; doi:10.1101/gr.209536.116)
Supplement: Supplemental Material [file supp_gr.209536.116_Supplemental_Table_S4.docx]

Table S4. Recombined regions in STs 1, 23, 37 and 62.

| **Lineage** | **Start** | **End** | **Affected taxa** | **# of SNPs introduced by event** |
| --- | --- | --- | --- | --- |
| ST1 | 720073 | 720113 | ST1_14 | 6 |
| ST1 | 914955 | 967396 | ST390_1 | 625 |
| ST1 | 696167 | 755335 | ST10_1 | 1536 |
| ST1 | 775012 | 777299 | ST10_1 | 68 |
| ST1 | 948967 | 952146 | ST10_1 | 21 |
| ST1 | 3376219 | 3399076 | ST1_37 | 361 |
| ST1 | 1454818 | 1470416 | ST1_37 | 250 |
| ST1 | 3360975 | 3365498 | ST1_37 | 91 |
| ST1 | 718950 | 720333 | ST1_37 | 31 |
| ST1 | 718950 | 720333 | ST1_14, ST390_1 | 27 |
| ST1 | 1994399 | 2001091 | ST1_53 | 358 |
| ST1 | 1628854 | 1634840 | ST1_53 | 100 |
| ST1 | 1154905 | 1159167 | ST1_53 | 61 |
| ST1 | 923579 | 931250 | ST1_53 | 13 |
| ST1 | 2258179 | 2309479 | ST1_14, ST390_1, ST10_1, ST1_37 | 1402 |
| ST1 | 1988947 | 2036482 | ST1_14, ST390_1, ST10_1, ST1_37 | 939 |
| ST1 | 613529 | 624131 | ST1_14, ST390_1, ST10_1, ST1_37 | 269 |
| ST1 | 1782638 | 1811993 | ST1_14, ST390_1, ST10_1, ST1_37 | 289 |
| ST1 | 713674 | 720318 | ST1_14, ST390_1, ST10_1, ST1_37 | 190 |
| ST1 | 917023 | 933108 | ST1_14, ST390_1, ST10_1, ST1_37 | 219 |
| ST1 | 568300 | 576793 | ST1_14, ST390_1, ST10_1, ST1_37 | 174 |
| ST1 | 2685258 | 2688678 | ST1_14, ST390_1, ST10_1, ST1_37 | 124 |
| ST1 | 1304731 | 1326450 | ST1_14, ST390_1, ST10_1, ST1_37 | 155 |
| ST1 | 2067801 | 2071190 | ST1_14, ST390_1, ST10_1, ST1_37 | 60 |
| ST1 | 789607 | 797972 | ST1_14, ST390_1, ST10_1, ST1_37 | 41 |
| ST1 | 1135667 | 1142949 | ST1_14, ST390_1, ST10_1, ST1_37 | 29 |
| ST1 | 1838009 | 1839319 | ST1_14, ST390_1, ST10_1, ST1_37 | 19 |
| ST1 | 1068888 | 1075051 | ST1_14, ST390_1, ST10_1, ST1_37 | 16 |
| ST1 | 1250656 | 1254075 | ST1_14, ST390_1, ST10_1, ST1_37 | 8 |
| ST1 | 1994399 | 2001091 | ST1_45 | 358 |
| ST1 | 923579 | 931250 | ST1_45 | 13 |
| ST1 | 923579 | 931250 | ST1_29 | 13 |
| ST1 | 899423 | 931305 | ST7_2, ST7_3 | 425 |
| ST1 | 190429 | 190457 | ST7_2, ST7_3 | 12 |
| ST1 | 917120 | 932822 | ST1_56 | 210 |
| ST1 | 425877 | 432715 | ST1_56 | 114 |
| ST1 | 923579 | 931250 | ST1_44 | 13 |
| ST1 | 23703 | 41752 | ST1_29, ST7_2, ST7_3, ST1_56, ST1_44 | 254 |
| ST1 | 54158 | 60859 | ST1_29, ST7_2, ST7_3, ST1_56, ST1_44 | 199 |
| ST1 | 622300 | 628159 | ST1_29, ST7_2, ST7_3, ST1_56, ST1_44 | 120 |
| ST1 | 1818963 | 1822135 | ST1_29, ST7_2, ST7_3, ST1_56, ST1_44 | 58 |
| ST1 | 1994515 | 2001118 | ST1_29, ST7_2, ST7_3, ST1_56, ST1_44 | 49 |
| ST1 | 915120 | 955337 | ST5_2, ST5_3, ST5_1 | 1159 |
| ST1 | 6516 | 34195 | ST5_2, ST5_3, ST5_1 | 194 |
| ST1 | 2895018 | 2895040 | ST5_2, ST5_3, ST5_1 | 9 |
| ST1 | 404935 | 406960 | ST5_2, ST5_3, ST5_1 | 9 |
| ST1 | 136636 | 150768 | ST5_2, ST5_3, ST5_1 | 19 |
| ST1 | 607809 | 619627 | ST5_2, ST5_3, ST5_1 | 10 |
| ST1 | 642420 | 643590 | ST5_2, ST5_3, ST5_1 | 6 |
| ST1 | 3310617 | 3319330 | ST1_8 | 224 |
| ST1 | 2994178 | 3006241 | ST1_8 | 118 |
| ST1 | 3162890 | 3194513 | ST1_8 | 128 |
| ST1 | 2954911 | 2963935 | ST1_8 | 86 |
| ST1 | 3091916 | 3106059 | ST1_8 | 92 |
| ST1 | 3228756 | 3249903 | ST1_8 | 89 |
| ST1 | 3422165 | 3423998 | ST1_8 | 51 |
| ST1 | 2520359 | 2529673 | ST1_8 | 62 |
| ST1 | 3149042 | 3155746 | ST1_8 | 21 |
| ST1 | 3164330 | 3184696 | ST1_57 | 424 |
| ST1 | 1997105 | 2040992 | ST1_57 | 431 |
| ST1 | 795565 | 800955 | ST1_57 | 99 |
| ST1 | 855034 | 857001 | ST1_57 | 43 |
| ST1 | 831990 | 833348 | ST1_57 | 7 |
| ST1 | 1997091 | 2001919 | ST1_43, ST1_38, ST1_35, Paris, ST1_32, ST1_59, ST1_20, ST1_6, ST1_9 | 234 |
| ST1 | 190429 | 190457 | ST1_11, ST1_33, ST1_42, ST1_40, ST1_41, ST1_34 | 12 |
| ST1 | 1992558 | 1997072 | ST1_57, ST1_43, ST1_38, ST1_35, Paris, ST1_32, ST1_59, ST1_20, ST1_6, ST1_9 | 28 |
| ST1 | 392896 | 420410 | ST1_39, ST1_11, ST1_33, ST1_42, ST1_40, ST1_41, ST1_34 | 1025 |
| ST1 | 1981678 | 2013225 | ST1_39, ST1_11, ST1_33, ST1_42, ST1_40, ST1_41, ST1_34 | 497 |
| ST1 | 1994399 | 2002152 | ST1_16 | 261 |
| ST1 | 1981424 | 2035681 | ST1_48 | 1003 |
| ST1 | 2365816 | 2377087 | ST1_48 | 397 |
| ST1 | 2294421 | 2307736 | ST1_48 | 245 |
| ST1 | 1911568 | 1921849 | ST1_48 | 234 |
| ST1 | 2956323 | 2965649 | ST1_48 | 53 |
| ST1 | 2884279 | 2907327 | ST1_48 | 29 |
| ST1 | 1994399 | 2002152 | ST1_7 | 231 |
| ST1 | 1997120 | 2001806 | ST1_48, ST1_7 | 30 |
| ST1 | 1682116 | 1811526 | ST1_36 | 1962 |
| ST1 | 1078977 | 1114651 | ST1_36 | 879 |
| ST1 | 1814012 | 1852771 | ST1_36 | 604 |
| ST1 | 1040800 | 1070969 | ST1_36 | 539 |
| ST1 | 2895221 | 2902683 | ST1_36 | 196 |
| ST1 | 2526748 | 2530864 | ST1_36 | 86 |
| ST1 | 922796 | 929825 | ST1_36 | 84 |
| ST1 | 1653633 | 1658370 | ST1_36 | 57 |
| ST1 | 529824 | 535837 | ST1_36 | 29 |
| ST1 | 386193 | 396154 | ST1_36 | 10 |
| ST1 | 369282 | 373337 | ST1_36 | 7 |
| ST1 | 299130 | 309007 | ST1_46, ST1_27, ST1_47, ST1_10, ST1_55, ST1_26 | 338 |
| ST1 | 2422757 | 2427342 | ST1_46, ST1_27, ST1_47, ST1_10, ST1_55, ST1_26 | 124 |
| ST1 | 915641 | 928057 | ST1_46, ST1_27, ST1_47, ST1_10, ST1_55, ST1_26 | 111 |
| ST1 | 858081 | 933217 | ST7_1 | 1232 |
| ST1 | 1983181 | 1999248 | ST7_1 | 278 |
| ST1 | 791380 | 807526 | ST7_1 | 239 |
| ST1 | 2484447 | 2490279 | ST7_1 | 33 |
| ST1 | 1999688 | 2004941 | ST7_1 | 7 |
| ST1 | 1981457 | 2018046 | ST1_36, ST1_46, ST1_27, ST1_47, ST1_10, ST1_55, ST1_26 | 749 |
| ST1 | 917563 | 929335 | ST1_36, ST1_46, ST1_27, ST1_47, ST1_10, ST1_55, ST1_26 | 332 |
| ST1 | 843689 | 861966 | ST1_36, ST1_46, ST1_27, ST1_47, ST1_10, ST1_55, ST1_26 | 347 |
| ST1 | 877964 | 891092 | ST1_36, ST1_46, ST1_27, ST1_47, ST1_10, ST1_55, ST1_26 | 224 |
| ST1 | 2091402 | 2104212 | ST1_36, ST1_46, ST1_27, ST1_47, ST1_10, ST1_55, ST1_26 | 214 |
| ST1 | 1997367 | 1997745 | ST1_16, ST1_57, ST1_43, ST1_38, ST1_35, Paris, ST1_32, ST1_59, ST1_20, ST1_6, ST1_9, ST1_39, ST1_11, ST1_33, ST1_42, ST1_40, ST1_41, ST1_34, ST1_48, ST1_7 | 5 |
| ST1 | 2860553 | 2909359 | ST1_28 | 1663 |
| ST1 | 2966569 | 2988686 | ST1_28 | 934 |
| ST1 | 271865 | 291538 | ST1_28 | 824 |
| ST1 | 351098 | 365245 | ST1_28 | 477 |
| ST1 | 591359 | 610326 | ST1_28 | 475 |
| ST1 | 3387578 | 3396011 | ST1_28 | 366 |
| ST1 | 2918024 | 2927687 | ST1_28 | 274 |
| ST1 | 254646 | 262413 | ST1_28 | 253 |
| ST1 | 2939203 | 2945769 | ST1_28 | 170 |
| ST1 | 95817 | 105285 | ST1_28 | 168 |
| ST1 | 1465283 | 1470416 | ST1_28 | 135 |
| ST1 | 787084 | 789218 | ST1_28 | 25 |
| ST1 | 28886 | 30371 | ST1_28 | 11 |
| ST1 | 3380081 | 3395276 | ST1_13 | 473 |
| ST1 | 1981994 | 2008857 | ST1_12 | 423 |
| ST1 | 256074 | 264516 | ST1_28, ST1_13, ST72_1 | 188 |
| ST1 | 1994399 | 2015013 | ST1_28, ST1_13, ST72_1 | 141 |
| ST1 | 3393726 | 3395209 | ST1_28, ST1_13, ST72_1 | 73 |
| ST1 | 1997012 | 2010965 | ST1_12, ST1_28, ST1_13, ST72_1 | 101 |
| ST1 | 190502 | 190530 | ST1_17 | 11 |
| ST1 | 2959733 | 2968259 | ST1_50, ST1_54, ST1_49 | 460 |
| ST1 | 1164139 | 1194956 | ST1_50, ST1_54, ST1_49 | 411 |
| ST1 | 1992861 | 2004662 | ST1_50, ST1_54, ST1_49 | 225 |
| ST1 | 926478 | 932256 | ST1_50, ST1_54, ST1_49 | 143 |
| ST1 | 648716 | 650067 | ST1_50, ST1_54, ST1_49 | 47 |
| ST1 | 192080 | 192110 | ST1_50, ST1_54, ST1_49 | 12 |
| ST1 | 1981331 | 2027320 | ST1_30 | 656 |
| ST1 | 2074251 | 2094482 | ST1_30 | 187 |
| ST1 | 1921849 | 1928683 | ST1_30 | 123 |
| ST1 | 2144221 | 2147242 | ST1_30 | 62 |
| ST1 | 1994399 | 2002152 | ST1_31 | 251 |
| ST1 | 1994399 | 2002152 | ST1_18, ST1_17 | 251 |
| ST1 | 1994399 | 2001919 | ST1_3, ST1_15 | 250 |
| ST1 | 3061410 | 3069294 | ST1_3, ST1_15 | 64 |
| ST1 | 2319666 | 2324386 | ST1_16, ST1_57, ST1_43, ST1_38, ST1_35, Paris, ST1_32, ST1_59, ST1_20, ST1_6, ST1_9, ST1_39, ST1_11, ST1_33, ST1_42, ST1_40, ST1_41, ST1_34, ST1_48, ST1_7, ST7_1, ST1_36, ST1_46, ST1_27, ST1_47, ST1_10, ST1_55, ST1_26, ST1_12, ST1_28, ST1_13, ST72_1 | 97 |
| ST1 | 2014953 | 2018845 | ST1_16, ST1_57, ST1_43, ST1_38, ST1_35, Paris, ST1_32, ST1_59, ST1_20, ST1_6, ST1_9, ST1_39, ST1_11, ST1_33, ST1_42, ST1_40, ST1_41, ST1_34, ST1_48, ST1_7, ST7_1, ST1_36, ST1_46, ST1_27, ST1_47, ST1_10, ST1_55, ST1_26, ST1_12, ST1_28, ST1_13, ST72_1 | 69 |
| ST1 | 1997300 | 2001806 | ST1_16, ST1_57, ST1_43, ST1_38, ST1_35, Paris, ST1_32, ST1_59, ST1_20, ST1_6, ST1_9, ST1_39, ST1_11, ST1_33, ST1_42, ST1_40, ST1_41, ST1_34, ST1_48, ST1_7, ST7_1, ST1_36, ST1_46, ST1_27, ST1_47, ST1_10, ST1_55, ST1_26, ST1_12, ST1_28, ST1_13, ST72_1 | 7 |
| ST1 | 1998845 | 1998859 | ST1_18, ST1_17, ST1_50, ST1_54, ST1_49, ST1_30, ST1_31, ST1_3, ST1_15 | 8 |
| ST1 | 2852226 | 2943974 | ST5_2, ST5_3, ST5_1, ST1_8 | 1074 |
| ST1 | 2125861 | 2148867 | ST5_2, ST5_3, ST5_1, ST1_8 | 524 |
| ST1 | 2964210 | 2994118 | ST5_2, ST5_3, ST5_1, ST1_8 | 532 |
| ST1 | 401143 | 432534 | ST5_2, ST5_3, ST5_1, ST1_8 | 407 |
| ST1 | 1315589 | 1341685 | ST5_2, ST5_3, ST5_1, ST1_8 | 271 |
| ST1 | 1986118 | 2005626 | ST5_2, ST5_3, ST5_1, ST1_8 | 244 |
| ST1 | 2795306 | 2817256 | ST5_2, ST5_3, ST5_1, ST1_8 | 233 |
| ST1 | 1843392 | 1853272 | ST5_2, ST5_3, ST5_1, ST1_8 | 119 |
| ST1 | 3017846 | 3052614 | ST5_2, ST5_3, ST5_1, ST1_8 | 177 |
| ST1 | 2428004 | 2439942 | ST5_2, ST5_3, ST5_1, ST1_8 | 116 |
| ST1 | 1509886 | 1513332 | ST5_2, ST5_3, ST5_1, ST1_8 | 58 |
| ST1 | 1871371 | 1880112 | ST5_2, ST5_3, ST5_1, ST1_8 | 48 |
| ST1 | 2945415 | 2950814 | ST5_2, ST5_3, ST5_1, ST1_8 | 40 |
| ST1 | 1972681 | 1976119 | ST5_2, ST5_3, ST5_1, ST1_8 | 32 |
| ST1 | 1589801 | 1595969 | ST5_2, ST5_3, ST5_1, ST1_8 | 21 |
| ST1 | 437938 | 443814 | ST5_2, ST5_3, ST5_1, ST1_8 | 18 |
| ST1 | 2617972 | 2620919 | ST5_2, ST5_3, ST5_1, ST1_8 | 13 |
| ST1 | 2518655 | 2518982 | ST5_2, ST5_3, ST5_1, ST1_8 | 8 |
| ST1 | 68053 | 76221 | ST1_16, ST1_57, ST1_43, ST1_38, ST1_35, Paris, ST1_32, ST1_59, ST1_20, ST1_6, ST1_9, ST1_39, ST1_11, ST1_33, ST1_42, ST1_40, ST1_41, ST1_34, ST1_48, ST1_7, ST7_1, ST1_36, ST1_46, ST1_27, ST1_47, ST1_10, ST1_55, ST1_26, ST1_12, ST1_28, ST1_13, ST72_1, ST1_18, ST1_17, ST1_50, ST1_54, ST1_49, ST1_30, ST1_31, ST1_3, ST1_15 | 160 |
| ST1 | 1994067 | 1999248 | ST1_16, ST1_57, ST1_43, ST1_38, ST1_35, Paris, ST1_32, ST1_59, ST1_20, ST1_6, ST1_9, ST1_39, ST1_11, ST1_33, ST1_42, ST1_40, ST1_41, ST1_34, ST1_48, ST1_7, ST7_1, ST1_36, ST1_46, ST1_27, ST1_47, ST1_10, ST1_55, ST1_26, ST1_12, ST1_28, ST1_13, ST72_1, ST1_18, ST1_17, ST1_50, ST1_54, ST1_49, ST1_30, ST1_31, ST1_3, ST1_15 | 95 |
| ST1 | 838377 | 919389 | ST152_1 | 1362 |
| ST1 | 3049179 | 3101539 | ST152_1 | 975 |
| ST1 | 137388 | 167272 | ST152_1 | 619 |
| ST1 | 392607 | 414711 | ST152_1 | 345 |
| ST1 | 351708 | 382225 | ST152_1 | 147 |
| ST1 | 23516 | 27518 | ST152_1 | 74 |
| ST1 | 1988831 | 2004941 | ST152_1 | 54 |
| ST1 | 1915110 | 1916841 | ST152_1 | 32 |
| ST1 | 421687 | 432143 | ST152_1 | 33 |
| ST1 | 68804 | 69392 | ST152_1 | 7 |
| ST1 | 110845 | 113854 | ST152_1 | 9 |
| ST1 | 699197 | 699401 | ST152_1 | 5 |
| ST1 | 383284 | 391290 | ST152_1 | 8 |
| ST1 | 440235 | 445176 | ST152_1 | 6 |
| ST1 | 1060915 | 1114436 | ST1_58 | 860 |
| ST1 | 2392732 | 2421895 | ST1_58 | 615 |
| ST1 | 993110 | 1038854 | ST1_58 | 638 |
| ST1 | 298539 | 314989 | ST1_58 | 460 |
| ST1 | 3363756 | 3413651 | ST1_58 | 493 |
| ST1 | 1990080 | 2017240 | ST1_58 | 409 |
| ST1 | 1802571 | 1857618 | ST1_58 | 452 |
| ST1 | 2530045 | 2549526 | ST1_58 | 317 |
| ST1 | 2280942 | 2293075 | ST1_58 | 188 |
| ST1 | 832911 | 837701 | ST1_58 | 45 |
| ST1 | 2220837 | 2224037 | ST1_58 | 30 |
| ST1 | 3457154 | 3457924 | ST1_58 | 20 |
| ST1 | 1982357 | 1983159 | ST1_58 | 12 |
| ST1 | 2661627 | 2724645 | ST1_22, ST1_19, ST1_21, ST1_23, ST1_25, ST1_5, ST1_24, ST1_4 | 4005 |
| ST1 | 1160175 | 1203349 | ST1_22, ST1_19, ST1_21, ST1_23, ST1_25, ST1_5, ST1_24, ST1_4 | 2130 |
| ST1 | 182787 | 219994 | ST1_22, ST1_19, ST1_21, ST1_23, ST1_25, ST1_5, ST1_24, ST1_4 | 1810 |
| ST1 | 1981851 | 2025625 | ST1_22, ST1_19, ST1_21, ST1_23, ST1_25, ST1_5, ST1_24, ST1_4 | 482 |
| ST1 | 936298 | 936831 | ST1_22, ST1_19, ST1_21, ST1_23, ST1_25, ST1_5, ST1_24, ST1_4 | 65 |
| ST1 | 1454845 | 1458138 | ST1_22, ST1_19, ST1_21, ST1_23, ST1_25, ST1_5, ST1_24, ST1_4 | 47 |
| ST1 | 68053 | 76221 | ST152_1, ST1_58 | 153 |
| ST1 | 1205044 | 1211176 | ST152_1, ST1_58 | 25 |
| ST1 | 1992350 | 1993125 | ST152_1, ST1_58 | 5 |
| ST1 | 1994633 | 2000140 | ST5_2, ST5_3, ST5_1, ST1_8, ST1_16, ST1_57, ST1_43, ST1_38, ST1_35, Paris, ST1_32, ST1_59, ST1_20, ST1_6, ST1_9, ST1_39, ST1_11, ST1_33, ST1_42, ST1_40, ST1_41, ST1_34, ST1_48, ST1_7, ST7_1, ST1_36, ST1_46, ST1_27, ST1_47, ST1_10, ST1_55, ST1_26, ST1_12, ST1_28, ST1_13, ST72_1, ST1_18, ST1_17, ST1_50, ST1_54, ST1_49, ST1_30, ST1_31, ST1_3, ST1_15 | 50 |
| ST1 | 923474 | 933108 | ST5_2, ST5_3, ST5_1, ST1_8, ST1_16, ST1_57, ST1_43, ST1_38, ST1_35, Paris, ST1_32, ST1_59, ST1_20, ST1_6, ST1_9, ST1_39, ST1_11, ST1_33, ST1_42, ST1_40, ST1_41, ST1_34, ST1_48, ST1_7, ST7_1, ST1_36, ST1_46, ST1_27, ST1_47, ST1_10, ST1_55, ST1_26, ST1_12, ST1_28, ST1_13, ST72_1, ST1_18, ST1_17, ST1_50, ST1_54, ST1_49, ST1_30, ST1_31, ST1_3, ST1_15 | 51 |
| ST1 | 2055666 | 2061785 | ST5_2, ST5_3, ST5_1, ST1_8, ST1_16, ST1_57, ST1_43, ST1_38, ST1_35, Paris, ST1_32, ST1_59, ST1_20, ST1_6, ST1_9, ST1_39, ST1_11, ST1_33, ST1_42, ST1_40, ST1_41, ST1_34, ST1_48, ST1_7, ST7_1, ST1_36, ST1_46, ST1_27, ST1_47, ST1_10, ST1_55, ST1_26, ST1_12, ST1_28, ST1_13, ST72_1, ST1_18, ST1_17, ST1_50, ST1_54, ST1_49, ST1_30, ST1_31, ST1_3, ST1_15 | 46 |
| ST1 | 2072723 | 2076994 | ST5_2, ST5_3, ST5_1, ST1_8, ST1_16, ST1_57, ST1_43, ST1_38, ST1_35, Paris, ST1_32, ST1_59, ST1_20, ST1_6, ST1_9, ST1_39, ST1_11, ST1_33, ST1_42, ST1_40, ST1_41, ST1_34, ST1_48, ST1_7, ST7_1, ST1_36, ST1_46, ST1_27, ST1_47, ST1_10, ST1_55, ST1_26, ST1_12, ST1_28, ST1_13, ST72_1, ST1_18, ST1_17, ST1_50, ST1_54, ST1_49, ST1_30, ST1_31, ST1_3, ST1_15 | 34 |
| ST1 | 2084600 | 2094628 | ST5_2, ST5_3, ST5_1, ST1_8, ST1_16, ST1_57, ST1_43, ST1_38, ST1_35, Paris, ST1_32, ST1_59, ST1_20, ST1_6, ST1_9, ST1_39, ST1_11, ST1_33, ST1_42, ST1_40, ST1_41, ST1_34, ST1_48, ST1_7, ST7_1, ST1_36, ST1_46, ST1_27, ST1_47, ST1_10, ST1_55, ST1_26, ST1_12, ST1_28, ST1_13, ST72_1, ST1_18, ST1_17, ST1_50, ST1_54, ST1_49, ST1_30, ST1_31, ST1_3, ST1_15 | 27 |
| ST1 | 467796 | 476192 | ST5_2, ST5_3, ST5_1, ST1_8, ST1_16, ST1_57, ST1_43, ST1_38, ST1_35, Paris, ST1_32, ST1_59, ST1_20, ST1_6, ST1_9, ST1_39, ST1_11, ST1_33, ST1_42, ST1_40, ST1_41, ST1_34, ST1_48, ST1_7, ST7_1, ST1_36, ST1_46, ST1_27, ST1_47, ST1_10, ST1_55, ST1_26, ST1_12, ST1_28, ST1_13, ST72_1, ST1_18, ST1_17, ST1_50, ST1_54, ST1_49, ST1_30, ST1_31, ST1_3, ST1_15 | 16 |
| ST1 | 945693 | 954162 | ST5_2, ST5_3, ST5_1, ST1_8, ST1_16, ST1_57, ST1_43, ST1_38, ST1_35, Paris, ST1_32, ST1_59, ST1_20, ST1_6, ST1_9, ST1_39, ST1_11, ST1_33, ST1_42, ST1_40, ST1_41, ST1_34, ST1_48, ST1_7, ST7_1, ST1_36, ST1_46, ST1_27, ST1_47, ST1_10, ST1_55, ST1_26, ST1_12, ST1_28, ST1_13, ST72_1, ST1_18, ST1_17, ST1_50, ST1_54, ST1_49, ST1_30, ST1_31, ST1_3, ST1_15 | 14 |
| ST1 | 2928006 | 2929589 | ST5_2, ST5_3, ST5_1, ST1_8, ST1_16, ST1_57, ST1_43, ST1_38, ST1_35, Paris, ST1_32, ST1_59, ST1_20, ST1_6, ST1_9, ST1_39, ST1_11, ST1_33, ST1_42, ST1_40, ST1_41, ST1_34, ST1_48, ST1_7, ST7_1, ST1_36, ST1_46, ST1_27, ST1_47, ST1_10, ST1_55, ST1_26, ST1_12, ST1_28, ST1_13, ST72_1, ST1_18, ST1_17, ST1_50, ST1_54, ST1_49, ST1_30, ST1_31, ST1_3, ST1_15 | 5 |
| ST1 | 1648686 | 1675289 | ST1_22, ST1_19, ST1_21, ST1_23, ST1_25, ST1_5, ST1_24, ST1_4, ST152_1, ST1_58 | 251 |
| ST1 | 923274 | 951358 | ST1_22, ST1_19, ST1_21, ST1_23, ST1_25, ST1_5, ST1_24, ST1_4, ST152_1, ST1_58 | 156 |
| ST1 | 1983181 | 1990908 | ST1_22, ST1_19, ST1_21, ST1_23, ST1_25, ST1_5, ST1_24, ST1_4, ST152_1, ST1_58 | 90 |
| ST1 | 895759 | 899123 | ST1_22, ST1_19, ST1_21, ST1_23, ST1_25, ST1_5, ST1_24, ST1_4, ST152_1, ST1_58 | 54 |
| ST1 | 2001439 | 2004941 | ST5_2, ST5_3, ST5_1, ST1_8, ST1_16, ST1_57, ST1_43, ST1_38, ST1_35, Paris, ST1_32, ST1_59, ST1_20, ST1_6, ST1_9, ST1_39, ST1_11, ST1_33, ST1_42, ST1_40, ST1_41, ST1_34, ST1_48, ST1_7, ST7_1, ST1_36, ST1_46, ST1_27, ST1_47, ST1_10, ST1_55, ST1_26, ST1_12, ST1_28, ST1_13, ST72_1, ST1_18, ST1_17, ST1_50, ST1_54, ST1_49, ST1_30, ST1_31, ST1_3, ST1_15, ST1_22, ST1_19, ST1_21, ST1_23, ST1_25, ST1_5, ST1_24, ST1_4, ST152_1, ST1_58 | 9 |
| ST1 | 854668 | 940706 | ST8_1 | 1268 |
| ST1 | 7231 | 40824 | ST8_1 | 705 |
| ST1 | 1051130 | 1085521 | ST8_1 | 661 |
| ST1 | 1110682 | 1138668 | ST8_1 | 540 |
| ST1 | 2958721 | 2970009 | ST8_1 | 377 |
| ST1 | 976443 | 990189 | ST8_1 | 197 |
| ST1 | 1793798 | 1815096 | ST8_1 | 203 |
| ST1 | 1173689 | 1174215 | ST8_1 | 35 |
| ST1 | 1991641 | 1997009 | ST8_1 | 38 |
| ST1 | 948496 | 953507 | ST8_1 | 30 |
| ST1 | 2503938 | 2533058 | ST1_45, ST1_53, ST1_14, ST390_1, ST10_1, ST1_37, ST1_29, ST7_2, ST7_3, ST1_56, ST1_44 | 129 |
| ST1 | 1992558 | 1996998 | ST1_45, ST1_53, ST1_14, ST390_1, ST10_1, ST1_37, ST1_29, ST7_2, ST7_3, ST1_56, ST1_44 | 40 |
| ST1 | 923474 | 933108 | ST1_45, ST1_53, ST1_14, ST390_1, ST10_1, ST1_37, ST1_29, ST7_2, ST7_3, ST1_56, ST1_44 | 38 |
| ST1 | 946171 | 951936 | ST1_45, ST1_53, ST1_14, ST390_1, ST10_1, ST1_37, ST1_29, ST7_2, ST7_3, ST1_56, ST1_44 | 9 |
| ST1 | 2592582 | 2599836 | ST1_45, ST1_53, ST1_14, ST390_1, ST10_1, ST1_37, ST1_29, ST7_2, ST7_3, ST1_56, ST1_44 | 8 |
| ST1 | 2552448 | 2558952 | ST1_45, ST1_53, ST1_14, ST390_1, ST10_1, ST1_37, ST1_29, ST7_2, ST7_3, ST1_56, ST1_44 | 7 |
| ST1 | 942814 | 944200 | ST1_45, ST1_53, ST1_14, ST390_1, ST10_1, ST1_37, ST1_29, ST7_2, ST7_3, ST1_56, ST1_44, ST5_2, ST5_3, ST5_1, ST1_8, ST1_16, ST1_57, ST1_43, ST1_38, ST1_35, Paris, ST1_32, ST1_59, ST1_20, ST1_6, ST1_9, ST1_39, ST1_11, ST1_33, ST1_42, ST1_40, ST1_41, ST1_34, ST1_48, ST1_7, ST7_1, ST1_36, ST1_46, ST1_27, ST1_47, ST1_10, ST1_55, ST1_26, ST1_12, ST1_28, ST1_13, ST72_1, ST1_18, ST1_17, ST1_50, ST1_54, ST1_49, ST1_30, ST1_31, ST1_3, ST1_15, ST1_22, ST1_19, ST1_21, ST1_23, ST1_25, ST1_5, ST1_24, ST1_4, ST152_1, ST1_58, ST8_1 | 126 |
| ST1 | 1443483 | 1481944 | ST1_45, ST1_53, ST1_14, ST390_1, ST10_1, ST1_37, ST1_29, ST7_2, ST7_3, ST1_56, ST1_44, ST5_2, ST5_3, ST5_1, ST1_8, ST1_16, ST1_57, ST1_43, ST1_38, ST1_35, Paris, ST1_32, ST1_59, ST1_20, ST1_6, ST1_9, ST1_39, ST1_11, ST1_33, ST1_42, ST1_40, ST1_41, ST1_34, ST1_48, ST1_7, ST7_1, ST1_36, ST1_46, ST1_27, ST1_47, ST1_10, ST1_55, ST1_26, ST1_12, ST1_28, ST1_13, ST72_1, ST1_18, ST1_17, ST1_50, ST1_54, ST1_49, ST1_30, ST1_31, ST1_3, ST1_15, ST1_22, ST1_19, ST1_21, ST1_23, ST1_25, ST1_5, ST1_24, ST1_4, ST152_1, ST1_58, ST8_1 | 172 |
| ST1 | 1490300 | 1520516 | ST1_45, ST1_53, ST1_14, ST390_1, ST10_1, ST1_37, ST1_29, ST7_2, ST7_3, ST1_56, ST1_44, ST5_2, ST5_3, ST5_1, ST1_8, ST1_16, ST1_57, ST1_43, ST1_38, ST1_35, Paris, ST1_32, ST1_59, ST1_20, ST1_6, ST1_9, ST1_39, ST1_11, ST1_33, ST1_42, ST1_40, ST1_41, ST1_34, ST1_48, ST1_7, ST7_1, ST1_36, ST1_46, ST1_27, ST1_47, ST1_10, ST1_55, ST1_26, ST1_12, ST1_28, ST1_13, ST72_1, ST1_18, ST1_17, ST1_50, ST1_54, ST1_49, ST1_30, ST1_31, ST1_3, ST1_15, ST1_22, ST1_19, ST1_21, ST1_23, ST1_25, ST1_5, ST1_24, ST1_4, ST152_1, ST1_58, ST8_1 | 132 |
| ST1 | 3299584 | 3315343 | ST1_45, ST1_53, ST1_14, ST390_1, ST10_1, ST1_37, ST1_29, ST7_2, ST7_3, ST1_56, ST1_44, ST5_2, ST5_3, ST5_1, ST1_8, ST1_16, ST1_57, ST1_43, ST1_38, ST1_35, Paris, ST1_32, ST1_59, ST1_20, ST1_6, ST1_9, ST1_39, ST1_11, ST1_33, ST1_42, ST1_40, ST1_41, ST1_34, ST1_48, ST1_7, ST7_1, ST1_36, ST1_46, ST1_27, ST1_47, ST1_10, ST1_55, ST1_26, ST1_12, ST1_28, ST1_13, ST72_1, ST1_18, ST1_17, ST1_50, ST1_54, ST1_49, ST1_30, ST1_31, ST1_3, ST1_15, ST1_22, ST1_19, ST1_21, ST1_23, ST1_25, ST1_5, ST1_24, ST1_4, ST152_1, ST1_58, ST8_1 | 80 |
| ST1 | 2619241 | 2634290 | ST1_45, ST1_53, ST1_14, ST390_1, ST10_1, ST1_37, ST1_29, ST7_2, ST7_3, ST1_56, ST1_44, ST5_2, ST5_3, ST5_1, ST1_8, ST1_16, ST1_57, ST1_43, ST1_38, ST1_35, Paris, ST1_32, ST1_59, ST1_20, ST1_6, ST1_9, ST1_39, ST1_11, ST1_33, ST1_42, ST1_40, ST1_41, ST1_34, ST1_48, ST1_7, ST7_1, ST1_36, ST1_46, ST1_27, ST1_47, ST1_10, ST1_55, ST1_26, ST1_12, ST1_28, ST1_13, ST72_1, ST1_18, ST1_17, ST1_50, ST1_54, ST1_49, ST1_30, ST1_31, ST1_3, ST1_15, ST1_22, ST1_19, ST1_21, ST1_23, ST1_25, ST1_5, ST1_24, ST1_4, ST152_1, ST1_58, ST8_1 | 49 |
| ST1 | 787507 | 792757 | ST1_45, ST1_53, ST1_14, ST390_1, ST10_1, ST1_37, ST1_29, ST7_2, ST7_3, ST1_56, ST1_44, ST5_2, ST5_3, ST5_1, ST1_8, ST1_16, ST1_57, ST1_43, ST1_38, ST1_35, Paris, ST1_32, ST1_59, ST1_20, ST1_6, ST1_9, ST1_39, ST1_11, ST1_33, ST1_42, ST1_40, ST1_41, ST1_34, ST1_48, ST1_7, ST7_1, ST1_36, ST1_46, ST1_27, ST1_47, ST1_10, ST1_55, ST1_26, ST1_12, ST1_28, ST1_13, ST72_1, ST1_18, ST1_17, ST1_50, ST1_54, ST1_49, ST1_30, ST1_31, ST1_3, ST1_15, ST1_22, ST1_19, ST1_21, ST1_23, ST1_25, ST1_5, ST1_24, ST1_4, ST152_1, ST1_58, ST8_1 | 25 |
| ST1 | 1790015 | 1796290 | ST1_45, ST1_53, ST1_14, ST390_1, ST10_1, ST1_37, ST1_29, ST7_2, ST7_3, ST1_56, ST1_44, ST5_2, ST5_3, ST5_1, ST1_8, ST1_16, ST1_57, ST1_43, ST1_38, ST1_35, Paris, ST1_32, ST1_59, ST1_20, ST1_6, ST1_9, ST1_39, ST1_11, ST1_33, ST1_42, ST1_40, ST1_41, ST1_34, ST1_48, ST1_7, ST7_1, ST1_36, ST1_46, ST1_27, ST1_47, ST1_10, ST1_55, ST1_26, ST1_12, ST1_28, ST1_13, ST72_1, ST1_18, ST1_17, ST1_50, ST1_54, ST1_49, ST1_30, ST1_31, ST1_3, ST1_15, ST1_22, ST1_19, ST1_21, ST1_23, ST1_25, ST1_5, ST1_24, ST1_4, ST152_1, ST1_58, ST8_1 | 17 |
| ST1 | 1530132 | 1552679 | ST1_45, ST1_53, ST1_14, ST390_1, ST10_1, ST1_37, ST1_29, ST7_2, ST7_3, ST1_56, ST1_44, ST5_2, ST5_3, ST5_1, ST1_8, ST1_16, ST1_57, ST1_43, ST1_38, ST1_35, Paris, ST1_32, ST1_59, ST1_20, ST1_6, ST1_9, ST1_39, ST1_11, ST1_33, ST1_42, ST1_40, ST1_41, ST1_34, ST1_48, ST1_7, ST7_1, ST1_36, ST1_46, ST1_27, ST1_47, ST1_10, ST1_55, ST1_26, ST1_12, ST1_28, ST1_13, ST72_1, ST1_18, ST1_17, ST1_50, ST1_54, ST1_49, ST1_30, ST1_31, ST1_3, ST1_15, ST1_22, ST1_19, ST1_21, ST1_23, ST1_25, ST1_5, ST1_24, ST1_4, ST152_1, ST1_58, ST8_1 | 23 |
| ST1 | 2647068 | 2648366 | ST1_45, ST1_53, ST1_14, ST390_1, ST10_1, ST1_37, ST1_29, ST7_2, ST7_3, ST1_56, ST1_44, ST5_2, ST5_3, ST5_1, ST1_8, ST1_16, ST1_57, ST1_43, ST1_38, ST1_35, Paris, ST1_32, ST1_59, ST1_20, ST1_6, ST1_9, ST1_39, ST1_11, ST1_33, ST1_42, ST1_40, ST1_41, ST1_34, ST1_48, ST1_7, ST7_1, ST1_36, ST1_46, ST1_27, ST1_47, ST1_10, ST1_55, ST1_26, ST1_12, ST1_28, ST1_13, ST72_1, ST1_18, ST1_17, ST1_50, ST1_54, ST1_49, ST1_30, ST1_31, ST1_3, ST1_15, ST1_22, ST1_19, ST1_21, ST1_23, ST1_25, ST1_5, ST1_24, ST1_4, ST152_1, ST1_58, ST8_1 | 12 |
| ST1 | 1573150 | 1584046 | ST1_45, ST1_53, ST1_14, ST390_1, ST10_1, ST1_37, ST1_29, ST7_2, ST7_3, ST1_56, ST1_44, ST5_2, ST5_3, ST5_1, ST1_8, ST1_16, ST1_57, ST1_43, ST1_38, ST1_35, Paris, ST1_32, ST1_59, ST1_20, ST1_6, ST1_9, ST1_39, ST1_11, ST1_33, ST1_42, ST1_40, ST1_41, ST1_34, ST1_48, ST1_7, ST7_1, ST1_36, ST1_46, ST1_27, ST1_47, ST1_10, ST1_55, ST1_26, ST1_12, ST1_28, ST1_13, ST72_1, ST1_18, ST1_17, ST1_50, ST1_54, ST1_49, ST1_30, ST1_31, ST1_3, ST1_15, ST1_22, ST1_19, ST1_21, ST1_23, ST1_25, ST1_5, ST1_24, ST1_4, ST152_1, ST1_58, ST8_1 | 16 |
| ST1 | 1725961 | 1732862 | ST1_45, ST1_53, ST1_14, ST390_1, ST10_1, ST1_37, ST1_29, ST7_2, ST7_3, ST1_56, ST1_44, ST5_2, ST5_3, ST5_1, ST1_8, ST1_16, ST1_57, ST1_43, ST1_38, ST1_35, Paris, ST1_32, ST1_59, ST1_20, ST1_6, ST1_9, ST1_39, ST1_11, ST1_33, ST1_42, ST1_40, ST1_41, ST1_34, ST1_48, ST1_7, ST7_1, ST1_36, ST1_46, ST1_27, ST1_47, ST1_10, ST1_55, ST1_26, ST1_12, ST1_28, ST1_13, ST72_1, ST1_18, ST1_17, ST1_50, ST1_54, ST1_49, ST1_30, ST1_31, ST1_3, ST1_15, ST1_22, ST1_19, ST1_21, ST1_23, ST1_25, ST1_5, ST1_24, ST1_4, ST152_1, ST1_58, ST8_1 | 7 |
| ST1 | 1994336 | 2001091 | ST1_2 | 395 |
| ST1 | 923474 | 933108 | ST1_2 | 49 |
| ST1 | 944824 | 951936 | ST1_2 | 10 |
| ST1 | 888224 | 955432 | ST6_1, ST1_51, ST1_52 | 1465 |
| ST1 | 964978 | 990570 | ST6_1, ST1_51, ST1_52 | 639 |
| ST1 | 471846 | 488208 | ST6_1, ST1_51, ST1_52 | 387 |
| ST1 | 500118 | 510563 | ST6_1, ST1_51, ST1_52 | 252 |
| ST1 | 1994540 | 2003488 | ST6_1, ST1_51, ST1_52 | 53 |
| ST23 | 976393 | 985531 | ST23_33 | 5 |
| ST23 | 1797329 | 1856578 | ST23_32 | 80 |
| ST23 | 985441 | 985732 | ST23_32 | 15 |
| ST23 | 985504 | 987432 | ST23_36 | 40 |
| ST23 | 90282 | 114673 | ST23_27 | 484 |
| ST23 | 985497 | 986686 | ST23_18 | 131 |
| ST23 | 1846432 | 1846814 | ST23_13 | 19 |
| ST23 | 1830646 | 1831372 | ST23_13 | 14 |
| ST23 | 1830565 | 1831357 | ST23_29 | 45 |
| ST23 | 1797278 | 1884363 | ST23_28, ST23_24 | 2847 |
| ST23 | 1917992 | 2029631 | ST23_28, ST23_24 | 3612 |
| ST23 | 348780 | 372623 | ST23_28, ST23_24 | 553 |
| ST23 | 2051646 | 2069269 | ST23_28, ST23_24 | 315 |
| ST23 | 2117394 | 2117673 | ST23_28, ST23_24 | 18 |
| ST23 | 1814773 | 1829614 | ST23_21, ST23_28, ST23_24 | 6 |
| ST23 | 66911 | 82940 | ST23_37 | 433 |
| ST23 | 1403701 | 1414727 | ST23_37 | 393 |
| ST23 | 3167409 | 3169197 | ST23_37 | 47 |
| ST23 | 3141655 | 3142504 | ST23_37 | 14 |
| ST23 | 1813655 | 1832409 | ST23_23 | 2258 |
| ST23 | 66911 | 82940 | ST23_31 | 435 |
| ST23 | 1403701 | 1414727 | ST23_31 | 394 |
| ST23 | 3167409 | 3169197 | ST23_31 | 47 |
| ST23 | 3141655 | 3142504 | ST23_31 | 15 |
| ST23 | 1807703 | 1855253 | ST23_25 | 12 |
| ST23 | 1830646 | 1831372 | ST23_31, ST23_34, ST23_12, ST23_13, ST23_29, ST23_21, ST23_28, ST23_24, ST23_37, ST23_23, ST23_19, ST23_26 | 14 |
| ST23 | 985480 | 987432 | ST23_31, ST23_34, ST23_12, ST23_13, ST23_29, ST23_21, ST23_28, ST23_24, ST23_37, ST23_23, ST23_19, ST23_26 | 5 |
| ST23 | 958018 | 1011415 | ST23_20 | 16 |
| ST23 | 956035 | 989089 | ST23_5 | 954 |
| ST23 | 1846493 | 1846575 | ST23_5 | 14 |
| ST23 | 956035 | 989089 | ST23_3, ST23_4, ST23_2 | 955 |
| ST23 | 956176 | 1011395 | ST23_20, ST23_14 | 190 |
| ST23 | 2576957 | 2668161 | ST23_8 | 2201 |
| ST23 | 2329327 | 2370631 | ST23_8 | 834 |
| ST23 | 2181852 | 2207521 | ST23_8 | 727 |
| ST23 | 956001 | 989219 | ST23_8 | 405 |
| ST23 | 1006723 | 1011356 | ST23_8 | 6 |
| ST23 | 955993 | 1013531 | ST23_7, ST23_6 | 1567 |
| ST23 | 2633019 | 2647930 | ST23_7, ST23_6 | 407 |
| ST23 | 1013926 | 1102772 | ST23_15 | 1972 |
| ST23 | 956013 | 988834 | ST23_15 | 1044 |
| ST23 | 3030760 | 3053831 | ST23_15 | 784 |
| ST23 | 2524129 | 2558938 | ST23_15 | 446 |
| ST23 | 817623 | 820975 | ST23_15 | 85 |
| ST23 | 556940 | 558735 | ST23_15 | 36 |
| ST23 | 988999 | 1003708 | ST23_15 | 30 |
| ST23 | 956029 | 1008701 | ST23_7, ST23_6, ST23_8, ST23_20, ST23_14, ST23_5, ST23_3, ST23_4, ST23_2 | 404 |
| ST23 | 955999 | 1013531 | ST23_35 | 1949 |
| ST23 | 956224 | 985690 | ST23_15, ST23_7, ST23_6, ST23_8, ST23_20, ST23_14, ST23_5, ST23_3, ST23_4, ST23_2 | 256 |
| ST23 | 3409420 | 3414426 | ST23_15, ST23_7, ST23_6, ST23_8, ST23_20, ST23_14, ST23_5, ST23_3, ST23_4, ST23_2 | 55 |
| ST23 | 1814103 | 1831462 | ST23_22 | 133 |
| ST23 | 1606505 | 1644290 | ST23_22, ST23_35, ST23_15, ST23_7, ST23_6, ST23_8, ST23_20, ST23_14, ST23_5, ST23_3, ST23_4, ST23_2 | 807 |
| ST23 | 956011 | 988999 | ST23_22, ST23_35, ST23_15, ST23_7, ST23_6, ST23_8, ST23_20, ST23_14, ST23_5, ST23_3, ST23_4, ST23_2 | 696 |
| ST23 | 997940 | 1000389 | ST23_22, ST23_35, ST23_15, ST23_7, ST23_6, ST23_8, ST23_20, ST23_14, ST23_5, ST23_3, ST23_4, ST23_2 | 49 |
| ST23 | 836651 | 839908 | ST23_22, ST23_35, ST23_15, ST23_7, ST23_6, ST23_8, ST23_20, ST23_14, ST23_5, ST23_3, ST23_4, ST23_2 | 50 |
| ST23 | 3163617 | 3165765 | ST23_22, ST23_35, ST23_15, ST23_7, ST23_6, ST23_8, ST23_20, ST23_14, ST23_5, ST23_3, ST23_4, ST23_2 | 33 |
| ST23 | 3141267 | 3155373 | ST23_22, ST23_35, ST23_15, ST23_7, ST23_6, ST23_8, ST23_20, ST23_14, ST23_5, ST23_3, ST23_4, ST23_2 | 45 |
| ST23 | 2950491 | 2951452 | ST23_22, ST23_35, ST23_15, ST23_7, ST23_6, ST23_8, ST23_20, ST23_14, ST23_5, ST23_3, ST23_4, ST23_2 | 17 |
| ST23 | 1846190 | 1846818 | ST23_22, ST23_35, ST23_15, ST23_7, ST23_6, ST23_8, ST23_20, ST23_14, ST23_5, ST23_3, ST23_4, ST23_2 | 12 |
| ST23 | 840498 | 850140 | ST23_22, ST23_35, ST23_15, ST23_7, ST23_6, ST23_8, ST23_20, ST23_14, ST23_5, ST23_3, ST23_4, ST23_2 | 8 |
| ST23 | 3390687 | 3396535 | ST23_11 | 187 |
| ST23 | 1606505 | 1646261 | ST23_30 | 833 |
| ST23 | 3027996 | 3056737 | ST23_30 | 680 |
| ST23 | 3190999 | 3202634 | ST23_30 | 395 |
| ST23 | 266794 | 288600 | ST23_30 | 391 |
| ST23 | 2426847 | 2457998 | ST23_30 | 295 |
| ST23 | 959845 | 969080 | ST23_30 | 121 |
| ST23 | 717479 | 726819 | ST23_30 | 99 |
| ST23 | 2044854 | 2054140 | ST23_30 | 70 |
| ST23 | 2829904 | 2853292 | ST23_30 | 88 |
| ST23 | 836651 | 839908 | ST23_30 | 50 |
| ST23 | 3216995 | 3217242 | ST23_30 | 25 |
| ST23 | 604873 | 630184 | ST23_30 | 57 |
| ST23 | 590958 | 601310 | ST23_30 | 32 |
| ST23 | 2922362 | 2922713 | ST23_30 | 16 |
| ST23 | 2271727 | 2279037 | ST23_30 | 26 |
| ST23 | 1846126 | 1846507 | ST23_30 | 12 |
| ST23 | 2415602 | 2416202 | ST23_30 | 15 |
| ST23 | 1025489 | 1027412 | ST23_30 | 17 |
| ST23 | 2489553 | 2502274 | ST23_30 | 22 |
| ST23 | 3172401 | 3190821 | ST23_30 | 23 |
| ST23 | 1472852 | 1480646 | ST23_30 | 14 |
| ST23 | 1428645 | 1429483 | ST23_30 | 6 |
| ST23 | 1440479 | 1440986 | ST23_30 | 5 |
| ST23 | 840498 | 850140 | ST23_30 | 8 |
| ST23 | 1797278 | 1831171 | ST23_27, ST23_10, ST23_33, ST23_1, ST23_32, ST23_36, ST23_16, ST23_17, ST23_9, ST23_18 | 177 |
| ST37 | 2178935 | 2217298 | ST37_49 | 776 |
| ST37 | 2227496 | 2240252 | ST37_49 | 308 |
| ST37 | 2251145 | 2252875 | ST37_49 | 58 |
| ST37 | 1171522 | 1171565 | ST37_41 | 11 |
| ST37 | 3201398 | 3243429 | Philadelphia | 650 |
| ST37 | 1932065 | 1980125 | Philadelphia | 527 |
| ST37 | 144005 | 145613 | Philadelphia | 82 |
| ST37 | 3254841 | 3267287 | Philadelphia | 77 |
| ST37 | 2104525 | 2106820 | Philadelphia | 51 |
| ST37 | 66021 | 66446 | Philadelphia | 35 |
| ST37 | 1319674 | 1320016 | Philadelphia | 9 |
| ST37 | 1931856 | 1980370 | ST37_27, ST37_5, ST37_4, ST37_59, ST37_63, ST37_61, ST37_3 | 406 |
| ST37 | 3201384 | 3243492 | ST37_27, ST37_5, ST37_4, ST37_59, ST37_63, ST37_61, ST37_3 | 321 |
| ST37 | 3254398 | 3282008 | ST37_27, ST37_5, ST37_4, ST37_59, ST37_63, ST37_61, ST37_3 | 262 |
| ST37 | 1688612 | 1688976 | ST37_27, ST37_5, ST37_4, ST37_59, ST37_63, ST37_61, ST37_3 | 19 |
| ST37 | 1068126 | 1141247 | Philadelphia, ST37_27, ST37_5, ST37_4, ST37_59, ST37_63, ST37_61, ST37_3 | 2587 |
| ST37 | 2079705 | 2134649 | Philadelphia, ST37_27, ST37_5, ST37_4, ST37_59, ST37_63, ST37_61, ST37_3 | 2112 |
| ST37 | 3187272 | 3262236 | Philadelphia, ST37_27, ST37_5, ST37_4, ST37_59, ST37_63, ST37_61, ST37_3 | 1231 |
| ST37 | 1932213 | 2001991 | Philadelphia, ST37_27, ST37_5, ST37_4, ST37_59, ST37_63, ST37_61, ST37_3 | 1030 |
| ST37 | 1311340 | 1343469 | Philadelphia, ST37_27, ST37_5, ST37_4, ST37_59, ST37_63, ST37_61, ST37_3 | 675 |
| ST37 | 3161730 | 3169180 | Philadelphia, ST37_27, ST37_5, ST37_4, ST37_59, ST37_63, ST37_61, ST37_3 | 51 |
| ST37 | 2316811 | 2319591 | Philadelphia, ST37_27, ST37_5, ST37_4, ST37_59, ST37_63, ST37_61, ST37_3 | 32 |
| ST37 | 3262538 | 3263151 | Philadelphia, ST37_27, ST37_5, ST37_4, ST37_59, ST37_63, ST37_61, ST37_3 | 5 |
| ST37 | 1191023 | 1191887 | ST37_45 | 43 |
| ST37 | 1154610 | 1154967 | ST37_20 | 23 |
| ST37 | 1506150 | 1521895 | ST37_15, ST37_23 | 365 |
| ST37 | 132318 | 136323 | ST37_15, ST37_23 | 90 |
| ST37 | 858188 | 859355 | ST37_15, ST37_23 | 27 |
| ST37 | 36858 | 134211 | ST37_72, ST37_42, ST37_71, ST37_58, ST37_57, ST37_17, ST37_7, ST37_22, ST37_44, ST37_43, ST37_56, ST37_13, ST37_12, ST37_62, ST37_69, ST37_70, ST37_8, ST37_6, ST37_46, ST37_60, ST37_54, ST37_39, ST37_67, ST37_68, ST37_66, ST37_53, ST37_36, ST37_11, ST37_25, ST37_18, ST37_41, ST37_19, ST37_48, ST37_47, Philadelphia, ST37_27, ST37_5, ST37_4, ST37_59, ST37_63, ST37_61, ST37_3, ST37_55, ST37_9, ST37_40, ST37_26, ST37_50, ST37_34, ST37_37, ST37_21, ST37_65, ST37_45, ST37_20 | 3280 |
| ST37 | 1032040 | 1057994 | ST37_72, ST37_42, ST37_71, ST37_58, ST37_57, ST37_17, ST37_7, ST37_22, ST37_44, ST37_43, ST37_56, ST37_13, ST37_12, ST37_62, ST37_69, ST37_70, ST37_8, ST37_6, ST37_46, ST37_60, ST37_54, ST37_39, ST37_67, ST37_68, ST37_66, ST37_53, ST37_36, ST37_11, ST37_25, ST37_18, ST37_41, ST37_19, ST37_48, ST37_47, Philadelphia, ST37_27, ST37_5, ST37_4, ST37_59, ST37_63, ST37_61, ST37_3, ST37_55, ST37_9, ST37_40, ST37_26, ST37_50, ST37_34, ST37_37, ST37_21, ST37_65, ST37_45, ST37_20 | 335 |
| ST37 | 752294 | 769367 | ST37_72, ST37_42, ST37_71, ST37_58, ST37_57, ST37_17, ST37_7, ST37_22, ST37_44, ST37_43, ST37_56, ST37_13, ST37_12, ST37_62, ST37_69, ST37_70, ST37_8, ST37_6, ST37_46, ST37_60, ST37_54, ST37_39, ST37_67, ST37_68, ST37_66, ST37_53, ST37_36, ST37_11, ST37_25, ST37_18, ST37_41, ST37_19, ST37_48, ST37_47, Philadelphia, ST37_27, ST37_5, ST37_4, ST37_59, ST37_63, ST37_61, ST37_3, ST37_55, ST37_9, ST37_40, ST37_26, ST37_50, ST37_34, ST37_37, ST37_21, ST37_65, ST37_45, ST37_20 | 289 |
| ST62 | 3433588 | 3438560 | ST62_3 | 219 |
| ST62 | 1149624 | 1166073 | ST62_3 | 203 |
| ST62 | 228615 | 242892 | ST62_3 | 116 |
| ST62 | 1429096 | 1431644 | ST62_3 | 12 |
| ST62 | 368570 | 462791 | ST62_27 | 3280 |
| ST62 | 228098 | 314450 | ST62_27 | 1348 |
| ST62 | 3170546 | 3206895 | ST62_27 | 1003 |
| ST62 | 1156655 | 1165992 | ST62_27 | 183 |
| ST62 | 867760 | 873436 | ST62_27 | 119 |
| ST62 | 1630356 | 1637484 | ST62_27 | 111 |
| ST62 | 1063765 | 1067598 | ST62_27 | 84 |
| ST62 | 890398 | 894072 | ST62_27 | 63 |
| ST62 | 3392344 | 3394497 | ST62_27 | 55 |
| ST62 | 530841 | 532289 | ST62_27 | 28 |
| ST62 | 3438457 | 3446175 | ST62_27 | 12 |
| ST62 | 1156652 | 1166021 | ST62_8, ST62_6 | 317 |
| ST62 | 228615 | 242892 | ST62_8, ST62_6 | 116 |
| ST62 | 3438329 | 3438370 | ST62_8, ST62_6 | 7 |
| ST62 | 1149615 | 1195510 | ST62_8, ST62_6, ST62_3, ST62_27 | 1104 |
| ST62 | 3433552 | 3438469 | ST62_8, ST62_6, ST62_3, ST62_27 | 414 |
| ST62 | 147352 | 160688 | ST62_8, ST62_6, ST62_3, ST62_27 | 356 |
| ST62 | 1379628 | 1435872 | ST62_8, ST62_6, ST62_3, ST62_27 | 480 |
| ST62 | 2759281 | 2774287 | ST62_8, ST62_6, ST62_3, ST62_27 | 202 |
| ST62 | 228320 | 242988 | ST62_8, ST62_6, ST62_3, ST62_27 | 142 |
| ST62 | 3399337 | 3428427 | ST62_8, ST62_6, ST62_3, ST62_27 | 98 |
| ST62 | 2723954 | 2748089 | ST62_8, ST62_6, ST62_3, ST62_27 | 55 |
| ST62 | 2687464 | 2718429 | ST62_8, ST62_6, ST62_3, ST62_27 | 54 |
| ST62 | 3272862 | 3274765 | ST62_8, ST62_6, ST62_3, ST62_27 | 8 |
| ST62 | 3017380 | 3020613 | ST62_8, ST62_6, ST62_3, ST62_27 | 7 |
| ST62 | 1152771 | 1195531 | ST62_2 | 2987 |
| ST62 | 2714479 | 2749216 | ST62_2 | 888 |
| ST62 | 631434 | 645197 | ST62_2 | 335 |
| ST62 | 1263011 | 1333073 | ST62_2 | 600 |
| ST62 | 780636 | 791437 | ST62_2 | 257 |
| ST62 | 902972 | 908216 | ST62_2 | 98 |
| ST62 | 799134 | 806247 | ST62_2 | 24 |
| ST62 | 1149710 | 1149786 | ST62_2 | 6 |
| ST62 | 375499 | 465012 | ST62_19, ST62_1 | 2153 |
| ST62 | 1378450 | 1416747 | ST62_19, ST62_1 | 1697 |
| ST62 | 2720527 | 2774817 | ST62_19, ST62_1 | 1663 |
| ST62 | 1437904 | 1476733 | ST62_19, ST62_1 | 1214 |
| ST62 | 2158924 | 2172137 | ST62_19, ST62_1 | 365 |
| ST62 | 1153093 | 1195506 | ST62_19, ST62_1 | 191 |
| ST62 | 1699789 | 1705030 | ST62_19, ST62_1 | 122 |
| ST62 | 1425699 | 1464861 | ST62_12 | 1457 |
| ST62 | 1153024 | 1175743 | ST62_12 | 728 |
| ST62 | 1425699 | 1476450 | ST62_18 | 1421 |
| ST62 | 1153478 | 1175602 | ST62_18 | 993 |
| ST62 | 2548298 | 2570818 | ST62_18 | 268 |
| ST62 | 3343227 | 3353492 | ST62_18 | 74 |
| ST62 | 3433552 | 3438469 | ST62_35, ST62_34, ST62_33 | 249 |
| ST62 | 1437904 | 1476733 | ST62_21, ST62_22, ST62_30, ST62_26 | 1476 |
| ST62 | 1152889 | 1195510 | ST62_21, ST62_22, ST62_30, ST62_26 | 943 |
| ST62 | 1175881 | 1195533 | ST62_23 | 658 |
| ST62 | 521424 | 533744 | ST62_23 | 446 |
| ST62 | 1176426 | 1192215 | ST62_21, ST62_22, ST62_30, ST62_26, ST62_23 | 13 |
| ST62 | 1153462 | 1175596 | ST62_18, ST62_19, ST62_1, ST62_12 | 582 |
| ST62 | 1464388 | 1465565 | ST62_18, ST62_19, ST62_1, ST62_12 | 51 |
| ST62 | 2039693 | 2041590 | ST62_18, ST62_19, ST62_1, ST62_12 | 29 |
| ST62 | 1445520 | 1446049 | ST62_18, ST62_19, ST62_1, ST62_12 | 12 |
| ST62 | 1149708 | 1177025 | ST62_8, ST62_6, ST62_3, ST62_27, ST62_2 | 312 |
| ST62 | 1189782 | 1195503 | ST62_8, ST62_6, ST62_3, ST62_27, ST62_2 | 202 |
| ST62 | 2725537 | 2749257 | ST62_8, ST62_6, ST62_3, ST62_27, ST62_2 | 40 |
| ST62 | 3441069 | 3441111 | ST62_7 | 12 |
| ST62 | 1149615 | 1176985 | ST62_14, ST62_5 | 226 |
| ST62 | 3435296 | 3436262 | ST62_14, ST62_5 | 16 |
| ST62 | 1149641 | 1195508 | ST62_24 | 1694 |
| ST62 | 3435332 | 3441450 | ST62_24 | 67 |
| ST62 | 1201694 | 1201728 | ST62_24 | 7 |
| ST62 | 1386992 | 1387002 | ST62_24 | 5 |
| ST62 | 1176936 | 1176985 | ST62_13 | 10 |
| ST62 | 1154995 | 1193634 | ST62_7, ST62_14, ST62_5, ST62_9, ST62_15 | 251 |
| ST62 | 3435297 | 3436848 | ST62_7, ST62_14, ST62_5, ST62_9, ST62_15 | 33 |
| ST62 | 1152846 | 1195507 | ST62_29 | 1083 |
| ST62 | 3433552 | 3441684 | ST62_7, ST62_14, ST62_5, ST62_9, ST62_15, ST62_24, ST62_13 | 480 |
| ST62 | 1149641 | 1195285 | ST62_7, ST62_14, ST62_5, ST62_9, ST62_15, ST62_24, ST62_13 | 481 |
| ST62 | 1155018 | 1175304 | ST62_20, ST62_29, ST62_7, ST62_14, ST62_5, ST62_9, ST62_15, ST62_24, ST62_13, ST62_16 | 245 |
| ST62 | 1154986 | 1176966 | ST62_28 | 1529 |
| ST62 | 1444445 | 1465496 | ST62_8, ST62_6, ST62_3, ST62_27, ST62_2, ST62_18, ST62_19, ST62_1, ST62_12, ST62_4, ST62_35, ST62_34, ST62_33, ST62_21, ST62_22, ST62_30, ST62_26, ST62_23 | 295 |
| ST62 | 2749257 | 2764154 | ST62_8, ST62_6, ST62_3, ST62_27, ST62_2, ST62_18, ST62_19, ST62_1, ST62_12, ST62_4, ST62_35, ST62_34, ST62_33, ST62_21, ST62_22, ST62_30, ST62_26, ST62_23 | 106 |
| ST62 | 1153105 | 1195510 | ST62_20, ST62_29, ST62_7, ST62_14, ST62_5, ST62_9, ST62_15, ST62_24, ST62_13, ST62_16, ST62_28 | 648 |
| ST62 | 1440024 | 1483947 | ST62_20, ST62_29, ST62_7, ST62_14, ST62_5, ST62_9, ST62_15, ST62_24, ST62_13, ST62_16, ST62_28 | 514 |
| ST62 | 3440589 | 3443338 | ST62_20, ST62_29, ST62_7, ST62_14, ST62_5, ST62_9, ST62_15, ST62_24, ST62_13, ST62_16, ST62_28 | 235 |
| ST62 | 1379942 | 1407362 | ST62_20, ST62_29, ST62_7, ST62_14, ST62_5, ST62_9, ST62_15, ST62_24, ST62_13, ST62_16, ST62_28 | 139 |
| ST62 | 2705598 | 2724770 | ST62_20, ST62_29, ST62_7, ST62_14, ST62_5, ST62_9, ST62_15, ST62_24, ST62_13, ST62_16, ST62_28 | 112 |
| ST62 | 2728848 | 2737877 | ST62_20, ST62_29, ST62_7, ST62_14, ST62_5, ST62_9, ST62_15, ST62_24, ST62_13, ST62_16, ST62_28 | 24 |
| ST62 | 94166 | 155229 | ST62_11 | 609 |
| ST62 | 81064 | 91858 | ST62_11 | 14 |
| ST62 | 1140227 | 1140266 | ST62_11 | 4 |
| ST62 | 126884 | 160045 | ST62_25, ST62_17, ST62_31, ST62_32 | 413 |
| ST62 | 1442984 | 1465496 | ST62_8, ST62_6, ST62_3, ST62_27, ST62_2, ST62_18, ST62_19, ST62_1, ST62_12, ST62_4, ST62_35, ST62_34, ST62_33, ST62_21, ST62_22, ST62_30, ST62_26, ST62_23, ST62_20, ST62_29, ST62_7, ST62_14, ST62_5, ST62_9, ST62_15, ST62_24, ST62_13, ST62_16, ST62_28 | 861 |
| ST62 | 2857470 | 2911359 | ST62_8, ST62_6, ST62_3, ST62_27, ST62_2, ST62_18, ST62_19, ST62_1, ST62_12, ST62_4, ST62_35, ST62_34, ST62_33, ST62_21, ST62_22, ST62_30, ST62_26, ST62_23, ST62_20, ST62_29, ST62_7, ST62_14, ST62_5, ST62_9, ST62_15, ST62_24, ST62_13, ST62_16, ST62_28 | 669 |
| ST62 | 94166 | 118793 | ST62_8, ST62_6, ST62_3, ST62_27, ST62_2, ST62_18, ST62_19, ST62_1, ST62_12, ST62_4, ST62_35, ST62_34, ST62_33, ST62_21, ST62_22, ST62_30, ST62_26, ST62_23, ST62_20, ST62_29, ST62_7, ST62_14, ST62_5, ST62_9, ST62_15, ST62_24, ST62_13, ST62_16, ST62_28 | 398 |
| ST62 | 39106 | 69951 | ST62_8, ST62_6, ST62_3, ST62_27, ST62_2, ST62_18, ST62_19, ST62_1, ST62_12, ST62_4, ST62_35, ST62_34, ST62_33, ST62_21, ST62_22, ST62_30, ST62_26, ST62_23, ST62_20, ST62_29, ST62_7, ST62_14, ST62_5, ST62_9, ST62_15, ST62_24, ST62_13, ST62_16, ST62_28 | 218 |
| ST62 | 127054 | 155229 | ST62_8, ST62_6, ST62_3, ST62_27, ST62_2, ST62_18, ST62_19, ST62_1, ST62_12, ST62_4, ST62_35, ST62_34, ST62_33, ST62_21, ST62_22, ST62_30, ST62_26, ST62_23, ST62_20, ST62_29, ST62_7, ST62_14, ST62_5, ST62_9, ST62_15, ST62_24, ST62_13, ST62_16, ST62_28 | 181 |
| ST62 | 2185322 | 2224403 | ST62_8, ST62_6, ST62_3, ST62_27, ST62_2, ST62_18, ST62_19, ST62_1, ST62_12, ST62_4, ST62_35, ST62_34, ST62_33, ST62_21, ST62_22, ST62_30, ST62_26, ST62_23, ST62_20, ST62_29, ST62_7, ST62_14, ST62_5, ST62_9, ST62_15, ST62_24, ST62_13, ST62_16, ST62_28 | 172 |
| ST62 | 2039693 | 2065786 | ST62_8, ST62_6, ST62_3, ST62_27, ST62_2, ST62_18, ST62_19, ST62_1, ST62_12, ST62_4, ST62_35, ST62_34, ST62_33, ST62_21, ST62_22, ST62_30, ST62_26, ST62_23, ST62_20, ST62_29, ST62_7, ST62_14, ST62_5, ST62_9, ST62_15, ST62_24, ST62_13, ST62_16, ST62_28 | 70 |
| ST62 | 1996411 | 2033587 | ST62_8, ST62_6, ST62_3, ST62_27, ST62_2, ST62_18, ST62_19, ST62_1, ST62_12, ST62_4, ST62_35, ST62_34, ST62_33, ST62_21, ST62_22, ST62_30, ST62_26, ST62_23, ST62_20, ST62_29, ST62_7, ST62_14, ST62_5, ST62_9, ST62_15, ST62_24, ST62_13, ST62_16, ST62_28 | 68 |
| ST62 | 169519 | 170011 | ST62_8, ST62_6, ST62_3, ST62_27, ST62_2, ST62_18, ST62_19, ST62_1, ST62_12, ST62_4, ST62_35, ST62_34, ST62_33, ST62_21, ST62_22, ST62_30, ST62_26, ST62_23, ST62_20, ST62_29, ST62_7, ST62_14, ST62_5, ST62_9, ST62_15, ST62_24, ST62_13, ST62_16, ST62_28 | 19 |
| ST62 | 1187934 | 1187985 | ST62_8, ST62_6, ST62_3, ST62_27, ST62_2, ST62_18, ST62_19, ST62_1, ST62_12, ST62_4, ST62_35, ST62_34, ST62_33, ST62_21, ST62_22, ST62_30, ST62_26, ST62_23, ST62_20, ST62_29, ST62_7, ST62_14, ST62_5, ST62_9, ST62_15, ST62_24, ST62_13, ST62_16, ST62_28 | 13 |
| ST62 | 16094 | 37372 | ST62_8, ST62_6, ST62_3, ST62_27, ST62_2, ST62_18, ST62_19, ST62_1, ST62_12, ST62_4, ST62_35, ST62_34, ST62_33, ST62_21, ST62_22, ST62_30, ST62_26, ST62_23, ST62_20, ST62_29, ST62_7, ST62_14, ST62_5, ST62_9, ST62_15, ST62_24, ST62_13, ST62_16, ST62_28 | 32 |
| ST62 | 2738280 | 2744046 | ST62_8, ST62_6, ST62_3, ST62_27, ST62_2, ST62_18, ST62_19, ST62_1, ST62_12, ST62_4, ST62_35, ST62_34, ST62_33, ST62_21, ST62_22, ST62_30, ST62_26, ST62_23, ST62_20, ST62_29, ST62_7, ST62_14, ST62_5, ST62_9, ST62_15, ST62_24, ST62_13, ST62_16, ST62_28 | 18 |
| ST62 | 2167575 | 2175085 | ST62_8, ST62_6, ST62_3, ST62_27, ST62_2, ST62_18, ST62_19, ST62_1, ST62_12, ST62_4, ST62_35, ST62_34, ST62_33, ST62_21, ST62_22, ST62_30, ST62_26, ST62_23, ST62_20, ST62_29, ST62_7, ST62_14, ST62_5, ST62_9, ST62_15, ST62_24, ST62_13, ST62_16, ST62_28 | 19 |
| ST62 | 74878 | 91054 | ST62_8, ST62_6, ST62_3, ST62_27, ST62_2, ST62_18, ST62_19, ST62_1, ST62_12, ST62_4, ST62_35, ST62_34, ST62_33, ST62_21, ST62_22, ST62_30, ST62_26, ST62_23, ST62_20, ST62_29, ST62_7, ST62_14, ST62_5, ST62_9, ST62_15, ST62_24, ST62_13, ST62_16, ST62_28 | 21 |
| ST62 | 2773595 | 2773935 | ST62_8, ST62_6, ST62_3, ST62_27, ST62_2, ST62_18, ST62_19, ST62_1, ST62_12, ST62_4, ST62_35, ST62_34, ST62_33, ST62_21, ST62_22, ST62_30, ST62_26, ST62_23, ST62_20, ST62_29, ST62_7, ST62_14, ST62_5, ST62_9, ST62_15, ST62_24, ST62_13, ST62_16, ST62_28 | 8 |
| ST62 | 418830 | 455329 | ST62_10 | 1656 |
| ST62 | 3433551 | 3445309 | ST62_10 | 529 |
| ST62 | 120657 | 160626 | ST62_10 | 529 |
| ST62 | 3077327 | 3082704 | ST62_10 | 219 |
| ST62 | 3135777 | 3139250 | ST62_10 | 59 |
| ST62 | 1149510 | 1149625 | ST62_10 | 25 |
| ST62 | 1539808 | 1539824 | ST62_10 | 5 |
