# Supplementary material for: Multiple major disease-associated clones of Legionella pneumophila have emerged recently and independently
Source: Genome Res. 2016 Nov;26(11):1555–64. doi: 10.1101/gr.209536.116 (PMC5088597; doi:10.1101/gr.209536.116)
Supplement: Supplemental Material [file supp_gr.209536.116_Supplemental_Table_S9.docx]

Table S9. Distribution of the known Dot/Icm substrates in the five STs.

| **Gene** | **Name/Function** | **ST1** | **ST23** | **ST37** | **ST47** | **ST62** |
| --- | --- | --- | --- | --- | --- | --- |
| *lpg0008* | hypothetical protein | + | + | + | + | + |
| *lpg0012* | hypothetical protein | + | + | + | + | + |
| *lpg0021* | hypothetical protein | + | + | + | + | + |
| *lpg0030* | hypothetical protein | + | + | + | + | + |
| *lpg0038* | ankyrin repeat-containing protein | + | + | + | + | + |
| *lpg0041* | hypothetical protein | Absent | Absent | + | Absent | Absent |
| *lpg0045* | hypothetical protein | + | + | + | + | + |
| *lpg0046* | hypothetical protein | + | + | + | + | + |
| *lpg0059* | hypothetical protein | + | + | + | + | + |
| *lpg0080* | hypothetical protein | + | + | + | + | Absent |
| *lpg0081* | hypothetical protein | + | + | + | + | Absent |
| *lpg0090* | hypothetical protein | + | + | + | + | + |
| *lpg0096* | hypothetical protein | + | + | + | + | + |
| *lpg0103* | GNAT family acetyltransferase | + | + | + | + | + |
| *lpg0107* | hypothetical protein | + | + | + | + | + |
| *lpg0126* | hypothetical protein | + | + | + | + | + |
| *lpg0130* | hypothetical protein | + | + | + | + | + |
| *lpg0135* | hypothetical protein | + | + | + | + | + |
| *lpg0140* | hypothetical protein | + | + | + | + | + |
| *lpg0160* | hypothetical protein | + | + | + | + | + |
| *lpg0170* | ABC transporter permease | + | + | + | + | + |
| *lpg0171* | hypothetical protein | + | + | + | + | + |
| *lpg0172* | hypothetical protein | + | + | + | + | + |
| *lpg0181* | hypothetical protein | + | + | + | + | + |
| *lpg0191* | hypothetical protein | + | + | + | + | + |
| *lpg0195* | hypothetical protein | + | + | + | + | + |
| *lpg0196* | hypothetical protein | + | + | + | + | + |
| *lpg0208* | serine/threonine-protein kinase | + | + | + | + | + |
| *lpg0209* | hypothetical protein | + | + | + | + | + |
| *lpg0210* | hypothetical protein | + | + | + | + | + |
| *lpg0227* | hypothetical protein | + | + | + | + | + |
| *lpg0234* | protein SidE | + | + | + | + | + |
| *lpg0240* | DNA repair protein | + | + | + | + | Variable |
| *lpg0246* | hypothetical protein | + | + | + | + | + |
| *lpg0254* | hypothetical protein | + | + | + | + | + |
| *lpg0257* | multidrug resistance secretion protein | + | + | + | + | + |
| *lpg0260* | hypothetical protein | + | + | + | + | + |
| *lpg0275* | protein SdbA | + | + | + | + | + |
| *lpg0276* | hypothetical protein | + | + | + | + | + |
| *lpg0284* | hypothetical protein | + | + | + | + | + |
| *lpg0285* | hypothetical protein | + | + | + | + | + |
| *lpg0294* | hypothetical protein | + | + | + | + | + |
| *lpg0364* | hypothetical protein | + | + | + | + | + |
| *lpg0365* | hypothetical protein | + | + | + | + | + |
| *lpg0375* | hypothetical protein | + | + | + | + | + |
| *lpg0376* | protein SdhA | + | + | + | + | + |
| *lpg0390* | VipA | + | + | + | + | + |
| *lpg0393* | hypothetical protein | + | + | + | + | + |
| *lpg0401* | hypothetical protein | + | + | + | + | + |
| *lpg0402* | ankyrin repeat-containing protein | + | + | + | + | + |
| *lpg0403* | ankyrin repeat-containing protein | + | + | + | + | + |
| *lpg0405* | hypothetical protein | + | + | + | + | + |
| *lpg0422* | glucoamylase | + | + | + | + | + |
| *lpg0436* | ankyrin repeat-containing protein | + | + | + | + | + |
| *lpg0437* | hypothetical protein | + | + | + | + | + |
| *lpg0439* | hypothetical protein | + | + | + | + | + |
| *lpg0483* | ankyrin repeat-containing protein | + | + | + | + | + |
| *lpg0515* | phytanoyl-CoA dioxygenase | + | + | + | + | + |
| *lpg0518* | hypothetical protein | + | + | + | + | + |
| *lpg0519* | hypothetical protein | Absent | Absent | + | Absent | Absent |
| *lpg0621* | protein SidA | + | + | + | + | + |
| *lpg0634* | hypothetical protein | + | + | + | + | + |
| *lpg0642* | hypothetical protein | + | + | + | + | + |
| *lpg0645* | hypothetical protein | + | + | + | + | + |
| *lpg0695* | ankyrin repeat-containing protein | + | + | + | + | + |
| *lpg0696* | hypothetical protein | + | + | + | + | + |
| *lpg0716* | hypothetical protein | + | + | + | + | + |
| *lpg0733* | hypothetical protein | + | + | + | + | + |
| *lpg0796* | hypothetical protein | + | Absent | + | Absent | Absent |
| *lpg0898* | hypothetical protein | + | + | + | + | + |
| *lpg0921* | hypothetical protein | + | + | + | + | + |
| *lpg0926* | hypothetical protein | + | + | + | + | + |
| *lpg0940* | LidA | + | + | + | + | + |
| *lpg0944* | hypothetical protein | + | Absent | + | + | Variable |
| *lpg0945* | Gala protein type 1, 3 or 4 | + | + | + | + | + |
| *lpg0963* | hypothetical protein | + | + | + | + | + |
| *lpg0967* | hypothetical protein | + | + | + | + | + |
| *lpg0968* | hypothetical protein | + | + | + | + | + |
| *lpg0969* | hypothetical protein | + | + | + | + | + |
| *lpg1083* | hypothetical protein | Absent | Absent | + | Absent | Absent |
| *lpg1101* | hypothetical protein | + | + | + | + | + |
| *lpg1106* | hypothetical protein | + | + | + | + | + |
| *lpg1108* | lipase | + | + | + | + | + |
| *lpg1109* | hypothetical protein | + | + | + | + | + |
| *lpg1110* | hypothetical protein | + | + | + | + | + |
| *lpg1111* | hypothetical protein | + | + | + | + | + |
| *lpg1120* | hypothetical protein | + | + | + | + | + |
| *lpg1121* | hypothetical protein | + | + | + | + | + |
| *lpg1124* | hypothetical protein | + | + | + | + | + |
| *lpg1129* | hypothetical protein | + | + | + | + | + |
| *lpg1137* | hypothetical protein | + | + | + | + | + |
| *lpg1144* | hypothetical protein | + | + | + | + | + |
| *lpg1145* | hypothetical protein | + | + | + | + | + |
| *lpg1147* | hypothetical protein | + | + | + | + | + |
| *lpg1148* | hypothetical protein | + | + | + | + | + |
| *lpg1152* | hypothetical protein | + | + | + | + | + |
| *lpg1154* | hypothetical protein | + | + | + | + | + |
| *lpg1158* | hypothetical protein | + | + | + | + | + |
| *lpg1166* | hypothetical protein | + | + | + | + | + |
| *lpg1171* | hypothetical protein | + | + | + | + | + |
| *lpg1183* | hypothetical protein | + | + | + | + | + |
| *lpg1227* | hypothetical protein | + | + | + | + | + |
| *lpg1273* | hypothetical protein | + | + | + | + | + |
| *lpg1290* | hypothetical protein | + | Absent | + | Absent | Absent |
| *lpg1312* | coiled-coil-containing protein | Absent | Absent | + | Absent | Absent |
| *lpg1316* | hypothetical protein | Absent | Absent | + | Absent | Absent |
| *lpg1317* | hypothetical protein | Absent | Absent | + | Absent | Absent |
| *lpg1328* | thaumatin domain-containing protein | + | + | + | + | + |
| *lpg1354* | hypothetical protein | + | + | + | Absent | Absent |
| *lpg1355* | protein SidG | + | + | + | Absent | Absent |
| *lpg1356* | hypothetical protein | + | + | + | + | + |
| *lpg1368* | hypothetical protein | + | + | + | + | + |
| *lpg1408* | choline kinase | + | + | + | + | + |
| *lpg1426* | hypothetical protein | + | + | + | + | + |
| *lpg1449* | hypothetical protein | + | + | + | + | + |
| *lpg1453* | hypothetical protein | + | + | + | + | + |
| *lpg1483* | serine/threonine-protein kinase | + | + | + | + | + |
| *lpg1484* | hypothetical protein | + | + | + | + | + |
| *lpg1488* | coiled-coil-containing protein | + | + | + | + | + |
| *lpg1489* | hypothetical protein | + | + | + | + | + |
| *lpg1491* | hypothetical protein | + | + | + | + | + |
| *lpg1496* | hypothetical protein | + | + | + | + | + |
| *lpg1551* | hypothetical protein | + | + | + | + | + |
| *lpg1578* | hypothetical protein | + | + | + | + | + |
| *lpg1588* | hypothetical protein | + | + | + | + | + |
| *lpg1598* | hypothetical protein | + | + | + | + | + |
| *lpg1602* | hypothetical protein | + | + | + | + | + |
| *lpg1621* | hypothetical protein | + | + | + | + | + |
| *lpg1625* | hypothetical protein | + | + | + | + | + |
| *lpg1639* | hypothetical protein | + | + | + | + | + |
| *lpg1642* | protein SidB | + | + | + | + | + |
| *lpg1654* | hypothetical protein | + | + | + | Absent | + |
| *lpg1660* | hypothetical protein | + | + | + | + | + |
| *lpg1661* | hypothetical protein | + | + | + | + | + |
| *lpg1663* | hypothetical protein | + | + | + | + | + |
| *lpg1666* | hypothetical protein | + | + | + | + | + |
| *lpg1667* | hypothetical protein | + | + | + | + | + |
| *lpg1670* | hypothetical protein | + | + | + | + | + |
| *lpg1683* | hypothetical protein | Absent | Absent | + | Absent | Absent |
| *lpg1684* | hypothetical protein | Absent | Absent | + | Absent | Absent |
| *lpg1685* | hypothetical protein | Absent | Absent | + | Absent | Absent |
| *lpg1687* | hypothetical protein | + | + | + | + | + |
| *lpg1689* | hypothetical protein | + | + | + | + | + |
| *lpg1692* | hypothetical protein | + | Absent | Variable | + | Absent |
| *lpg1701* | kinectin 1 | + | + | + | + | + |
| *lpg1702* | hypothetical protein | + | + | + | + | + |
| *lpg1716* | hypothetical protein | + | + | + | + | + |
| *lpg1717* | hypothetical protein | + | + | + | + | + |
| *lpg1718* | hypothetical protein | + | + | + | + | + |
| *lpg1751* | hypothetical protein | + | + | + | + | + |
| *lpg1752* | hypothetical protein | + | + | + | + | + |
| *lpg1776* | hypothetical protein | + | + | + | + | + |
| *lpg1797* | hypothetical protein | + | + | + | + | + |
| *lpg1798* | hypothetical protein | + | + | + | + | + |
| *lpg1803* | hypothetical protein | + | + | + | + | + |
| *lpg1822* | hypothetical protein | + | + | + | + | + |
| *lpg1836* | coiled coil domain-containing protein | + | + | + | + | + |
| *lpg1851* | hypothetical protein | + | + | + | + | + |
| *lpg1884* | microtubule binding protein | + | + | + | + | + |
| *lpg1888* | hypothetical protein | + | + | + | + | + |
| *lpg1890* | hypothetical protein | + | + | + | + | + |
| *lpg1907* | hypothetical protein | + | + | + | + | + |
| *lpg1924* | hypothetical protein | + | + | + | + | + |
| *lpg1933* | hypothetical protein | + | + | + | + | + |
| *lpg1947* | hypothetical protein | + | + | + | + | Absent |
| *lpg1948* | hypothetical protein | + | + | + | + | + |
| *lpg1949* | hypothetical protein | + | + | + | + | + |
| *lpg1950* | guanine nucleotide exchange protein | + | + | + | + | + |
| *lpg1953* | hypothetical protein | + | + | + | + | + |
| *lpg1958* | hypothetical protein | + | + | + | + | + |
| *lpg1959* | hypothetical protein | + | + | + | + | + |
| *lpg1960* | hypothetical protein | + | + | + | + | + |
| *lpg1962* | peptidyl-prolyl cis-trans isomerase | + | + | + | + | + |
| *lpg1963* | hypothetical protein | Absent | Absent | + | Absent | Absent |
| *lpg1964* | hypothetical protein | Absent | Absent | + | Absent | Absent |
| *lpg1965* | hypothetical protein | + | + | + | + | + |
| *lpg1966* | hypothetical protein | + | + | + | + | + |
| *lpg1969* | hypothetical protein | + | + | + | + | + |
| *lpg1972* | hypothetical protein | + | + | + | + | + |
| *lpg1975* | hypothetical protein | + | + | + | + | + |
| *lpg1976* | UVB-resistance protein UVR8 | + | + | + | + | + |
| *lpg1978* | teichoic acid biosynthesis protein | + | + | + | + | + |
| *lpg1986* | hypothetical protein | + | + | + | + | + |
| *lpg2050* | hypothetical protein | + | + | + | + | + |
| *lpg2131* | ankyrin 3 | Absent | + | + | + | + |
| *lpg2137* | calmodulin-dependent protein kinase | + | + | + | + | + |
| *lpg2144* | F-box protein | + | + | + | + | + |
| *lpg2147* | hypothetical protein | + | + | + | + | + |
| *lpg2148* | hypothetical protein | + | + | + | + | + |
| *lpg2149* | hypothetical protein | + | + | + | + | + |
| *lpg2153* | Sid related protein-like | + | + | + | + | + |
| *lpg2154* | Sid related protein-like | + | + | + | + | + |
| *lpg2155* | hypothetical protein | + | + | + | + | + |
| *lpg2156* | Sid related protein-like | + | + | + | + | + |
| *lpg2157* | Sid related protein-like | + | + | + | + | + |
| *lpg2160* | hypothetical protein | + | + | + | + | + |
| *lpg2164* | hypothetical protein | + | + | + | + | + |
| *lpg2166* | hypothetical protein | + | + | + | + | + |
| *lpg2160* | hypothetical protein | + | + | + | + | + |
| *lpg2176* | sphingosine-1-phosphate lyase I | + | + | + | + | + |
| *lpg2199* | hypothetical protein | + | + | + | + | + |
| *lpg2200* | hypothetical protein | + | + | + | + | + |
| *lpg2215* | ankyrin repeat-containing protein | + | + | + | + | + |
| *lpg2216* | purine NTPase | + | + | + | + | + |
| *lpg2222* | hypothetical protein | + | + | + | + | + |
| *lpg2223* | hypothetical protein | + | + | + | + | + |
| *lpg2224* | UVB-resistance protein UVR8 | + | + | + | + | + |
| *lpg2239* | hypothetical protein | + | + | + | + | + |
| *lpg2244* | hypothetical protein | + | + | + | + | + |
| *lpg2248* | hypothetical protein | + | + | + | + | + |
| *lpg2271* | hypothetical protein | + | + | + | + | + |
| *lpg2283* | hypothetical protein | + | + | + | + | + |
| *lpg2298* | inclusion membrane protein A | + | + | + | + | + |
| *lpg2300* | ankyrin repeat-containing protein | + | + | + | + | + |
| *lpg2311* | interaptin | + | + | + | + | + |
| *lpg2322* | ankyrin repeat-containing protein | + | + | + | + | + |
| *lpg2327* | hypothetical protein | + | + | + | + | + |
| *lpg2328* | hypothetical protein | + | + | + | + | + |
| *lpg2344* | hypothetical protein | + | + | + | + | + |
| *lpg2351* | hypothetical protein | + | + | + | + | + |
| *lpg2359* | hypothetical protein | + | + | + | + | + |
| *lpg2370* | hypothetical protein | + | + | + | + | + |
| *lpg2372* | hypothetical protein | + | + | + | + | + |
| *lpg2375* | hypothetical protein | + | + | + | + | + |
| *lpg2382* | hypothetical protein | + | + | + | + | + |
| *lpg2391* | protein SdbC | + | + | + | + | + |
| *lpg2392* | hypothetical protein | + | + | + | + | + |
| *lpg2400* | hypothetical protein | + | + | + | + | + |
| *lpg2406* | hypothetical protein | + | + | + | + | + |
| *lpg2407* | hypothetical protein | + | + | + | + | + |
| *lpg2409* | hypothetical protein | + | + | + | + | + |
| *lpg2410* | patatin-like phospholipase | + | + | + | + | + |
| *lpg2411* | hypothetical protein | + | + | + | + | + |
| *lpg2416* | ankyrin repeat-containing protein | + | + | + | + | + |
| *lpg2420* | hypothetical protein | + | + | + | + | + |
| *lpg2422* | hypothetical protein | + | + | + | + | + |
| *lpg2424* | hypothetical protein | + | + | + | + | + |
| *lpg2425* | hypothetical protein | + | + | + | + | + |
| *lpg2433* | hypothetical protein | + | + | + | + | + |
| *lpg2434* | hypothetical protein | + | + | + | + | + |
| *lpg2443* | hypothetical protein | + | + | + | + | + |
| *lpg2444* | hypothetical protein | + | + | + | + | + |
| *lpg2452* | ankyrin repeat-containing protein | + | + | + | + | + |
| *lpg2456* | ankyrin repeat-containing protein | + | + | + | + | + |
| *lpg2461* | hypothetical protein | + | + | + | + | + |
| *lpg2464* | hypothetical protein | + | + | + | + | + |
| *lpg2465* | protein SidD | Absent | Absent | + | Absent | Absent |
| *lpg2482* | protein SdbC | + | + | + | + | + |
| *lpg2490* | hypothetical protein | + | + | + | + | + |
| *lpg2482* | protein SdbC | + | + | + | + | + |
| *lpg2498* | hypothetical protein | + | + | + | + | + |
| *lpg2504* | hypothetical protein | + | + | + | + | + |
| *lpg2505* | hypothetical protein | + | + | + | + | + |
| *lpg2508* | hypothetical protein | + | + | + | + | + |
| *lpg2509* | protein SdeD | + | + | + | + | + |
| *lpg2510* | protein SdcA | + | + | + | + | + |
| *lpg2511* | protein SidC, interaptin | + | + | + | + | + |
| *lpg2523* | hypothetical protein | + | + | + | + | + |
| *lpg2525* | hypothetical protein | Absent | Absent | + | Absent | Absent |
| *lpg2526* | hypothetical protein | + | + | + | + | + |
| *lpg2527* | hypothetical protein | + | + | + | + | + |
| *lpg2529* | hypothetical protein | + | + | + | + | + |
| *lpg2538* | hypothetical protein | + | + | + | + | + |
| *lpg2539* | hypothetical protein | + | + | + | + | + |
| *lpg2541* | hypothetical protein | + | + | + | + | + |
| *lpg2546* | hypothetical protein | + | + | + | + | + |
| *lpg2552* | hypothetical protein | + | + | + | + | + |
| *lpg2555* | hypothetical protein | + | + | + | + | + |
| *lpg2556* | protein kinase | + | + | + | + | + |
| *lpg2577* | hypothetical protein | + | + | + | + | + |
| *lpg2584* | inhibitor of growth protein SidF | + | + | + | + | + |
| *lpg2588* | acid sphingomyelinase-like phosphodiesterase | + | + | + | + | + |
| *lpg2591* | hypothetical protein | + | + | + | + | + |
| *lpg2603* | hypothetical protein | + | + | + | + | + |
| *lpg2628* | hypothetical protein | + | + | + | + | + |
| *lpg2637* | hypothetical protein | + | + | + | + | + |
| *lpg2638* | hypothetical protein | + | + | + | + | + |
| *lpg2692* | hypothetical protein | + | + | + | + | + |
| *lpg2694* | phytanoyl-CoA dioxygenase | + | + | + | + | + |
| *lpg2718* | hypothetical protein | + | + | + | + | + |
| *lpg2760* | DNA-binding response regulator | + | + | + | + | + |
| *lpg2744* | hypothetical protein | + | + | + | + | + |
| *lpg2745* | hypothetical protein | + | + | + | + | + |
| *lpg2793* | interaptin | + | + | + | + | + |
| *lpg2804* | hypothetical protein | + | + | + | + | + |
| *lpg2806* | hypothetical protein | + | + | + | + | + |
| *lpg2815* | hypothetical protein | + | + | + | + | + |
| *lpg2826* | hypothetical protein | Absent | + | + | + | + |
| *lpg2828* | hypothetical protein | + | + | + | + | + |
| *lpg2829* | protein SidH | + | + | + | + | + |
| *lpg2830* | UBOX-containing protein | + | + | + | + | + |
| *lpg2831* | VipD | + | + | + | + | + |
| *lpg2832* | hypothetical protein | + | + | + | + | + |
| *lpg2844* | hypothetical protein | Variable | + | + | Variable | + |
| *lpg2862* | coiled-coil containing protein | + | + | + | + | + |
| *lpg2874* | hypothetical protein | + | + | + | + | + |
| *lpg2879* | hypothetical protein | + | + | + | + | + |
| *lpg2884* | hypothetical protein | + | + | + | + | + |
| *lpg2885* | hypothetical protein | + | + | + | + | + |
| *lpg2888* | hypothetical protein | + | + | + | + | + |
| *lpg2907* | hypothetical protein | + | + | + | + | + |
| *lpg2912* | hypothetical protein | + | + | + | + | + |
| *lpg2936* | 16S ribosomal RNA methyltransferase RsmE | + | + | + | + | + |
| *lpg2975* | hypothetical protein | + | + | + | + | + |
| *lpg2999* | hypothetical protein | + | + | + | + | + |
| *lpg3000* | hypothetical protein | + | + | + | + | + |
