# Supplementary material for: Multiple major disease-associated clones of Legionella pneumophila have emerged recently and independently
Source: Genome Res. 2016 Nov;26(11):1555–64. doi: 10.1101/gr.209536.116 (PMC5088597; doi:10.1101/gr.209536.116)
Supplement: Supplemental Material [file supp_gr.209536.116_Supplemental_Table_S10.docx]

Table S10. Homoplasic SNPs identified on three or four of the branches of the species tree, leading to STs 1, 23, 37, 47 and 62.

| **SNP position** | **Type of SNP** | **Base change** | **Gene (Corby/**  **Paris)** | **Product** | **Branches leading to** |
| --- | --- | --- | --- | --- | --- |
| *Homoplasic on four branches* | | | | | |
| 1025688 | synon | T->C | *LPC_2453/lpp0904* | toluene tolerance protein Ttg2B | ST1, ST37, ST47 and ST62 |
| 1035006 | synon | T->C | *LPC_2442/lpp0915* | transcriptional regulator FleQ | ST1, ST37, ST47 and ST62 |
| 1035015 | synon | G->A | *LPC_2442/lpp0915* | transcriptional regulator FleQ | ST1, ST37, ST47 and ST62 |
| 1035033 | synon | G->A | *LPC_2442/lpp0915* | transcriptional regulator FleQ | ST1, ST37, ST47 and ST62 |
| 1061079 | nonsynon | T->C | *LPC_2413/lpp0942* | diguanylate kinase (GGDEF domain) | ST1, ST37, ST47 and ST62 |
| 1061164 | synon | A->G | *LPC_2413/lpp0942* | diguanylate kinase (GGDEF domain) | ST1, ST37, ST47 and ST62 |
| 1081231 | synon | T->C | *LPC_2394/ lpp0960* | A/G specific adenine glycosylase | ST1, ST37, ST47 and ST62 |
| *Homoplasic on three branches* | | | | | |
| 578994 | synon | C->T | *LPC_2858/lpp0550* | adenylosuccinate synthetase, (PurA) | ST1, ST23, and ST47 |
| 694526 | synon | T->C | *LPC_2735/lpp0624* | hypothetical protein | ST1, ST47 and ST62 |
| 695083 | nonsynon | T->C | *LPC_2735/lpp0624* | hypothetical protein | ST1, ST47 and ST62 |
| 695464 | synon | A->T | *LPC_2734/lpp0625* | spore maturation protein A | ST1, ST47 and ST62 |
| 798230 | synon | T->A | *LPC_2649/ lpp0699* | conserved C-terminal part of RTX protein | ST1, ST47 and ST62 |
| 798242 | synon | G->A | *LPC_2649/ lpp0699* | conserved C-terminal part of RTX protein | ST1, ST47 and ST62 |
| 798245 | synon | T->A | *LPC_2649/ lpp0699* | conserved C-terminal part of RTX protein | ST1, ST47 and ST62 |
| 798260 | synon | T->A | *LPC_2649/ lpp0699* | conserved C-terminal part of RTX protein | ST1, ST47 and ST62 |
| 798261 | nonsynon | G->C | *LPC_2649/ lpp0699* | conserved C-terminal part of RTX protein | ST1, ST47 and ST62 |
| 857435 | nonsynon | A->G | *LPC_2602/lpp0747* | ABC type dipeptide/oligopeptide/nickel transport, | ST1, ST47 and ST62 |
| 885272 | nonsynon | A->G | *LPC_2582/lpp0766* | imidazolonepropionase, (HutI) | ST1, ST47 and ST62 |
| 973922 | synon | G->A | *LPC_2502/lpp0854* | L-serine dehydratase, (Sdh) | ST1, ST47 and ST62 |
| 988058 | nonsynon | C->A | *LPC_2491/lpp0866* | choloylglycine hydrolase/Peptidase C59 family protein | ST1, ST47 and ST62 |
| 988285 | synon | A->G | *LPC_2491/lpp0866* | choloylglycine hydrolase/Peptidase C59 family protein | ST1, ST47 and ST62 |
| 988339 | nonsynon | T->G | *LPC_2491/lpp0866* | choloylglycine hydrolase/Peptidase C59 family protein | ST1, ST47 and ST62 |
| 988801 | nonsynon | G->A | *LPC_2490/lpp0867* | phosphoenolpyruvate synthase, (PpsA) | ST1, ST47 and ST62 |
| 989107 | nonsynon | T->A | *LPC_2490/lpp0867* | phosphoenolpyruvate synthase, (PpsA) | ST1, ST47 and ST62 |
| 993326 | synon | G->A | *LPC_2488/lpp0869* | nicotinate-nucleotide pyrophosphorylase, (NadC) | ST1, ST47 and ST62 |
| 993691 | synon | A->G | *LPC_2487/lpp0870* | N-acetylglucosaminyltransferase, (MurG) | ST1, ST47 and ST62 |
| 993901 | synon | T->C | *LPC_2487/lpp0870* | N-acetylglucosaminyltransferase, (MurG) | ST1, ST47 and ST62 |
| 993949 | synon | C->T | *LPC_2487/lpp0870* | N-acetylglucosaminyltransferase, (MurG) | ST1, ST47 and ST62 |
| 1020223 | nonsynon | T->G | *LPC_2461/lpp0896* | anthranilate phosphoribosyltransferase, (TrpD) | ST1, ST47 and ST62 |
| 1021056 | synon | G->A | *LPC_2459/lpp0898* | ABC transporter, ATP binding protein, (LptB) | ST1, ST23 and ST37 |
| 1023474 | synon | G->A | *LPC_2455/lpp0902* | polysialic acid capsule expression protein, (kdsD) | ST1, ST23 and ST37 |
| 1023522 | synon | A->C | *LPC_2455/lpp0902* | polysialic acid capsule expression protein, (kdsD) | ST1, ST23 and ST37 |
| 1024718 | synon | A->G | *LPC_2454/lpp0903* | toluene tolerance ABC transporter, (Ttg2A) | ST1, ST23 and ST37 |
| 1026117 | synon | C->T | *LPC_2453/lpp0904* | toluene tolerance protein, (Ttg2B) | ST1, ST23 and ST37 |
| 1042356 | nonsynon | G->T | *LPC_2433/lpp0923* | cytochrome c-type biogenesis protein, (CcmF) | ST1, ST23 and ST37 |
| 1042572 | synon | G->A | *LPC_2432/lpp0924* | cytochrome C biogenesis protein, (CcmG) | ST1, ST23 and ST37 |
| 1042596 | synon | T->C | *LPC_2432/lpp0924* | cytochrome C biogenesis protein, (CcmG) | ST1, ST23 and ST37 |
| 1042692 | synon | G->A | *LPC_2432/lpp0924* | cytochrome C biogenesis protein, (CcmG) | ST1, ST23 and ST37 |
| 1042749 | synon | A->G | *LPC_2432/lpp0924* | cytochrome C biogenesis protein, (CcmG) | ST1, ST23 and ST37 |
| 1042767 | synon | C->T | *LPC_2432/lpp0924* | cytochrome C biogenesis protein, (CcmG) | ST1, ST23 and ST37 |
| 1055741 | synon | G->A | *LPC_2418/lpp0937* | NAD(P) transhydrogenase subunit beta, (PntB) | ST1, ST23 and ST37 |
| 1060955 | nonsynon | C->G | *LPC_2413/lpp0942* | diguanylate kinase (GGDEF domain) | ST1, ST47 and ST62 |
| 1061021 | nonsynon | C->T | *LPC_2413/lpp0942* | diguanylate kinase (GGDEF domain) | ST1, ST47 and ST62 |
| 1078092 | synon | G->A | *LPC_2397/lpp0957* | hypothetical protein, Sel-1 repeat protein | ST1, ST23 and ST37 |
| 1081123 | synon | C->T | *LPC_2394/lpp0960* | A/G specific adenine glycosylase, (MutY) | ST1, ST37 and ST62 |
| 1081129 | synon | T->C | *LPC_2394/lpp0960* | A/G specific adenine glycosylase, (MutY) | ST1, ST37 and ST62 |
| 1081513 | synon | A->T | *LPC_2393/lpp0961* | conserved hypothetical protein, (AsmA) | ST37, ST47 and ST62 |
| 1086849 | intergenic | G->A | intergenic | N/A | ST1, ST37 and ST62 |
| 2717362 | intergenic | A->G | intergenic | N/A | ST1, ST37 and ST62 |

synon, synonymous

nonsynon, nonsynonymous
