# Supplementary material for: Multiple major disease-associated clones of Legionella pneumophila have emerged recently and independently
Source: Genome Res. 2016 Nov;26(11):1555–64. doi: 10.1101/gr.209536.116 (PMC5088597; doi:10.1101/gr.209536.116)
Supplement: Supplemental Material [file supp_gr.209536.116_Supplemental_Table_S11.docx]

Table S11. Genes that are significantly more similar in the five major disease-associated STs than is expected, given the similarity of the five STs across all core genes and also with respect to the conservation of each gene across the species.

| **Gene** | **Other name** | **Product/function** |
| --- | --- | --- |
| *lpp0536* | *poxF* | phenol hydroxylase |
| *lpp0542* | *rpoN* | RNA polymerase signma-54 factor RpoN |
| *lpp0548* | *hflK* | protease subunit HflK specific for phage lambda cII repressor |
| *lpp0550* | *purA* | adenylosuccinate synthetase (IMP-aspartate ligase) (AdSS) (AMPSase) |
| *lpp0561* | *ctpA* | carboxy-terminal protease |
| *lpp0615* |  | hypothetical protein |
| *lpp0618* |  | stearoyl-CoA-9-desaturase |
| *lpp0619* |  | hypothetical protein |
| *lpp0626* | *spmB* | spore maturation protein B |
| *lpp0627* |  | peptidase, M23/M37 family |
| *lpp0643* | *fthC* | 5-formyltetrahydrofolate cyclo-ligase |
| *lpp0653* | *sufC* | ATP transporter, ABC binding component, ATP-binding protein |
| *lpp0655* | *sufS/csdB* | selenocysteine lyase |
| *lpp0658* | *lysS* | lysyl tRNA synthetase |
| *lpp0661* | *phtB* | major facilitator family transporter |
| *lpp0665* |  | hypothetical protein |
| *lpp0676* |  | transmembrane protein |
| *lpp0677* |  | conserved hypothetical protein |
| *lpp0679* |  | hypothetical protein conserved within *Legionellae* |
| *lpp0680* | *comA* | DNA uptake/competence protein ComA |
| *lpp0707* | *phtF* | major facilitator transporter PhtF |
| *lpp0757* | *tdh* | threonine(-3-)dehydrogenase |
| *lpp0758* |  | ABC transporter ATP-binding protein |
| *lpp0759* | *enhA* | enhanced entry protein EnhA |
| *lpp0760* |  | predicted transporter component (contains sulphur transport domain) |
| *lpp0761* |  | predicted transporter component |
| *lpp0801* |  | DNA helicase, SNF2/RAD54 family domain protein |
| *pp0810* | *lipA* | lipoic acid synthetase |
| *lpp0865* |  | acyl CoA dehydrogenase, short chain specific |
| *lpp0866* |  | choloylglycine hydrolase/Peptidase C59 family |
| *lpp0867* | *ppsA* | phosphoenolpyruvate synthase |
| *lpp0874* | *mreC* | rod shape determining protein MreC |
| *lpp0877* |  | hypothetical protein conserved within Legionellae |
| *lpp0878* | *icd* | isocitrate dehydrogenase, NADP-dependent |
| *lpp0880* | *clpA* | ATP binding protease component ClpA |
| *lpp0883* |  | lipopolysaccharide biosynthesis glycosyltransferase |
| *lpp0887* |  | peptidase, M23/M37 family |
| *lpp0888* | *xseA* | exonuclease VII, large subunit |
| *lpp0890* |  | periplasmic protein |
| *lpp0891* |  | diguanylate cyclase/phosphodiesterase, GGDEF and EAL domain |
| *lpp0892* |  | conserved hypothetical protein |
| *lpp0893* |  | flavin containing monooxygenase |
| *lpp0907* | *rsbV* | conserved hypothetical protein |
| *lpp0911* | *lolD* | ABC transporter, ATP binding protein |
| lpp0890 |  | periplasmic protein |
| *lpp0913* |  | membrane fusion protein |
| *lpp0914* |  | hypothetical protein conserved within Legionellae |
| *lpp0918* | *ccmA* | heme exporter protein CcmA |
| *lpp0920* | *ccmC* | heme exporter protein CcmC |
| *lpp0922* | *ccmE* | cytochrome c-type biogenesis protein CcmE |
| *lpp0931* | *acdA* | acyl CoA dehydrogenase, short chain specific |
| *lpp0932* |  | 3-hydroxyisobutyryl Coenzyme A hydrolase |
| *lpp0933* |  | enoyl-CoA hydratase/carnithine racemase |
| *lpp0934* |  | hypothetical protein |
