# Supplementary material for: Multiple major disease-associated clones of Legionella pneumophila have emerged recently and independently
Source: Genome Res. 2016 Nov;26(11):1555–64. doi: 10.1101/gr.209536.116 (PMC5088597; doi:10.1101/gr.209536.116)
Supplement: Supplemental Material [file supp_gr.209536.116_Supplemental_Table_S12.docx]

Table S12. Recombination events that occurred on the branches leading to the ST47 or ST37 lineages. The start and end of the regions are with respect to the ST47 and ST37 mapping references (Lorraine and EUL 132, respectively).

| **Region start** | | **Region end** | **Length** | **Top hit** | **% identity of best hit** |
| --- | --- | --- | --- | --- | --- |
| *Recombined regions detected on the branch leading to ST47* | | | | | |
| 530,564 | 629,905 | | 99,341 | ST62 | 100 |
| 636,480 | 711,192 | | 74,712 | ST62 | 100 |
| 719,451 | 765,675 | | 46,224 | ST62 | 100 |
| 772,825 | 820,139 | | 47,314 | ST62 | 100 |
| 848,400 | 850,454 | | 2,054 | ST62 | 100 |
| 888,240 | 985,915 | | 97,675 | ST62 | 100 |
| 990,561 | 1,006,506 | | 15,945 | ST62 | 100 |
| 1,517,688 | 1,521,080 | | 3,392 | ST84 | 99.59 |
| 1,917,592 | 1,946,487 | | 28,895 | No donor found | NA |
| 1,990,956 | 2,002,446 | | 11,490 | ST78/ST62 | 98.88 |
| 2,141,795 | 2,143,409 | | 1,614 | ST44 | 99.38 |
| 2,623,816 | 2,625,196 | | 1,380 | ST62 | 100 |
| *Recombined regions detected on the branch leading to ST37* | | | | | |
| 2,063,553 | | 2,074,733 | 11,180 | ST74 | 98.09 |
| 2,183,734 | | 2,274,312 | 90,578 | Paris (ST1) & ST5 | 100 |
