# Supplementary material for: Multiple major disease-associated clones of Legionella pneumophila have emerged recently and independently
Source: Genome Res. 2016 Nov;26(11):1555–64. doi: 10.1101/gr.209536.116 (PMC5088597; doi:10.1101/gr.209536.116)
Supplement: Supplemental Material [file supp_gr.209536.116_Supplemental_Fig_S4.pdf]

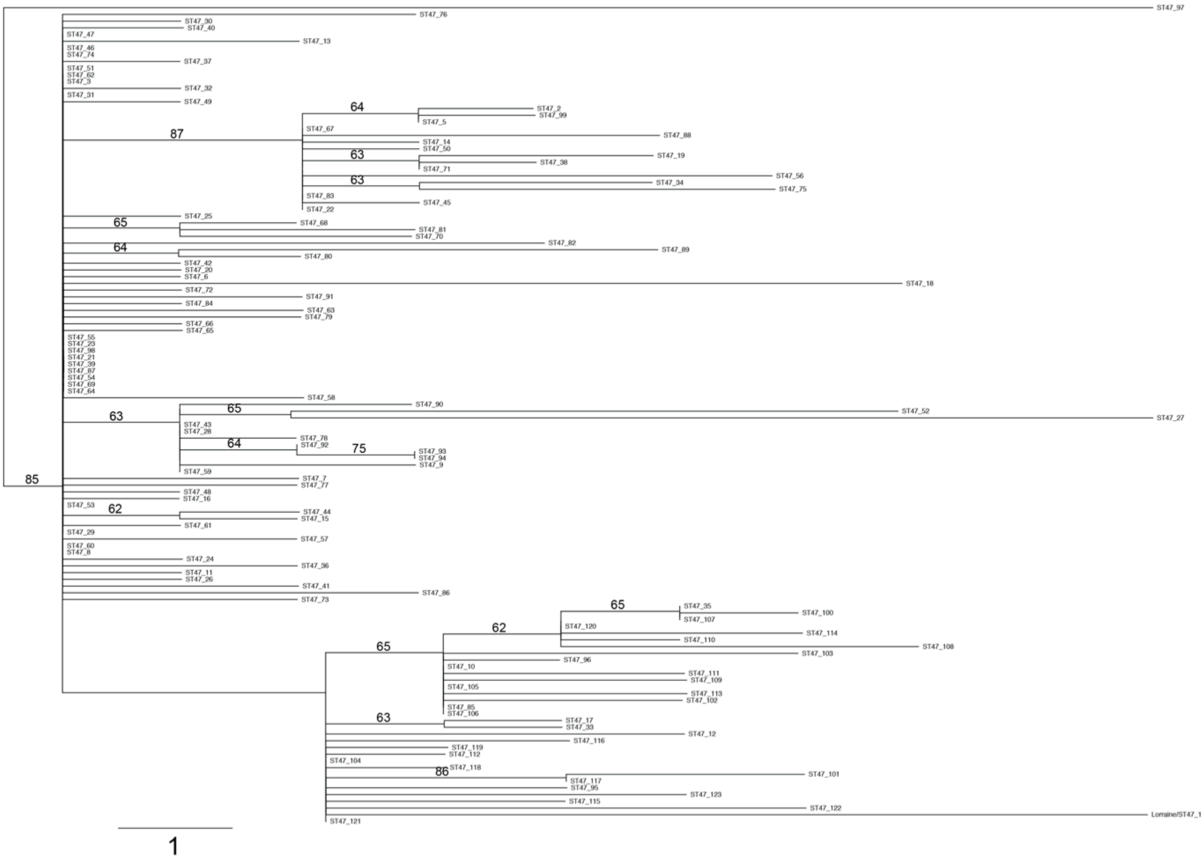

ST47_97
ST47_76
ST47_30
ST47_40
ST47_47
ST47_13
ST47_46
ST47_74
ST47_37
ST47_51
ST47_62
ST47_3
ST47_32
ST47_31
ST47_49
64
ST47_2
ST47_99
ST47_5
87
ST47_67
ST47_88
ST47_14
ST47_50
63
ST47_19
ST47_38
ST47_71
ST47_56
63
ST47_34
ST47_75
ST47_83
ST47_45
ST47_22
ST47_25
65
ST47_68
ST47_81
ST47_70
ST47_82
64
ST47_89
ST47_80
ST47_42
ST47_20
ST47_6
ST47_18
ST47_72
ST47_91
ST47_84
ST47_63
ST47_79
ST47_66
ST47_65
ST47_55
ST47_23
ST47_98
ST47_21
ST47_39
ST47_87
ST47_54
ST47_69
ST47_64
ST47_58
ST47_90
65
ST47_52
63
ST47_27
ST47_43
ST47_28
ST47_78
64
ST47_92
75
ST47_93
ST47_94
ST47_9
ST47_59
85
ST47_7
ST47_77
ST47_48
ST47_16
ST47_53
62
ST47_44
ST47_15
ST47_61
ST47_29
ST47_57
ST47_60
ST47_8
ST47_24
ST47_36
ST47_11
ST47_26
ST47_41
ST47_86
ST47_73
65
ST47_35
ST47_100
ST47_107
62
ST47_120
ST47_114
ST47_110
65
ST47_108
ST47_103
ST47_96
ST47_10
ST47_111
ST47_109
ST47_105
ST47_113
ST47_102
ST47_85
ST47_106
63
ST47_17
ST47_33
ST47_12
ST47_116
ST47_119
ST47_112
ST47_104
ST47_118
86
ST47_101
ST47_117
ST47_95
ST47_123
ST47_115
ST47_122
Lorraine/ST47_1
ST47_121
1
